# The Influence of Exposure to Nature on Inpatient Hospital Stays: A Scoping Review

**DOI:** 10.1177/19375867231221559

**Published:** 2024-01-30

**Authors:** Keegan Guidolin, Flora Jung, Sarah Hunter, Han Yan, Marina Englesakis, Stephen Verderber, Sami Chadi, Fayez Quereshy

**Affiliations:** 1 Department of Surgery, University of Toronto, Canada; 2 Institute of Biomedical Engineering, University of Toronto, Canada; 3 Department of Medicine, University of Toronto, Canada; 4 Institute of Health Policy, Management, and Evaluation, University of Toronto, Canada; 5 Department of Family and Community Medicine, University of Toronto, Canada; 6 Library and Information Services, University Health Network, Toronto, Canada; 7 Dalla Lana School of Public Health, University of Toronto, Canada; 8 John H. Daniels Faculty of Architecture, Landscape and Design, University of Toronto, Canada; 9 Centre for Design + Health Innovation, Toronto, Canada; 10 Department of Surgery, University Health Network, Toronto, Canada

**Keywords:** biophilia, inpatient hospitals, access to nature, natural light, medical/surgical unit, hospital

## Abstract

**Aim:**

To summarize the existing literature surrounding the influence of natural elements on course in hospital and to introduce clinicians to the concept of biophilic design and the potential for incorporation of nature into the hospital environment as a component of a therapeutic hospitalization.

**Background:**

For decades, architects and designers have espoused the benefits of incorporating natural elements into the healthcare environment for therapeutic purposes. The benefits of this “biophilic” design philosophy has been investigated predominantly in long-term care or rehabilitation settings; however, some of the most appealing opportunities lie in the acute care setting.

**Methods:**

This scoping review surveyed the literature surrounding the influence of exposure to nature on course in acute hospitalizations. After screening 12,979 citations, 41 articles were included. Exposures were divided into seven categories, the most common of which were the presence of a window/natural light, a natural scene through a window, and nature soundscapes. These articles were reviewed in a narrative fashion and thematic analysis was conducted.

**Results:**

Studies were extremely heterogeneous in their design, research questions, and reported outcomes. Types of exposure to nature studied were exposure to a real natural scene through a window, presence of a window/nature light, nature in the healthcare environment, art depicting nature, direct contact with nature, nature soundscapes, and nature experienced through virtual reality (VR).

**Conclusions:**

Exposure to nature during an acute hospital admission appears to have a real but small therapeutic effect, predominantly on psychological metrics like anxiety/depression, pain, and patient satisfaction. Greater beneficial effects are seen with greater durations of exposure to nature and greater degrees of immersion into nature (e.g., creating multisensory experiences using emerging technology like VR).

## Introduction

The term biophilia was first coined by the psychoanalyst Eric Fromm in his book *The Anatomy of Human Destructiveness* to describe the love of life and of living things (Fromm, 1973) and was adopted as a concept later by biologist and ecologist E. O. Wilson in his book *Biophilia* and states that all humans, by virtue of genetic heritage, share an affinity for the natural world (Kellert et al., 2011; Wilson, 1984). This notion has served to inspire architects and designers to incorporate natural elements into the design of the built environment to conduct “biophilic design”(Kellert & Wilson, 1993). Biophilic design has been described as consisting primarily of two basic dimensions: the naturalistic dimension (shapes and forms in the built environment that reflect the human affinity for nature) and the vernacular dimension (buildings and landscapes that connect to the ecology of a region; Kellert et al., 2008). This has been more recently expressed by Kellert and Calabrese (2015) as a framework with 24 parameters in three groups and by Browning et al. (2014) as a framework of 14 parameters in three groups.

Within the biophilic design movement, natural elements are often thought to have salutary (health positive) effects; thus, architects of healthcare facilities have sought to use these biophilic elements and attributes to design therapeutic spaces (Franklin, 2012). This has been codified in part by the “evidence-based design” movement within architecture, which seeks to empirically assess and understand the effects of design on the desired outcome of the designed space. In the context of healthcare, scientifically founded designs include modifications to reduce hospital noise, prevent the spread of infectious agents, reduce stress and fatigue, and more (Ulrich, 2006). In 1984, Ulrich published a study comparing patients who had undergone surgery, half of whom stayed in a room with a view of deciduous trees, while the other half stayed in a room with a view of a brick wall and found that those exposed to a view of trees required less analgesia and had a reduced length of stay (Ulrich, 1984). This finding garnered interest in the potential for inexpensive interventions (e.g., artificial plants, images of landscapes, or natural soundscapes, etc.) to improve patient outcomes. If truly effective, such interventions could prove to be simple, inexpensive changes to help combat complex problems.

The objective of this study was to synthesize the existing literature on the effects of exposure of hospital inpatients to natural elements on conventionally studied health outcomes such as morbidity, mortality, length of stay, and analgesic use.

## Method

### Literature Search and Study Selection

We conducted a librarian-led search of Medline (Ovid), Medline ePub ahead of print/Medline in-process and other nonindexed citations, Embase (Ovid), Cochrane Central Register of Controlled Trials (Ovid), Cochrane Database of Systematic Reviews (Ovid), APA PsycINFO (Ovid), Ovid Emcare Nursing (Ovid), Web of Science (Clarivate, consisting of Science Citation Index Expanded, Social Sciences Citation Index, Arts & Humanities Citation Index, and Emerging Sources Citation Index), Scopus (Elsevier), CINAHL (EbscoHost), Avery Index (ProQuest), and Design and Applied Arts Index (ProQuest; Online Appendix 1). The search strategy was constructed and iteratively revised by the authors with the aim of being as sensitive as possible. Resulting citations were imported into Covidence systematic review software (Veritas Health Innovation, Melbourne Australia) for subsequent screening.

Inclusion criteria were studies (i) reporting original data, (ii) reporting on any patient outcome, (iii) reporting on patients admitted to an acute healthcare facility, and (iv) investigating the influence of exposure to nature. Studies in the setting of outpatient clinics, nursing homes, retirement homes, rehabilitation facilities, and other long-term care facilities were excluded, as were studies on outpatients or nonpatients, and studies not investigating nature-based design.

Abstracts underwent single reviewer screening. Included citations then underwent full-text review for inclusion independently by two reviewers. Full-text inclusion conflicts were resolved by a third reviewer. Data were then extracted (see Online Appendix 2 for data collection form) and subjected to thematic analysis and narrative summary.

## Results

### Cohort Build and Study Characteristics

**Figure 1.**
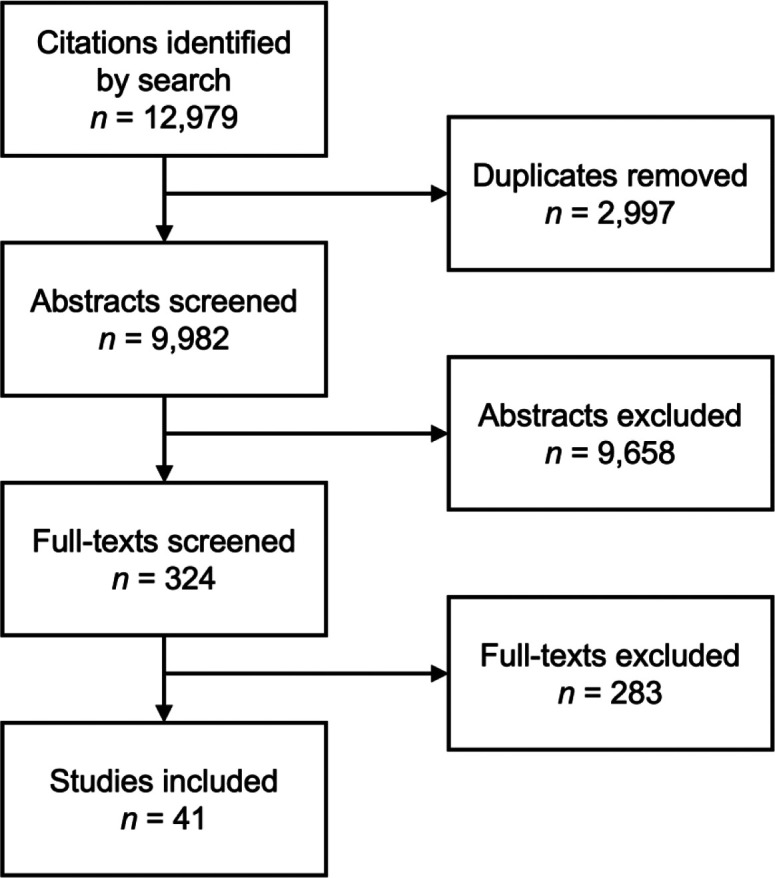
Preferred Reporting Item for Systematic Reviews and Meta-Analyses (PRISMA) flow diagram of included studies.

**Figure 2.**
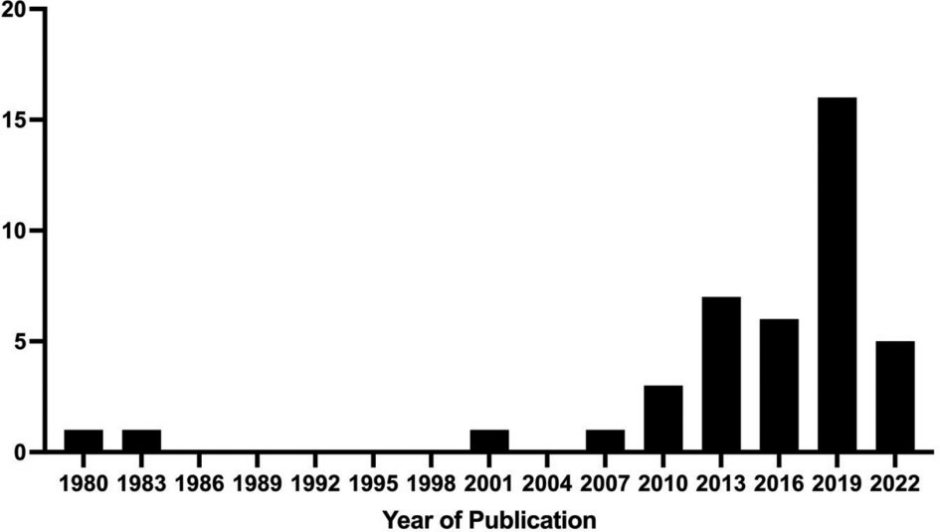
Histogram of year of publication of included studies. *Note*. Not all databases searched cover resources from the entire time period displayed here.

**Figure 3.**
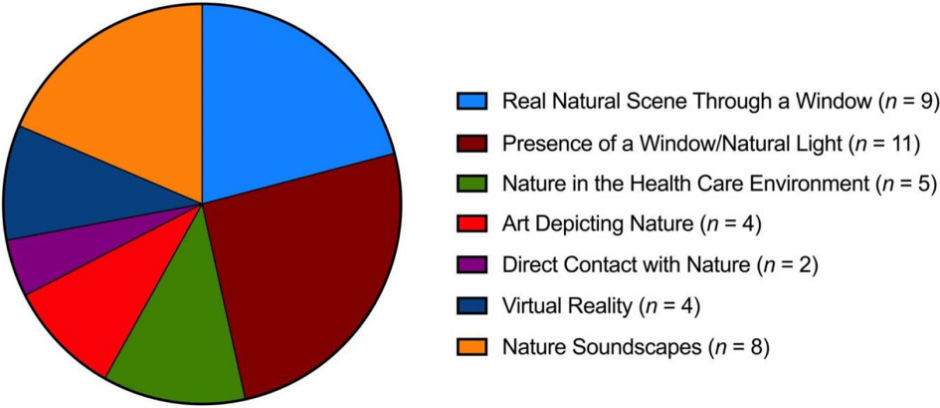
Proportional representation of the types of exposures to nature studied (*n* = 43).

Our initial search produced 12,979 citations; after abstract and full-text screening, 41 articles were ultimately included in the study (Figure 1). Most studies were conducted in North America (United States); however, studies were well distributed between Europe, Asia, and the Middle East. Most studies were either randomized controlled trials (RCTs) or retrospective cohort studies (Table 1), and most were published in the last 10 years (Figure 2). Roughly equal numbers of studies were conducted in hospital ward environments and intensive care units (ICU; Table 2). The most studied exposures to nature were through the presence of windows/daylight, a view of a real natural scene through a window, and by exposure to natural soundscapes (Table 2 and Figure 3). A complete list of all included studies can be found in Online Appendix 3.

**Table 1.** Study Demographics.

Geographic Region	
North America, *n*	14
Europe, *n*	9
Asia, *n*	8
Middle East, *n*	9
Oceania, *n*	1
Year of publication, median (IQR)	2018 (2013–2019)
Study Design	
Meta-analysis	1
Randomized controlled trial	13
Prospective cohort or single-arm study	5
Retrospective cohort study	12
Case series	2
Cross-sectional, survey-, or interview-based study	6
Time series	2

*Note*. IQR: interquartile range.

**Table 2.** Care Settings for Included Studies.

Care Setting	*n* (%)
Hospital, any inpatient location	2 (4.9)
Ward	21 (51.2)
General ward ^a^	8 (19.5)
Medical ward	3 (7.3)
Surgical ward	3 (7.3)
Geriatric ward	1 (2.4)
Pediatric ward	1 (2.4)
Psychiatric ward	3 (7.3)
Obstetrics ward	2 (4.9)
Intensive care unit	18 (43.9)
General intensive care unit	13 (31.7)
Cardiac intensive care unit	5 (12.2)

^a^ Found in hospitals without defined medical versus surgical wards.

### The Influence of a Real Natural Scene Through a Window

The effect of a view of a natural scene through a window is among the most studied means of exposure to nature. The landmark article published by Ulrich (1984) sought to assess the influence of the view through a hospital bed-adjacent window on recovery after open cholecystectomy. This study found that patients exposed to trees had a reduced length of stay, had fewer “negative comments” (a measure of patient symptoms/complaints), and reduced opioid requirements compared to patients exposed to a wall. The possibility that sensory stimuli could affect postoperative course sparked interest in subsequent studies.

Emami et al. (2018) conducted a prospective study of patients admitted to a room with a window view of either a garden or a street and measured anxiety and pain. They found that most patients with a garden view reported reductions in pain scores and reductions in anxiety. Wang et al. (2019) surveyed 296 postcaesarian section women and found that women who expressed satisfaction with the number of natural elements outside their bedside window had decreased frequency and severity of pain.

Several studies examined the effect of a view of nature through a window on outcomes like patient experience and perceived quality of care. Mihandoust et al. (2021) conducted a nationwide survey of 652 admitted patients and found that when patients had a view to the outdoors, they rated more highly the quality of the hospital, the quality of the care they received, and the quality of their room. This effect was exaggerated in patients with a view of “green spaces.” Zaki et al. (2016) also found improved patient satisfaction among 53 patients admitted to a psychiatric ward.

Timmermann et al. (2015) interviewed 12 inpatients who expressed that views of nature create positive thoughts and emotions that support a “sense of personal strength and well-being” during illness. They expressed the value of such views to foster a sense of inner peace and escapism from negative thoughts and act to create a sense of freedom in situations where freedom was otherwise restricted. Similarly, Anaker et al. (2019) interviewed 16 patients with stroke who expressed that the view of a nearby forest was calming and facilitated a distraction from the stress of hospitalization. They also expressed a desire for greater integration of nature into the ward itself in the form of potted plants, art depicting natural scenes, and an aquarium.

***Timmermann et al. (2015) interviewed 12 inpatients who expressed that views of nature create positive thoughts and emotions that support a “sense of personal strength and well-being” during illness.***

The therapeutic effect of views of nature through bedside windows has also been studied in ICU populations, predominantly focusing on ICU-specific outcomes. Kohn et al. (2013) conducted a study of 6,660 patients admitted to ICUs but found no differences between “nature view” and “industrial view” groups with respect to any important outcomes. Similarly, Shepley et al. (2012) compared 110 ICU patients treated in a unit either with or without access to natural light and garden views but found no important differences between groups.

### The Influence of Exposure to Windows and Natural Light

Some authors have asked whether access to natural light is physiologically important in a therapeutic environment. Common outcomes are delirium-related, given prior literature suggesting that exposure to natural light cycles can help to orient patients at risk of delirium (Groves, 2019; Lee et al., 2021).

Iwamoto et al. (2020) conducted a study comparing the rates of in-hospital falls in 2,767 patients admitted to hospital with a window-adjacent bed or a nonwindow-adjacent bed. They found a ∼50% reduction in falls in the group of patients with window-adjacent beds, even after adjustment for demographic and other important factors. Aomura et al. (2021) conducted a similar study investigating the therapeutic effect of daylight access in a population of 1,556 geriatric patients admitted to either a window-adjacent bed or a bed not adjacent to a window. They found no differences between groups with respect to rate of delirium related events, length of stay, ICU admission, or death.

Other studies of window/daylight exposure have specifically investigated the effect of different cardinal directions of window orientation (encompassing the duration, intensity, and timing of natural light). Choi et al. (2012) found that patients staying in ward rooms with a southeastern exposure (bright, morning light, *n* = 122) had a shorter length of stay than those staying in ward rooms with a northwestern exposure (dim, evening light, *n* = 100). Similar results were reported by Benedetti et al. (2001) in patients with bipolar disorder (*n* = 187). They showed a reduction in length of stay in patients admitted to east facing rooms that received direct daylight in the morning as opposed to west facing rooms receiving evening light.

Zaki et al. (2016) investigated the association between the intensity of daylight on a psychiatric ward on the quality of life and satisfaction scores of 53 patients and found that natural light levels >500 Lux were associated with increased patient satisfaction.

The influence of daylight exposure has been more thoroughly explored in ICU populations, in many cases, because of the hypothesized influence of natural light cycles on delirium; however, evidence is mixed. Arenson et al. (2013) compared 1,010 patients admitted to either ICU bays with or without windows but found no differences in ICU or in-hospital delirium. Similarly, Kohn et al. (2013) compared 6,336 patients admitted to ICU bays with or without windows, finding no differences between groups. Interestingly, a subgroup analysis suggested a protective effect of window exposure in patients with prolonged ICU stays. Finally, Wunsch et al. (2011) studied 789 patients admitted to ICU bays with or without windows, with subarachnoid hemorrhage and found no differences. The authors hypothesized that such patients may be “too comatose” to be influenced by sensory exposure.

Three studies in ICU populations have found beneficial effects of window exposure on various outcomes. Chiu et al. (2018) investigated a number of ICU-specific outcomes in a population of 281 patients admitted to a bay. They found that patients in bays with a window had a shorter length of stay in the ICU; however, like prior studies, they found no effect on incidence of delirium. Keep et al. (1980) studied 150 patients admitted to ICU bays and found improvements in rates of hallucination/delusions, orientation to time, sleep disturbances, and recall of the admission among patients exposed to windows. This study did not specifically measure the incidence of delirium. Similar results were reported by Smonig et al. (2019) who studied 195 patients admitted to the ICU and found no differences in the incidence or duration of delirium or a number of other ICU-specific outcomes based on window exposure but did find a reduction in hallucinations, agitation, and the need for antipsychotics among those exposed to windows.

### The Influence of Nature in the Healthcare Environment

Some researchers have attempted to integrate natural elements, both genuine and artificial, typically in the form of plants or flowers. Park and Mattson (2008) conducted an RCT in which they placed 12 potted plants in the rooms of 45 postappendectomy inpatients for 72 hr and compared various clinical outcomes to those of 45 control postappendectomy inpatients. They found that the presence of plants and flowers lowered blood pressure, heart rate, pain intensity, pain distress, anxiety, and fatigue. Park and Mattson (2008) found that patients exposed to potted plants while in hospital required fewer analgesics than unexposed patients and found their environment relaxing, comfortable, colorful, attractive, satisfying, pleasant smelling, and calming, with 93% reporting that the plants were the most positive quality of the room.

***Park and Mattson (2008) found that patients exposed to potted plants while in hospital required fewer analgesics than unexposed patients and found their environment relaxing, comfortable, colorful, attractive, satisfying, pleasant smelling, and calming, with 93% reporting that the plants were the most positive quality of the room.***

Allah Yar and Kazemi (2020) conducted a similar RCT in which they placed potted plants in the rooms of 27 pediatric inpatients for 72 hr and compared various clinical outcomes to controls. They found that patients with potted plants had more normal heart and respiratory rates than those without plants. This effect was also observable over time with reductions to normal in blood pressure, heart rate, and respiratory rate among those randomized to the potted plant group after 72 hr versus upon admission, whereas no such reductions over time were observed in the control group. Exposure to plants also improved all subjective indices measured: fear, relaxation, happiness, attention, compatibility, anxiety, and depression.

Koh et al. (2019) conducted a similar RCT on the effect of artificial elements in a population of patients admitted to hospital postacute coronary syndrome. They placed four artificial potted roses in patient rooms and compared anxiety and depression levels between patients with and without exposure to natural elements. Patients exposed to artificial flowers had a significantly lower depression score upon discharge and had a sustained reduction in depression score at 3 months. Upon discharge, 6.6% of patients in the artificial flower group met criteria for depression as compared to 24% in the group not exposed to artificial flowers.

Instead of placing natural elements directly into patient rooms, some researchers studied the effect of a generalized “greening” of the public areas of the ward, which appeared similarly effective. Ali Khan et al. (2016) conducted an RCT in which 135 patients were assigned to a “greened” ward compared to 135 control patients. The “greened” ward was decorated with plants and flowers throughout. Patients on the “greened” ward had more normal vital signs, less pain, used fewer opioid analgesics, and had a reduced length of stay. Ali Khan et al. (2016) found that patients on “greened” wards reported that the plants put them at ease, making them feel more relaxed, happy, “better,” and “more alive”; all patients expressed positive opinions of the plants.

***Ali Khan et al. (2016) found that patients on “greened” wards reported that the plants put them at ease, making them feel more relaxed, happy, “better,” and “more alive”; all patients expressed positive opinions of the plants.***

Another geriatric ward “greening” study was conducted by Berg et al. (2020). They added moss walls, plant walls, and potted plants throughout. They found that functional independence was improved after greening and that rates of functional decline decreased from 32.1% prior to greening to 11.5% after greening.

### The Influence of Art Depicting Nature

Some researchers have questioned whether depictures of nature could produce therapeutic effects. Pati et al. (2016) conducted an RCT in which they randomized 181 patients to either a standard room or one with an artificial view of the sky. They found no differences in clinical outcomes; however, patients in rooms with the artificial skylight expressed greater satisfaction. A post hoc subgroup analysis suggested that the artificial skylight lowered stress and anxiety among patients that did not require analgesia.

Pearson et al. (2019) studied the effect of a nature-themed window murals on physiologic measures in 90 pediatric ward inpatients. They found that patients in rooms with murals had a greater normalization of heart rate. They also found that murals depicting trees were most effective and normalized both heart rate and blood pressure.

Aburas et al. (2017) conducted an RCT in which they placed televisions that ran a looped slideshow of nature photographs in the rooms of 26 women undergoing labor and delivery but found no differences in important clinical outcomes.

Finally, Finkel et al. (2021) studied the perceptions of 45 patients admitted to a cardiac surgery ward with respect to the type of art. Finkel et al. (2021) found that natural landscapes were most positively perceived of any type of art, reminding patients of vacation, making them feel better, providing a “good distraction,” and improving mood, comfort, stress, satisfaction, and overall experience.

***Finkel et al. (2021) found that natural landscapes were most positively perceived of any type of art, reminding patients of vacation, making them feel better, providing a “good distraction,” and improving mood, comfort, stress, satisfaction, and overall experience.***

### Influence of Direct Contact With Nature

Some researchers have studied the effects of more direct contact with and immersion in nature with “horticultural therapy” (gardening) or nature walks. Such interventions have only been studied in “physically well” inpatient populations admitted for psychiatric indications. Bielinis et al. (2019) studied the effect of a one-time walk through a forest on the mood and anxiety state of 50 psychiatry patients. These patients improved in levels of anxiety, depression, fatigue, confusion, energy, and anger.

Lu et al. (2021) conducted a meta-analysis of Chinese studies on horticultural therapy (the planting and maintenance of plants) for patients with schizophrenia (*n* = 1,199). They found improvements in symptoms and quality of life. Effects were greater outside hospitals and scaled with exposure immersion and duration.

### Influence of Exposure to Nature Through Virtual Reality (VR)

Some authors have sought to achieve immersion in nature with the use of VR technologies, which can provide a highly portable, inexpensive, and immersive experience. Gao and Zhang (2020) studied several different VR models of patient rooms. They measured the skin conductance of 54 inpatients admitted to various hospital wards, finding that the presence of nature were the most effective at reducing skin conductance and most “restorative.”

Kucher et al. (2020) studied the effects of access to a VR nature experience on the levels of pain, nausea, and anxiety of six children undergoing major surgery. These patients reported that the VR experience was enjoyable, helpful, and usable, and effectively reduced pain, nausea, and anxiety, though one patient experienced nausea. They expressed a desire for a more immersive experience.

VR has also been explored for use in ICUs, where patients are relatively immobilized. Gerber et al. (2019) measured the effect of a 5-min VR nature experience on several clinical outcomes in 57 patients admitted to the ICU following cardiac surgery. They found a mild reduction in respiratory rate, and patients described the VR experience as highly usable, immersive, and satisfying. Similarly, Jawed et al. (2021) exposed 15 ICU patients to a VR beach setting with audio for 15 twice a day to study its feasibility and effects on agitation, anxiety, and confusion. They found that the VR sessions were tolerated in 80% of patients with 71% reporting improvements in anxiety levels, 60% requesting further such sessions. Jawed et al. (2021) found that the majority of ICU patients studied stated that they felt they were “more on the beach than in the ICU,” attesting to VR’s ability to provide a positive escape from the hospital environment.

***Jawed et al. (2021) found that the majority of ICU patients studied stated that they felt they were “more on the beach than in the ICU,” attesting to VR’s ability to provide a positive escape from the hospital environment.***

### Influence of Natural Soundscapes

One of the most robustly studied exposures is to auditory stimuli. Bahonar et al. (2019a) conducted an RCT in which they assigned 65 traumatic coma patients to experience either nature sounds for 30 min via headphones twice daily or not. They found that patients in the nature sound group recovered faster and had improved cognitive recovery. This group also conducted a second study (Bahonar et al., 2019b) on 60 traumatic coma patients in the ICU, this time investigating hemodynamic indices. They found that nature sounds resulted in a more normal heart rate than the control group at 2 weeks after initiation of the twice daily regimen; however, there were no differences in most indices.

Ghezeljeh et al. (2017) conducted a similar RCT on 93 patients admitted to the cardiac ICU through three groups. The first two groups either heard a nature soundscape or had noise cancelling headphones for 30 min per day for 2 days compared to a standard-of-care group. They measured physiologic indicators; however, no differences were detected between groups.

Saadatmand et al. (2013, 2015) conducted two RCTs of 60 mechanically ventilated patients admitted to the ICU with a Glasgow Coma Scale (GCS) of nine or higher, exposed to 90 min of nature sounds via a speaker in the ICU room versus no such exposure. They found a time-dependent reduction in perceived pain in the nature sounds group such that the magnitude of the analgesic effect increased to a plateau after 60 min. This pain relief was sustained for at least 30 min after the intervention ended. No such reduction was found in the control group. In their second study, they found that blood pressure was more normal in the nature sounds group at all time points during and after the nature sounds treatment. The nature sounds group also reported lower anxiety and agitation levels than the control group.

Bauer et al. (2011) conducted an RCT of 100 postcardiac surgery patients admitted to the ICU, receiving either standard care or exposed to a nature soundscape for 20 min twice per day for 3 days starting on postoperative Day 2. They found that patients exposed to nature sounds had a mild decrease in pain starting after the second session and that patients in the nature group reported a significantly higher level of relaxation.

Ghezeljeh et al. (2018) conducted an RCT on the effects of nature sounds on sleep quality in coronary care units among 93 patients allocated to routine care, silence via either noise-cancelling headphones or nature sounds (both two sessions of 30 min). The nature sounds group had an improved sleep depth, reduced sleep latency, fewer awakenings, faster return to sleep, improved subjective sleep quality, and improved total sleep quality. Importantly, there was no difference between the nature sounds group and the group exposed to silence, though the nature sounds group had a larger effect size.

Finally, Mackrill et al. (2013) surveyed 11 inpatients following cardiac operations for their opinions of the soundscape of the ward. They reported their enjoyment of natural sounds, for instance, the sound of birdsong and the sound of wind in the trees through open windows.

## Discussion

Despite the insistence of many architects, interior designers, and proponents of biophilic design, the link between exposure to nature and improvements in relevant clinical outcomes is tenuous, and where present, effect sizes appear small in the broader clinical contexts presented in these studies. Yet, there exist several well-designed studies that provide compelling evidence that certain types of exposure can confer meaningful positive effects on cognitively mediated outcomes, like pain perception, anxiety, mood, and perceived quality of care. Studies like Emami et al. (2018), Wang et al. (2019), Mihandoust et al. (2021), and Zaki et al. (2016) attest to the effect of a room with a view of nature on these outcomes. The need for a cognitive mediation of these effects is also borne out by the fact that studies of individuals with a decreased level of consciousness often see no effect (Bahonar et al., 2019a, 2019b; Wunsch et al., 2011), though this could also be because such patients are generally more unwell and effect sizes would have to be large to be observed.

The presence of windows and natural daylight was most often investigated with respect to delirium-related outcomes, likely because access to means of reorientation to time and place are frequently cited interventions to prevent delirium (Groves, 2019; Lee et al., 2021). The vast majority of these studies found no improvement in delirium-related outcomes among patients with access to daylight (Aomura et al., 2021; Arenson et al., 2013; Chiu et al., 2018; Kohn et al., 2013; Smonig et al., 2019); however, the study by Iwamoto et al. (2020) found a ∼50% reduction in falls in patients admitted to a window-bed. Whether this effect was mediated by a delirium state in this study is uncertain as the authors did not measure delirium. Access to daylight has been demonstrated by Keep et al. (1980) and Smonig et al. (2019) to reduce particular symptoms of delirium, namely hallucinations and agitation, and to reduce the need for antipsychotic medications.

Researchers have empirically identified the importance of adequately windowed conditions and meaningful contact with nature in inpatient settings closely allied with acute care hospital environments. Verderber et al. (Verderber, 2016; Verderber & Reuman, 1987; Verderber et al., 1987) surveyed 250 staff and patients in six rehabilitation hospitals and found that poorly windowed conditions were tantamount to architecturally unwindowed conditions. Nature was a preferred theme, as were nature-view surrogates in the form of plants and wall murals in therapy treatment areas.

The other interesting phenomenon revealed by the studies on access to daylight is the effect of the direction of the daylight exposure: east-facing windows (providing morning daylight) shortened length of stay in two studies by Choi et al. (2012) and Benedetti et al. (2001) This phenomenon has been insufficiently studied to propose a realistic mechanistic explanation, though the authors cite enhanced regulation of the patient’s circadian rhythm; significantly more work is required in this area to confirm these findings and establish the scientific mechanism whereby rate of recovery from illness is enhanced.

Among the most convincing studies are those that bring the patient into close contact with nature. Introducing natural elements into patient rooms or public areas of the ward has been consistently shown to reduce pain, anxiety, and depression and help to normalize vital signs (Ali Khan et al., 2016; Allah Yar & Kazemi, 2020; Koh et al., 2019; Park & Mattson, 2008). The effect is dampened when less immersive modes of exposure are used (e.g., artistic depictions; Aburas et al., 2017; Pearson et al., 2019), though patients still enjoy these natural elements (Finkel et al., 2021). Conversely, the effect seems to be amplified by increased immersion and exposure as seen in the studies by Bielinis et al. (2019) and Lu et al. (2021) which provided complete immersion in nature for an extended period of time. Thus, it may be the case that—like any other pharmaceutical or treatment—the dose matters, and for exposure to nature to have a meaningful therapeutic effect, patients must be as immersed as possible for as long as possible. In pharmaceutical medicine, this is known as the dose–response relationship.

***…like any other pharmaceutical or treatment—the dose matters, and for exposure to nature to have a meaningful therapeutic effect, patients must be as immersed as possible for as long as possible.***

VR technologies provide an exciting new means of delivering such immersive and extended exposures; however, existing studies are largely limited to feasibility and tolerability outcomes (the major concern being VR induced nausea). Perhaps the most promising VR-based study is by Jawed et al. (2021), which demonstrated that an immersive VR experience of nature is not only feasible and tolerable but also effectively reduces anxiety levels, replicating the effects seen in studies of exposure to real natural elements.

The influence of a nature soundscape has been highly investigated in several RCTs to similar results, suggesting a similar, cognitively based mechanism of action. In several trials, nature soundscapes helped to reduce pain, anxiety, and agitation, while helping to normalize vital signs and promoting relaxation (Bahonar et al., 2019a, 2019b; Bauer et al., 2011; Saadatmand et al., 2013, 2015). Interestingly, greater effects were seen in trials with longer durations of exposure to nature soundscapes, again suggesting a “dose-dependent” response to this type of nature exposure.

It is worth noting that VR and the soundscape of a space may not be considered a traditional component of the “built environment”; however, they may form part of a patient’s experienced environment. In addition, while VR environments are not built physically, those included in this study were designed intentionally and built virtually. Similarly, the soundscapes to which patients in these studies were exposed were designed intentionally to emulate the aural experience of being immersed in a natural environment. In addition, the foundations of biophilic design support their consideration as part of constructing a therapeutic environment, since both VR and nature soundscapes can be considered indirect experiences of nature under the framework proposed by Kellert and Cabrese (2015) or as natural analogues and nonvisual connections with nature under the framework proposed by Browning et al. (2014).

It must be acknowledged that across studies, whenever surveys or interviews are conducted, patients overwhelmingly prefer the presence of nature in the healthcare environment. While this preference does not necessarily have any “clinical value,” studies like Mihandoust et al. (2021) and Zaki et al. (2016) suggest that metrics of hospital quality may be tied to such preferences, which in turn may be valuable in certain models of healthcare.

In addition to the data presented here, a large volume of prior work exists that recapitulates the salutary effects of biophilic design in broader healthcare contexts, such as rehabilitation institutes, long-term care facilities, hospices, and mental health institutions (i.e., environments where patients may be admitted for longer periods of time), among not only patients but also those who work in those same environments (Abdelaal & Soebarto, 2019; Clarke and Currie, 2009; Laursen et al., 2014; Moore et al., 2018; Soderback et al., 2004; Tambyah et al., 2022; Tekin et al., 2022; Vujcic et al., 2017; Wastberg et al., 2021). The present study provides a novel perspective of biophilic design in the healthcare environment and helps to highlight those contexts and methods in which biophilic design might best be used in an acute care setting. This notably includes contexts like ICUs or other long-term stay units and highly immersive methods such as VR. The findings of this article also call upon intensivists and other inpatient clinicians to use biophilic design principles as tools wherever possible to improve the therapeutic environment in which they work.

This study represents a thorough summary of the published literature on the effects of natural elements on the hospital course of patients admitted to acute care facilities; however, it has several limitations that should be discussed. Because of space limitations, our manuscript did not explicitly discuss all outcomes investigated in the included studies, and only highlights positive results and notable negative results, though more data were extracted and analyzed. In addition, due to the high degree of heterogeneity in study design and outcome reporting, meta-analysis could not be completed. Similarly, because of the broad nature of the research question, a systematic review could not be conducted. We felt that a systematic review of any one of the types of exposure or outcomes would necessarily neglect a portion of the published literature. Because our goal was to investigate whether exposure to “nature”—in any sense—could produce health benefits in hospital inpatients, we felt that a scoping review format was most appropriate as it allowed for the breadth of scope required.

## Conclusion

A synthesis of the existing literature suggests that the therapeutic effect of exposure to nature on patients admitted to hospital is real but dose-dependent and of relatively low “potency”. Future studies seeking to investigate this effect should utilize high immersion, long duration exposures. An exciting emerging tool that could be used in both the investigation and delivery of this therapeutic effect is VR. This technology has the potential to create a highly immersive audio–visual experience that could be maintained, theoretically, for an indefinite period of time. It is important to neither overstate nor neglect the therapeutic value of exposure to nature in the delivery of healthcare to hospitalized patients.

## Implications for Practice

- Exposure to natural elements in the healthcare environment seems to have a real but modest effect on traditional health outcomes in acute care settings.
- The effect of exposure to natural elements can be conceptualized using a dose–response relationship as with pharmaceuticals.
- Both duration of exposure and immersion are important aspects of biophilic design.
- Emerging technologies like VR may facilitate increased exposure to natural elements in the absence of large-scale architectural renovations/changes.

## Supplemental Material

Supplemental Material, sj-docx-1-her-10.1177_19375867231221559 - The Influence of Exposure to Nature on Inpatient Hospital Stays: A Scoping Review Supplemental Material, sj-docx-1-her-10.1177_19375867231221559 for The Influence of Exposure to Nature on Inpatient Hospital Stays: A Scoping Review by Keegan Guidolin, Flora Jung, Sarah Hunter, Han Yan, Marina Englesakis, Stephen Verderber, Sami Chadi and Fayez Quereshy in HERD: Health Environments Research & Design Journal

Supplemental Material, sj-docx-2-her-10.1177_19375867231221559 - The Influence of Exposure to Nature on Inpatient Hospital Stays: A Scoping Review Supplemental Material, sj-docx-2-her-10.1177_19375867231221559 for The Influence of Exposure to Nature on Inpatient Hospital Stays: A Scoping Review by Keegan Guidolin, Flora Jung, Sarah Hunter, Han Yan, Marina Englesakis, Stephen Verderber, Sami Chadi and Fayez Quereshy in HERD: Health Environments Research & Design Journal

Supplemental Material, sj-docx-3-her-10.1177_19375867231221559 - The Influence of Exposure to Nature on Inpatient Hospital Stays: A Scoping Review Supplemental Material, sj-docx-3-her-10.1177_19375867231221559 for The Influence of Exposure to Nature on Inpatient Hospital Stays: A Scoping Review by Keegan Guidolin, Flora Jung, Sarah Hunter, Han Yan, Marina Englesakis, Stephen Verderber, Sami Chadi and Fayez Quereshy in HERD: Health Environments Research & Design Journal
