# Supplementary material for: The Influence of Exposure to Nature on Inpatient Hospital Stays: A Scoping Review
Source: HERD. 2024 Jan 30;17(2):360–75. doi: 10.1177/19375867231221559 (PMC11080386; doi:10.1177/19375867231221559)
Supplement: Supplemental Material, sj-docx-1-her-10.1177_19375867231221559 - The Influence of Exposure to Nature on Inpatient Hospital Stays: A Scoping Review [file sj-docx-1-her-10.1177_19375867231221559.docx]

**Appendix 1 – Search Strategy**

# Search history record for Scoping Review: Exposure to Nature and Hospitalized Patients

| Review/Search Topic: Exposure to nature and hospitalized patients**;** limited to English language, human subjects, conference and other non-journal literature removed where possible | Searcher: Marina Englesakis |
| --- | --- |
| Investigator(s): Drs F Quereshy, K Guidolin, F Jung, and S Hunter, General Surgery | Date: Wednesday, November 24, 2021 |

| Databases | **Database Dates covered** | **Date Database  was searched** | **# Citations** | **# Duplicate Citations** | **Total Citations remaining** | **Notes/Comments** |
| --- | --- | --- | --- | --- | --- | --- |
| Medline (Ovid) | 1946 – Nov. 23, 2021 | Nov. 24, 2021 | 2226 |  |  |  |
| Medline ePub Ahead of Print / Medline In-Process & Other Non-Indexed Citations (Ovid) | 2021 Nov. 23, 2021 | Nov. 24, 2021 | 63 |  |  |  |
| Embase (Ovid) | 1947 – Nov. 23, 2021 | Nov. 24, 2021 | 3697 |  |  | Non-article (i.e. conference literature) removed at source |
| Cochrane Central Register of Controlled Trials (Ovid) | 1991 – present | Nov. 24, 2021 | 140 |  |  | Non-article (i.e. conference literature) removed at source |
| Cochrane Database of Systematic Reviews (Ovid) | 2005 – present | Nov. 24, 2021 | 3 |  |  |  |
| APA PsycINFO (Ovid) | 1806 – Nov. Week 3, 2021 | Nov. 24, 2021 | 116 |  |  | Non-article (i.e. conference literature) and Medline citations removed at source |
| Ovid Emcare Nursing (Ovid) | 1995 – present | Nov. 24, 2021 | 2508 |  |  | Non-article (i.e. conference literature) and Medline removed at source |
| Web of Science (Clarivate) | 1900 – Nov. 22, 2021 | Nov. 24, 2021 | 2479 |  |  | Medline removed at source |
| Scopus (Elsevier) | 1960 – present | Nov. 24, 2021 | 103 |  |  | Medline/Embase removed at source |
| CINAHL (EbscoHost) | 1982 – present | Nov. 24, 2021 | 876 |  |  | Non-article (i.e. conference literature) and Medline removed at source |
| Avery Index (Avery Index to Architectural Periodicals) (ProQuest) | 1. – present | Nov. 24, 2021 | 140 |  |  | Limited to articles only |
| DAAI – Design & Applied Arts Index (ProQuest) | 1. – present | Nov. 24, 2021 | 628 |  |  | Limited to journal articles only; scholarly |
| JSTOR | 1930 - present | Nov. 24, 2021 | **n/a** |  |  | **NOT USED; Advanced search** is too rudimentary for KS searching. Clients may wish to conduct searches on this database for supplemental information |
|  |  | Totals: | 12979 | 0 | 0 | Results in a compressed EndNote Library |
|  |  |  |  |  |  |  |

#
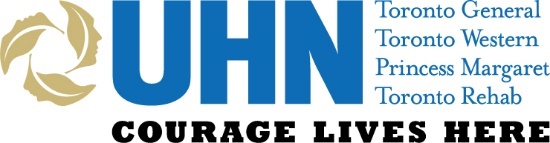
UHN Health Sciences Libraries

# Literature Search Results

For: Drs F Quereshy, K Guidolin, F Jung, and S Hunter

Department: General Surgery

Date Completed: Wed., November 24, 2021

Tel:

Fax:

Email:

**Following are your scoping review searches for**:

**Exposure to nature and hospitalized patients;** limited to English language, human subjects; conference and other non-journal literature removed where possible.

**The databases searched were**:

1. [Medline](#Medline); 2. [Medline In-Process/ePubs](#Medline_In_Process); 3. [Embase](#Embase); 4. [CCTR](#CCTR); 5. [CDSR](#CDSR); 6. [APA PsycINFO;](#APA_PsycINFO) 7. [Ovid Emcare Nursing;](#Ovid_EmCare_Nursing) 8. [CINAHL](#CINAHL); 9. [Scopus;](#Scopus) 10. [Web of Science](#Web_of_Science); 11. [JSTOR](#JSTOR); 12. [Avery Index](#Avery_Index); 13. [DAAI](#DAAI)

**RESULTS & STRATEGY USED**: *see following*

**Search Completed By:** Marina Englesakis, Information Specialist

You may contact me either by telephone at (416) 340-4800 x3022 or via e-mail at [*marina.englesakis@uhn.ca*](mailto:marina.englesakis@uhn.ca)*.*

It is important that you are satisfied with your search results.

If you have any questions regarding this search, or if the results were not satisfactory, please do not hesitate in contacting me.

To request items not available in our library system, an INTERLIBRARY LOAN REQUEST FORM can be obtained from the library’s circulation desk or through the Virtual Library (<http://www.uhn.ca/Education/libraries/ill.asp> ). Any questions regarding our Document Delivery Service can be directed to Caleb by telephone at 416-340-4121 or by email at [Caleb.Nault@uhn.ca](mailto:Caleb.Nault@uhn.ca).

For any other circulation inquiries:

Toronto General Hospital Library: (416) 340-3429

Toronto Western Hospital Library: (416) 603-5750

Toronto Rehab Library: (416) 597-3422, ext. 3050

Princess Margaret Library: (416) 946-4482

Search strategy saved as 2021-11-23 FQ KG Exposure to Nature and Hospitalized Patients

# Medline

Ovid MEDLINE(R) 1946 to November 23, 2021

| **#** | **Searches** | **Results** |
| --- | --- | --- |
| 1 | Environment/ and (physical or build* or built or hospital or hospitals or inpatient? or interior? or facility or facilities or architectur*).mp. | 9237 |
| 2 | "Facility Design and Construction"/ | 9263 |
| 3 | "Hospital Design and Construction"/ | 9955 |
| 4 | "Interior Design and Furnishings"/ | 4667 |
| 5 | "Patient’s Rooms"/ | 3015 |
| 6 | Architecture/ | 2586 |
| 7 | Bed Occupancy/ | 2622 |
| 8 | Birthing Centers/ | 840 |
| 9 | Building Codes/ | 546 |
| 10 | Built Environment/ | 947 |
| 11 | Delivery Rooms/ | 1702 |
| 12 | Environment Design/ | 6899 |
| 13 | Environmental Medicine/ | 471 |
| 14 | Evidence-Based Facility Design/ | 57 |
| 15 | exp Hospitals/ | 295486 |
| 16 | exp Academic Medical Centers/ | 98456 |
| 17 | exp Intensive Care Units/ | 95772 |
| 18 | exp Medicine/ and (architect* or building? or department? or environment* or hospital or hospitals or landscape? or ward? or unit? or floor? or hospitali#ation? or facility or facilities or room or rooms or roomed or rooming).mp. | 216082 |
| 19 | Health Facilities, Proprietary/ | 1268 |
| 20 | Health Facilities/ | 16856 |
| 21 | Health Facility Environment/ | 4343 |
| 22 | Hospital Bed Capacity/ | 4328 |
| 23 | exp Hospital Units/ | 124094 |
| 24 | Hospitals, Proprietary/ | 4364 |
| 25 | Universal Design/ | 44 |
| 26 | Waiting Rooms/ | 31 |
| 27 | architect*.in,hw. | 20312 |
| 28 | (general adj3 (department? or ward? or unit? or floor? or hospitali#ation?)).mp. | 13334 |
| 29 | (healing adj1 (place or places)).mp. | 29 |
| 30 | (healing adj1 (space or spaces)).mp. | 39 |
| 31 | (health* adj1 environment?).ti,ab,jw. | 8032 |
| 32 | ((health care or healthcare) adj1 design???).mp. | 460 |
| 33 | ((health care or healthcare) adj1 setting?).mp. | 14786 |
| 34 | (healthy adj1 (place? or space?)).mp. | 78 |
| 35 | (hospital? adj3 (department? or ward? or unit? or floor? or hospitali#ation?)).mp. | 88220 |
| 36 | (in-patient?? adj3 room?).mp. | 407 |
| 37 | (inpatient?? adj3 room?).mp. | 464 |
| 38 | (patient?? adj3 room?).mp. | 7286 |
| 39 | (public? adj2 area?).mp. | 1582 |
| 40 | (surgical adj3 (department? or ward? or unit? or floor? or hospitali#ation?)).mp. | 17610 |
| 41 | or/1-40 [ Hospitals or Health Care Environment ] | 768712 |
| 42 | Nature/ | 943 |
| 43 | Bays/ | 2585 |
| 44 | Birds/ | 39947 |
| 45 | Estuaries/ | 3648 |
| 46 | Forests/ | 11605 |
| 47 | Gardens/ | 423 |
| 48 | Horticulture/ | 66 |
| 49 | Horticultural Therapy/ | 80 |
| 50 | Lakes/ | 11048 |
| 51 | "Oceans and Seas"/ | 13901 |
| 52 | Plants/ | 81216 |
| 53 | Ponds/ | 1770 |
| 54 | Rivers/ | 30798 |
| 55 | exp Songbirds/ | 10156 |
| 56 | Trees/ | 27935 |
| 57 | Vocalization, Animal/ | 10686 |
| 58 | Wilderness/ | 324 |
| 59 | exp Auditory Perception/ and exp Nature/ | 7 |
| 60 | (access* adj2 natural).mp. | 521 |
| 61 | (access* adj2 nature).mp. | 192 |
| 62 | (acoust* adj3 access* adj3 (nature or natural)).mp. | 0 |
| 63 | (audib* adj3 access* adj3 (nature or natural)).mp. | 0 |
| 64 | (auditor* adj3 access* adj3 (nature or natural)).mp. | 0 |
| 65 | (commun??? adj2 nature).mp. | 173 |
| 66 | (biophili?? adj2 design*).mp. | 9 |
| 67 | (biophili?? adj2 imag*).mp. | 2 |
| 68 | (contact* adj2 nature).mp. | 264 |
| 69 | (experienc* adj2 nature).mp. | 329 |
| 70 | (green adj2 belt*).mp. | 74 |
| 71 | green infrastructur*.mp. | 329 |
| 72 | ((hear or hears or hearing or heard) adj3 (nature or natural)).mp. | 206 |
| 73 | ((hear or hears or hearing or heard) adj3 animal?).mp. | 447 |
| 74 | ((hear or hears or hearing or heard) adj3 bird?).mp. | 91 |
| 75 | ((hear or hears or hearing or heard) adj3 rain*).mp. | 2 |
| 76 | ((hear or hears or hearing or heard) adj3 water).mp. | 43 |
| 77 | ((hear or hears or hearing or heard) adj3 wind?).mp. | 13 |
| 78 | (hear* adj3 access* adj3 (nature or natural)).mp. | 1 |
| 79 | (listen* adj3 (nature or natural)).mp. | 152 |
| 80 | (listen* adj3 animal?).mp. | 25 |
| 81 | (listen* adj3 bird?).mp. | 10 |
| 82 | (listen* adj3 rain*).mp. | 4 |
| 83 | (listen* adj3 water?).mp. | 6 |
| 84 | (listen* adj3 wind?).mp. | 3 |
| 85 | (natural adj2 environ*).mp. | 12337 |
| 86 | (natural adj2 imag*).mp. | 1441 |
| 87 | (natural adj2 land*).mp. | 712 |
| 88 | (natural adj2 light*).mp. | 1480 |
| 89 | (natural adj2 scene*).mp. | 1476 |
| 90 | (natural adj2 setting*).mp. | 1663 |
| 91 | (natural adj2 sound?).mp. | 568 |
| 92 | (natural adj2 spac*).mp. | 280 |
| 93 | (natural adj2 view*).mp. | 541 |
| 94 | (natural adj2 virtual*).mp. | 56 |
| 95 | (natural adj1 world?).mp. | 806 |
| 96 | (nature adj2 environ*).mp. | 559 |
| 97 | (nature adj2 imag*).mp. | 216 |
| 98 | (nature adj2 landscape?).mp. | 38 |
| 99 | (nature adj2 scene*).mp. | 58 |
| 100 | (nature adj2 simulat*).mp. | 67 |
| 101 | (nature?? adj2 sound?).mp. | 91 |
| 102 | (nature adj2 spac??).mp. | 80 |
| 103 | (nature adj2 stimul*).mp. | 289 |
| 104 | nature-theme?.mp. | 4 |
| 105 | (nature adj1 theme?).mp. | 13 |
| 106 | (nature adj2 view*).mp. | 177 |
| 107 | (nature adj2 virtual*).mp. | 51 |
| 108 | nature-based.mp. | 634 |
| 109 | (nature-based adj2 art?).mp. | 3 |
| 110 | ((near or nearby) adj3 (nature or natural)).mp. | 581 |
| 111 | (outdoor? adj2 (art or arts or artwork*)).mp. | 5 |
| 112 | (outdoor? adj2 imag*).mp. | 24 |
| 113 | (outdoor? adj2 photo*).mp. | 122 |
| 114 | (physical* adj3 access* adj3 (nature or natural)).mp. | 3 |
| 115 | (scenic adj2 view*).mp. | 11 |
| 116 | (visual* adj3 access* adj3 (nature or natural)).mp. | 4 |
| 117 | airscape?.mp. | 2 |
| 118 | artwork?.mp. | 792 |
| 119 | balconies.mp. | 55 |
| 120 | balcony.mp. | 105 |
| 121 | biophili???.mp. | 81 |
| 122 | birdsong?.mp. | 640 |
| 123 | "blue space?".mp. | 131 |
| 124 | bluespace?.mp. | 8 |
| 125 | cloudscape?.mp. | 0 |
| 126 | countryside?.mp. | 1375 |
| 127 | courtyard?.mp. | 160 |
| 128 | daylight*.mp. | 3266 |
| 129 | ecotherap*3.mp. | 6 |
| 130 | fauna.mp. | 10160 |
| 131 | "fine art?".mp. | 331 |
| 132 | flower?.mp. | 34219 |
| 133 | flowerbed?.mp. | 12 |
| 134 | (forest? not ("causal forest?" or "forest classifier?" or "forest plot?" or "random* forest?" or "wake forest" or "decision* forest?")).mp. | 48228 |
| 135 | (garden or gardens).mp. | 8883 |
| 136 | (green adj2 space?).mp. | 1518 |
| 137 | greenbelt?.mp. | 69 |
| 138 | greenery.mp. | 196 |
| 139 | greenspace?.mp. | 333 |
| 140 | "green space?".mp. | 1468 |
| 141 | horticultur*.mp. | 2778 |
| 142 | ((landscape or landscapes or landscaping) not (landscape? adj2 (biologic* or regulatory or chang* or cultural* or genetic* or genomic* or global* or practice? or practise? or treatment? or trial? or national or online or economic* or online or techn* or fraught or risk? or contact or test*3 or service? or tele* or policy or politic* or remediat* or health* or institution* or insur* or underemploy* or unemploy*))).mp. | 41751 |
| 143 | (mural or murals).mp. | 6424 |
| 144 | non-city.mp. | 12 |
| 145 | non-urban.mp. | 561 |
| 146 | non-window*.mp. | 9 |
| 147 | nonwindow*.mp. | 5 |
| 148 | "open space?".mp. | 1097 |
| 149 | (outdoor? adj3 (life or panoram* or scene??? or space? or view? or vista?)).mp. | 569 |
| 150 | "outside world".mp. | 970 |
| 151 | (panoramic adj2 (scen* or view? or vista?)).mp. | 547 |
| 152 | parkland?.mp. | 693 |
| 153 | phytotherap*3.mp. | 40637 |
| 154 | (plant? adj1 (living or growing)).mp. | 2896 |
| 155 | "public space?".mp. | 921 |
| 156 | riverscape?.mp. | 70 |
| 157 | (roof or roofs or rooftop?).mp. | 7241 |
| 158 | scenery.mp. | 406 |
| 159 | seascape?.mp. | 283 |
| 160 | (terrace or terraces).mp. | 607 |
| 161 | (vista and (nature or natural)).mp. | 112 |
| 162 | (vistas and (nature or natural)).mp. | 86 |
| 163 | waterscape?.mp. | 29 |
| 164 | wilderness??.mp. | 1357 |
| 165 | (wilds or wilderness).mp. | 1388 |
| 166 | (((window or windows) not ((spss adj2 windows) or (statistics adj2 windows) or (time adj1 window?) or time-window? or "time window")) or (ibm adj4 windows)).mp. | 64644 |
| 167 | ((window or windows) not (hour-window? or minute-window? or (dissection* adj1 window?))).mp. | 74333 |
| 168 | ((window or windows) not (window? adj1 opportunit*)).mp. | 74598 |
| 169 | window-side?.mp. | 12 |
| 170 | windowside?.mp. | 0 |
| 171 | woods.mp. | 2131 |
| 172 | Art/ | 7807 |
| 173 | Paintings/ | 5172 |
| 174 | "Pictorial Works as Topic"/ | 30 |
| 175 | Sculpture/ | 1093 |
| 176 | or/42-175 [ Natural Environment & related terms or how to see them ] | 514944 |
| 177 | 41 and 176 [ Hospitals + Nature ] | 12601 |
| 178 | exp Patients/ | 73162 |
| 179 | exp Hospitalization/ | 269355 |
| 180 | Length of Stay/ | 96908 |
| 181 | Patient-Centered Care/ | 21523 |
| 182 | exp Patient Outcome Assessment/ | 16590 |
| 183 | Patient Readmission/ | 20551 |
| 184 | (patient?? or inpatient??).mp. | 6826449 |
| 185 | ((person?? or client?) adj2 (centered or centred or centric)).mp. | 7679 |
| 186 | (hospital? adj3 (admit* or admis*)).mp. | 91362 |
| 187 | (hospital? adj3 (readmit* or readmis*)).mp. | 9728 |
| 188 | (hospital? adj3 (revisit* or re-visit*)).mp. | 245 |
| 189 | (length adj2 stay*3).mp. | 124370 |
| 190 | hospitali#ed.mp. | 116827 |
| 191 | institutionali#ed.mp. | 11723 |
| 192 | or/178-191 [ Patients & related terms ] | 6918892 |
| 193 | 177 and 192 [ Hospitals + Nature + Patients ] | 3351 |
| 194 | exp Qualitative Research/ | 70038 |
| 195 | exp Communication Barriers/ | 7155 |
| 196 | "Surveys and Questionnaires"/ | 518556 |
| 197 | Evaluation Studies as Topic/ | 122404 |
| 198 | Evaluation Study/ | 260359 |
| 199 | Feasibility Studies/ | 76846 |
| 200 | Grounded Theory/ | 2265 |
| 201 | Health Care Surveys/ | 33711 |
| 202 | Health Impact Assessment/ | 825 |
| 203 | Health Knowledge, Attitudes, Practice/ | 121174 |
| 204 | "Health Services Needs and Demand"/ | 54661 |
| 205 | Hermeneutics/ | 445 |
| 206 | Interview/ | 29164 |
| 207 | Interviews as Topic/ | 66495 |
| 208 | Narration/ | 9352 |
| 209 | Narrative Medicine/ | 165 |
| 210 | Needs Assessment/ | 31907 |
| 211 | Nursing Methodology Research/ | 16405 |
| 212 | Observational Studies as Topic/ | 7144 |
| 213 | Observational Study/ | 114674 |
| 214 | Patient Education as Topic/ | 87634 |
| 215 | Patient Health Questionnaire/ | 728 |
| 216 | Patient Reported Outcome Measures/ | 10171 |
| 217 | Patient Satisfaction/ | 86571 |
| 218 | Personal Narrative/ | 5981 |
| 219 | Personal Satisfaction/ | 21535 |
| 220 | Pilot Projects/ | 135679 |
| 221 | exp Program Evaluation/ | 80535 |
| 222 | ((discourse* or discurs*) adj3 analys#s).mp. | 2352 |
| 223 | ((purpos* adj4 sampl*) or (focus adj group*)).mp. | 63832 |
| 224 | (account or accounts or unstructured or open-ended or open ended or text* or narrative*).mp. | 578685 |
| 225 | (action research or cooperative inquir* or co operative inquir* or co- operative inquir*).mp. | 4157 |
| 226 | (constant adj (comparative or comparison)).mp. | 4620 |
| 227 | (corbin* adj2 strauss*).mp. | 291 |
| 228 | (emic or etic or hermeneutic* or heuristic* or semiotic* or (data adj1 saturat*) or participant observ*).mp. | 20589 |
| 229 | (evaluat* adj2 (study or studies)).mp. | 561842 |
| 230 | (field adj (study or studies or research)).mp. | 15279 |
| 231 | (grounded adj (theor* or study or studies or research or analys#s)).mp. | 11838 |
| 232 | (humanistic or existential or experiential or paradigm*).mp. | 144186 |
| 233 | (life stor* or women* stor*).mp. | 1307 |
| 234 | (life world or life-world or conversation analys#s or personal experience* or theoretical saturation).mp. | 14765 |
| 235 | ((lived or life) adj experience*).mp. | 11791 |
| 236 | (merleau adj ponty*).mp. | 211 |
| 237 | (observational adj (study or studies or research)).mp. | 185618 |
| 238 | (social construct* or (postmodern* or post- structural*) or (post structural* or poststructural*) or post modern* or post-modern* or feminis* or interpret*).mp. | 500435 |
| 239 | (survey? or surveyed or surveying).mp. | 1023799 |
| 240 | (theme* or thematic).mp. | 109928 |
| 241 | (van adj kaam*).mp. | 37 |
| 242 | (van adj manen*).mp. | 414 |
| 243 | biographical method?.mp. | 26 |
| 244 | cluster sampl*.mp. | 6937 |
| 245 | colaizzi*.mp. | 696 |
| 246 | content analys#s.mp. | 29028 |
| 247 | ethnograph*.mp. | 10490 |
| 248 | ethnological research.mp. | 7 |
| 249 | ethnonursing.mp. | 118 |
| 250 | foucault*.mp. | 779 |
| 251 | glaser*.mp. | 815 |
| 252 | heidegger*.mp. | 660 |
| 253 | human science.mp. | 239 |
| 254 | narrative analys#s.mp. | 1304 |
| 255 | narrative review?.mp. | 11551 |
| 256 | observational method*.mp. | 687 |
| 257 | phenomenol*.mp. | 22276 |
| 258 | qualitative.mp. | 240209 |
| 259 | questionnaire*.mp. | 754056 |
| 260 | spiegelberg*.mp. | 71 |
| 261 | theoretical sampl*.mp. | 681 |
| 262 | (client* adj2 (satisfaction or satisfied or satisfy*)).mp. | 1314 |
| 263 | (content analys* or thematic analys* or narrative analys*).mp. | 51938 |
| 264 | (ethnol* or ethnog* or ethnonurs* or emic or etic).mp. | 181853 |
| 265 | (feasib* adj2 (study or studies)).mp. | 83701 |
| 266 | (Grounded adj5 theor*).mp. | 13097 |
| 267 | (hermeneutic* or phenomenolog* or lived experience*).mp. | 27979 |
| 268 | (integrat* adj1 model?).mp. | 5993 |
| 269 | (meta-ethnog* or metaethnog* or meta-narrat* or metanarrat* or meta-interpret* or metainterpret*).mp. | 816 |
| 270 | (metasynthes* or meta-synthes* or metasummar* or meta-summar* or metastud* or meta-stud*).ti,ab. | 1451 |
| 271 | (mixed adj2 method*).mp. | 24699 |
| 272 | (multiple adj1 perspective?).mp. | 1003 |
| 273 | (needs adj2 assessment*).mp. | 36498 |
| 274 | (patient?? adj2 (satisfaction or satisfied or satisfy*)).mp. | 109336 |
| 275 | (person?? adj2 (satisfaction or satisfied or satisfy*)).mp. | 22291 |
| 276 | (personal adj1 (story or stories)).mp. | 517 |
| 277 | (personal adj1 account???).mp. | 971 |
| 278 | (program* adj3 evaluat*).mp. | 83961 |
| 279 | (qualitative adj5 metaanaly*).mp. | 3 |
| 280 | (qualitative adj5 meta-analy*).mp. | 609 |
| 281 | (semantic? adj2 description?).mp. | 96 |
| 282 | (therapeutic adj1 model?).mp. | 855 |
| 283 | (treatment? adj1 model?).mp. | 3704 |
| 284 | (unmet adj2 need?).mp. | 19756 |
| 285 | action research.ti,ab. | 3807 |
| 286 | contextual*.mp. | 38238 |
| 287 | focus group?.mp. | 51119 |
| 288 | frame work*.mp. | 401 |
| 289 | framework*.mp. | 233476 |
| 290 | giorgi*.mp. | 677 |
| 291 | interview?.mp. | 324780 |
| 292 | mixed-method*.mp. | 22974 |
| 293 | multimethod inquir*.mp. | 1 |
| 294 | multi-method inquir*.mp. | 2 |
| 295 | multiperspective?.mp. | 129 |
| 296 | multi-perspective?.mp. | 188 |
| 297 | narrative?.mp. | 47630 |
| 298 | phenomenological*.mp. | 15623 |
| 299 | qualitative*.mp. | 278273 |
| 300 | qualitative.mp. | 240209 |
| 301 | questionnaire?.mp. | 754005 |
| 302 | thematic.mp. | 35385 |
| 303 | theme.mp. | 21686 |
| 304 | themes.mp. | 70340 |
| 305 | or/194-304 [ Qualitative Research or Questionnaires & Related Terms ] | 3999137 |
| 306 | Clinical Trial, Phase III/ | 19453 |
| 307 | exp Clinical Trial/ | 918160 |
| 308 | Clinical Trials, Phase III as Topic/ | 10450 |
| 309 | Comparative Study/ | 1903406 |
| 310 | Controlled Clinical Trial/ | 94531 |
| 311 | Controlled Clinical Trials as Topic/ | 5614 |
| 312 | Cross-Sectional Studies/ | 399587 |
| 313 | Double-Blind Method/ | 168486 |
| 314 | Equivalence Trial/ | 913 |
| 315 | Equivalence Trials as Topic/ | 552 |
| 316 | exp Case-Control Studies/ | 1252758 |
| 317 | exp Cohort Studies/ | 2251326 |
| 318 | exp Randomized Controlled Trial/ | 552101 |
| 319 | exp Randomized Controlled Trials as Topic/ | 154107 |
| 320 | Longitudinal Studies/ | 152601 |
| 321 | Meta-Analysis as Topic/ | 20550 |
| 322 | Meta-Analysis/ | 147013 |
| 323 | Multicenter Studies as Topic/ | 20442 |
| 324 | Multicenter Study/ | 308980 |
| 325 | Observational Study/ | 114674 |
| 326 | Observational Studies as Topic/ | 7144 |
| 327 | Placebos/ | 35782 |
| 328 | Pragmatic Clinical Trial/ | 1973 |
| 329 | Pragmatic Clinical Trials as Topic/ | 708 |
| 330 | Prospective Studies/ | 604176 |
| 331 | Retrospective Studies/ | 967523 |
| 332 | Systematic Review/ | 173465 |
| 333 | Systematic Reviews as Topic/ | 7045 |
| 334 | Validation Study/ | 107756 |
| 335 | Validation Studies as Topic/ | 2371 |
| 336 | ("phase 1" or "phase1" or "phase I").mp. | 64447 |
| 337 | ("phase 2" or "phase2" or "phase II").mp. | 88053 |
| 338 | ("phase 3" or "phase3" or "phase III").mp. | 58536 |
| 339 | ((multicenter* or multicentre*) adj2 (trial? or study or studies)).mp. | 346366 |
| 340 | ((noninferiority or non-inferiority) adj4 (trial? or study or studies)).mp. | 5034 |
| 341 | ((single or double or triple or treble) adj3 (blind* or mask*)).mp. | 237250 |
| 342 | (case control* adj2 (study or studies)).mp. | 337229 |
| 343 | (comparative adj2 (trial? or study or studies)).mp. | 1958773 |
| 344 | (conceal* adj2 allocat*).mp. | 2842 |
| 345 | (controlled adj1 clinical adj2 (trial? or study or studies)).mp. | 128445 |
| 346 | (cross-sectional* adj2 (study or studies)).mp. | 423511 |
| 347 | (equivalen* adj4 (trial? or study or studies)).mp. | 5569 |
| 348 | (evaluation adj1 (study or studies)).mp. | 387554 |
| 349 | (longitudinal* adj2 (study or studies)).mp. | 189653 |
| 350 | (meta-anal* or metanal* or metaanal*).mp. | 212591 |
| 351 | (observational adj2 (trial? or study or studies)).mp. | 196411 |
| 352 | (overview? adj4 (review or reviews)).mp. | 17970 |
| 353 | (pragmatic adj2 (trial? or study or studies)).mp. | 4798 |
| 354 | (prospective* adj2 (study or studies)).mp. | 693637 |
| 355 | (retrospective* adj2 (study or studies)).mp. | 1001993 |
| 356 | (superiority adj4 (trial? or study or studies)).mp. | 3495 |
| 357 | (systematic adj4 (review or reviews or overview or overviews)).mp. | 210979 |
| 358 | (validation adj1 (study or studies)).mp. | 120439 |
| 359 | cohort*.mp. | 710985 |
| 360 | placebo*.mp. | 224169 |
| 361 | qualitativ*.mp. | 278405 |
| 362 | quasirandom*.mp. | 124 |
| 363 | random*.mp. | 1324142 |
| 364 | research.ti,hw,pt. | 9970997 |
| 365 | semiquantitative.mp. | 18934 |
| 366 | or/306-365 [ Studies ] | 13750510 |
| 367 | 305 or 366 [ Qualitative or Quantitative Research ] | 14892719 |
| 368 | 193 and 367 [ Hospitals + Nature + Patients + (Qualitative or Quantitative) ] | 2405 |
| 369 | limit 368 to english language | 2234 |
| 370 | remove duplicates from 369 [ Removal of internal Medline duplicates (if any) ] | 2233 |
| 371 | 370 not (exp animals/ not (exp animals/ and exp humans/)) | 2226 |
| 372 | limit 370 to humans | 2182 |
| 373 | 371 or 372 [ Final MEDLINE result ] | 2226 |

# Medline In-Process

Ovid MEDLINE(R) Epub Ahead of Print and In-Process, In-Data-Review & Other Non-Indexed Citations November 23, 2021

| **#** | **Searches** | **Results** |
| --- | --- | --- |
| 1 | Environment/ and (physical or build* or built or hospital or hospitals or inpatient? or interior? or facility or facilities or architectur*).mp. | 0 |
| 2 | "Facility Design and Construction"/ | 0 |
| 3 | "Hospital Design and Construction"/ | 0 |
| 4 | "Interior Design and Furnishings"/ | 0 |
| 5 | "Patient’s Rooms"/ | 0 |
| 6 | Architecture/ | 0 |
| 7 | Bed Occupancy/ | 0 |
| 8 | Birthing Centers/ | 0 |
| 9 | Building Codes/ | 0 |
| 10 | Built Environment/ | 0 |
| 11 | Delivery Rooms/ | 0 |
| 12 | Environment Design/ | 0 |
| 13 | Environmental Medicine/ | 0 |
| 14 | Evidence-Based Facility Design/ | 0 |
| 15 | exp Hospitals/ | 0 |
| 16 | exp Academic Medical Centers/ | 0 |
| 17 | exp Intensive Care Units/ | 0 |
| 18 | exp Medicine/ and (architect* or building? or department? or environment* or hospital or hospitals or landscape? or ward? or unit? or floor? or hospitali#ation? or facility or facilities or room or rooms or roomed or rooming).mp. | 1 |
| 19 | Health Facilities, Proprietary/ | 0 |
| 20 | Health Facilities/ | 0 |
| 21 | Health Facility Environment/ | 0 |
| 22 | Hospital Bed Capacity/ | 0 |
| 23 | exp Hospital Units/ | 0 |
| 24 | Hospitals, Proprietary/ | 0 |
| 25 | Universal Design/ | 0 |
| 26 | Waiting Rooms/ | 0 |
| 27 | architect*.in,hw. | 9313 |
| 28 | (general adj3 (department? or ward? or unit? or floor? or hospitali#ation?)).mp. | 1873 |
| 29 | (healing adj1 (place or places)).mp. | 2 |
| 30 | (healing adj1 (space or spaces)).mp. | 6 |
| 31 | (health* adj1 environment?).ti,ab,jw. | 869 |
| 32 | ((health care or healthcare) adj1 design???).mp. | 67 |
| 33 | ((health care or healthcare) adj1 setting?).mp. | 3000 |
| 34 | (healthy adj1 (place? or space?)).mp. | 13 |
| 35 | (hospital? adj3 (department? or ward? or unit? or floor? or hospitali#ation?)).mp. | 6661 |
| 36 | (in-patient?? adj3 room?).mp. | 71 |
| 37 | (inpatient?? adj3 room?).mp. | 93 |
| 38 | (patient?? adj3 room?).mp. | 688 |
| 39 | (public? adj2 area?).mp. | 279 |
| 40 | (surgical adj3 (department? or ward? or unit? or floor? or hospitali#ation?)).mp. | 2270 |
| 41 | or/1-40 [ Hospitals or Health Care Environment ] | 24232 |
| 42 | Nature/ | 0 |
| 43 | Bays/ | 0 |
| 44 | Birds/ | 0 |
| 45 | Estuaries/ | 0 |
| 46 | Forests/ | 0 |
| 47 | Gardens/ | 0 |
| 48 | Horticulture/ | 0 |
| 49 | Horticultural Therapy/ | 0 |
| 50 | Lakes/ | 0 |
| 51 | "Oceans and Seas"/ | 0 |
| 52 | Plants/ | 0 |
| 53 | Ponds/ | 0 |
| 54 | Rivers/ | 0 |
| 55 | exp Songbirds/ | 0 |
| 56 | Trees/ | 0 |
| 57 | Vocalization, Animal/ | 0 |
| 58 | Wilderness/ | 0 |
| 59 | exp Auditory Perception/ and exp Nature/ | 0 |
| 60 | (access* adj2 natural).mp. | 149 |
| 61 | (access* adj2 nature).mp. | 50 |
| 62 | (acoust* adj3 access* adj3 (nature or natural)).mp. | 0 |
| 63 | (audib* adj3 access* adj3 (nature or natural)).mp. | 0 |
| 64 | (auditor* adj3 access* adj3 (nature or natural)).mp. | 0 |
| 65 | (commun??? adj2 nature).mp. | 41 |
| 66 | (biophili?? adj2 design*).mp. | 6 |
| 67 | (biophili?? adj2 imag*).mp. | 0 |
| 68 | (contact* adj2 nature).mp. | 94 |
| 69 | (experienc* adj2 nature).mp. | 103 |
| 70 | (green adj2 belt*).mp. | 17 |
| 71 | green infrastructur*.mp. | 174 |
| 72 | ((hear or hears or hearing or heard) adj3 (nature or natural)).mp. | 34 |
| 73 | ((hear or hears or hearing or heard) adj3 animal?).mp. | 48 |
| 74 | ((hear or hears or hearing or heard) adj3 bird?).mp. | 14 |
| 75 | ((hear or hears or hearing or heard) adj3 rain*).mp. | 0 |
| 76 | ((hear or hears or hearing or heard) adj3 water).mp. | 4 |
| 77 | ((hear or hears or hearing or heard) adj3 wind?).mp. | 4 |
| 78 | (hear* adj3 access* adj3 (nature or natural)).mp. | 0 |
| 79 | (listen* adj3 (nature or natural)).mp. | 15 |
| 80 | (listen* adj3 animal?).mp. | 5 |
| 81 | (listen* adj3 bird?).mp. | 0 |
| 82 | (listen* adj3 rain*).mp. | 0 |
| 83 | (listen* adj3 water?).mp. | 3 |
| 84 | (listen* adj3 wind?).mp. | 0 |
| 85 | (natural adj2 environ*).mp. | 2962 |
| 86 | (natural adj2 imag*).mp. | 729 |
| 87 | (natural adj2 land*).mp. | 201 |
| 88 | (natural adj2 light*).mp. | 572 |
| 89 | (natural adj2 scene*).mp. | 376 |
| 90 | (natural adj2 setting*).mp. | 352 |
| 91 | (natural adj2 sound?).mp. | 100 |
| 92 | (natural adj2 spac*).mp. | 85 |
| 93 | (natural adj2 view*).mp. | 101 |
| 94 | (natural adj2 virtual*).mp. | 18 |
| 95 | (natural adj1 world?).mp. | 283 |
| 96 | (nature adj2 environ*).mp. | 217 |
| 97 | (nature adj2 imag*).mp. | 87 |
| 98 | (nature adj2 landscape?).mp. | 8 |
| 99 | (nature adj2 scene*).mp. | 27 |
| 100 | (nature adj2 simulat*).mp. | 34 |
| 101 | (nature?? adj2 sound?).mp. | 30 |
| 102 | (nature adj2 spac??).mp. | 52 |
| 103 | (nature adj2 stimul*).mp. | 63 |
| 104 | (nature adj2 view*).mp. | 39 |
| 105 | (nature adj2 virtual*).mp. | 18 |
| 106 | nature-based.mp. | 277 |
| 107 | (nature-based adj2 art?).mp. | 0 |
| 108 | ((near or nearby) adj3 (nature or natural)).mp. | 210 |
| 109 | (outdoor? adj2 (art or arts or artwork*)).mp. | 3 |
| 110 | (outdoor? adj2 imag*).mp. | 47 |
| 111 | (outdoor? adj2 photo*).mp. | 37 |
| 112 | (physical* adj3 access* adj3 (nature or natural)).mp. | 1 |
| 113 | (scenic adj2 view*).mp. | 1 |
| 114 | (visual* adj3 access* adj3 (nature or natural)).mp. | 1 |
| 115 | airscape?.mp. | 0 |
| 116 | artwork?.mp. | 614 |
| 117 | balconies.mp. | 31 |
| 118 | balcony.mp. | 46 |
| 119 | biophili???.mp. | 32 |
| 120 | birdsong?.mp. | 115 |
| 121 | "blue space?".mp. | 33 |
| 122 | bluespace?.mp. | 5 |
| 123 | cloudscape?.mp. | 0 |
| 124 | countryside?.mp. | 179 |
| 125 | courtyard?.mp. | 31 |
| 126 | daylight*.mp. | 958 |
| 127 | ecotherap*3.mp. | 4 |
| 128 | fauna.mp. | 2550 |
| 129 | "fine art?".mp. | 79 |
| 130 | flower?.mp. | 9722 |
| 131 | flowerbed?.mp. | 2 |
| 132 | (forest? not ("causal forest?" or "forest classifier?" or "forest plot?" or "random* forest?" or "wake forest" or "decision* forest?")).mp. | 13655 |
| 133 | (garden or gardens).mp. | 2791 |
| 134 | (green adj2 space?).mp. | 356 |
| 135 | greenbelt?.mp. | 19 |
| 136 | greenery.mp. | 50 |
| 137 | greenspace?.mp. | 112 |
| 138 | "green space?".mp. | 341 |
| 139 | horticultur*.mp. | 1154 |
| 140 | ((landscape or landscapes or landscaping) not (landscape? adj2 (biologic* or regulatory or chang* or cultural* or genetic* or genomic* or global* or practice? or practise? or treatment? or trial? or national or online or economic* or online or techn* or fraught or risk? or contact or test*3 or service? or tele* or policy or politic* or remediat* or health* or institution* or insur* or underemploy* or unemploy*))).mp. | 15048 |
| 141 | (mural or murals).mp. | 859 |
| 142 | non-city.mp. | 2 |
| 143 | non-urban.mp. | 131 |
| 144 | non-window*.mp. | 1 |
| 145 | nonwindow*.mp. | 3 |
| 146 | "open space?".mp. | 314 |
| 147 | (outdoor? adj3 (life or panoram* or scene??? or space? or view? or vista?)).mp. | 231 |
| 148 | "outside world".mp. | 177 |
| 149 | (panoramic adj2 (scen* or view? or vista?)).mp. | 167 |
| 150 | parkland?.mp. | 94 |
| 151 | phytotherap*3.mp. | 368 |
| 152 | (plant? adj1 (living or growing)).mp. | 1151 |
| 153 | "public space?".mp. | 277 |
| 154 | riverscape?.mp. | 18 |
| 155 | (roof or roofs or rooftop?).mp. | 1443 |
| 156 | scenery.mp. | 120 |
| 157 | seascape?.mp. | 80 |
| 158 | (terrace or terraces).mp. | 1076 |
| 159 | (vista and (nature or natural)).mp. | 18 |
| 160 | (vistas and (nature or natural)).mp. | 25 |
| 161 | waterscape?.mp. | 9 |
| 162 | wilderness??.mp. | 196 |
| 163 | (wilds or wilderness).mp. | 208 |
| 164 | (((window or windows) not ((spss adj2 windows) or (statistics adj2 windows) or (time adj1 window?) or time-window? or "time window")) or (ibm adj4 windows)).mp. | 19305 |
| 165 | ((window or windows) not (hour-window? or minute-window? or (dissection* adj1 window?))).mp. | 21677 |
| 166 | ((window or windows) not (window? adj1 opportunit*)).mp. | 21722 |
| 167 | window-side?.mp. | 4 |
| 168 | windowside?.mp. | 0 |
| 169 | woods.mp. | 628 |
| 170 | Art/ | 0 |
| 171 | Paintings/ | 0 |
| 172 | "Pictorial Works as Topic"/ | 0 |
| 173 | Sculpture/ | 0 |
| 174 | or/42-173 [ Natural Environment & related terms or how to see them ] | 79413 |
| 175 | 41 and 174 [ Hospitals + Nature ] | 927 |
| 176 | exp Patients/ | 0 |
| 177 | exp Hospitalization/ | 0 |
| 178 | Length of Stay/ | 0 |
| 179 | Patient-Centered Care/ | 0 |
| 180 | exp Patient Outcome Assessment/ | 0 |
| 181 | Patient Readmission/ | 0 |
| 182 | (patient?? or inpatient??).mp. | 908705 |
| 183 | ((person?? or client?) adj2 (centered or centred or centric)).mp. | 1764 |
| 184 | (hospital? adj3 (admit* or admis*)).mp. | 13929 |
| 185 | (hospital? adj3 (readmit* or readmis*)).mp. | 1650 |
| 186 | (hospital? adj3 (revisit* or re-visit*)).mp. | 45 |
| 187 | (length adj2 stay*3).mp. | 10182 |
| 188 | hospitali#ed.mp. | 16975 |
| 189 | institutionali#ed.mp. | 1010 |
| 190 | or/176-189 [ Patients & related terms ] | 915354 |
| 191 | 175 and 190 [ Hospitals + Nature + Patients ] | 107 |
| 192 | exp Qualitative Research/ | 0 |
| 193 | exp Communication Barriers/ | 0 |
| 194 | "Surveys and Questionnaires"/ | 0 |
| 195 | Evaluation Studies as Topic/ | 0 |
| 196 | Evaluation Studies/ | 28 |
| 197 | Feasibility Studies/ | 0 |
| 198 | Grounded Theory/ | 0 |
| 199 | Health Care Surveys/ | 0 |
| 200 | Health Impact Assessment/ | 0 |
| 201 | Health Knowledge, Attitudes, Practice/ | 0 |
| 202 | "Health Services Needs and Demand"/ | 0 |
| 203 | Hermeneutics/ | 0 |
| 204 | Interview/ | 833 |
| 205 | Interviews as Topic/ | 0 |
| 206 | Narration/ | 0 |
| 207 | Narrative Medicine/ | 0 |
| 208 | Needs Assessment/ | 0 |
| 209 | Nursing Methodology Research/ | 0 |
| 210 | Observational Studies as Topic/ | 0 |
| 211 | Observational Study/ | 92 |
| 212 | Patient Education as Topic/ | 0 |
| 213 | Patient Health Questionnaire/ | 0 |
| 214 | Patient Reported Outcome Measures/ | 0 |
| 215 | Patient Satisfaction/ | 0 |
| 216 | Personal Narrative/ | 5 |
| 217 | Personal Satisfaction/ | 0 |
| 218 | Pilot Projects/ | 0 |
| 219 | exp Program Evaluation/ | 0 |
| 220 | ((discourse* or discurs*) adj3 analys#s).mp. | 422 |
| 221 | ((purpos* adj4 sampl*) or (focus adj group*)).mp. | 12316 |
| 222 | (account or accounts or unstructured or open-ended or open ended or text* or narrative*).mp. | 145939 |
| 223 | (action research or cooperative inquir* or co operative inquir* or co- operative inquir*).mp. | 763 |
| 224 | (constant adj (comparative or comparison)).mp. | 680 |
| 225 | (corbin* adj2 strauss*).mp. | 96 |
| 226 | (emic or etic or hermeneutic* or heuristic* or semiotic* or (data adj1 saturat*) or participant observ*).mp. | 5318 |
| 227 | (evaluat* adj2 (study or studies)).mp. | 32886 |
| 228 | (field adj (study or studies or research)).mp. | 2881 |
| 229 | (grounded adj (theor* or study or studies or research or analys#s)).mp. | 1718 |
| 230 | (humanistic or existential or experiential or paradigm*).mp. | 28457 |
| 231 | (life stor* or women* stor*).mp. | 238 |
| 232 | (life world or life-world or conversation analys#s or personal experience* or theoretical saturation).mp. | 1675 |
| 233 | ((lived or life) adj experience*).mp. | 2756 |
| 234 | (merleau adj ponty*).mp. | 45 |
| 235 | (observational adj (study or studies or research)).mp. | 23996 |
| 236 | (social construct* or (postmodern* or post- structural*) or (post structural* or poststructural*) or post modern* or post-modern* or feminis* or interpret*).mp. | 73276 |
| 237 | (survey? or surveyed or surveying).mp. | 105135 |
| 238 | (theme* or thematic).mp. | 24766 |
| 239 | (van adj kaam*).mp. | 4 |
| 240 | (van adj manen*).mp. | 66 |
| 241 | biographical method?.mp. | 5 |
| 242 | cluster sampl*.mp. | 1436 |
| 243 | colaizzi*.mp. | 168 |
| 244 | content analys#s.mp. | 6765 |
| 245 | ethnograph*.mp. | 1849 |
| 246 | ethnological research.mp. | 0 |
| 247 | ethnonursing.mp. | 6 |
| 248 | foucault*.mp. | 164 |
| 249 | glaser*.mp. | 253 |
| 250 | heidegger*.mp. | 61 |
| 251 | human science.mp. | 21 |
| 252 | narrative analys#s.mp. | 265 |
| 253 | narrative review?.mp. | 5792 |
| 254 | observational method*.mp. | 179 |
| 255 | phenomenol*.mp. | 7537 |
| 256 | qualitative.mp. | 48564 |
| 257 | questionnaire*.mp. | 87252 |
| 258 | spiegelberg*.mp. | 9 |
| 259 | theoretical sampl*.mp. | 114 |
| 260 | (client* adj2 (satisfaction or satisfied or satisfy*)).mp. | 226 |
| 261 | (content analys* or thematic analys* or narrative analys*).mp. | 12956 |
| 262 | (ethnol* or ethnog* or ethnonurs* or emic or etic).mp. | 2168 |
| 263 | (feasib* adj2 (study or studies)).mp. | 4644 |
| 264 | (Grounded adj5 theor*).mp. | 2098 |
| 265 | (hermeneutic* or phenomenolog* or lived experience*).mp. | 8976 |
| 266 | (integrat* adj1 model?).mp. | 1383 |
| 267 | (meta-ethnog* or metaethnog* or meta-narrat* or metanarrat* or meta-interpret* or metainterpret*).mp. | 144 |
| 268 | (metasynthes* or meta-synthes* or metasummar* or meta-summar* or metastud* or meta-stud*).ti,ab. | 334 |
| 269 | (mixed adj2 method*).mp. | 7479 |
| 270 | (multiple adj1 perspective?).mp. | 241 |
| 271 | (needs adj2 assessment*).mp. | 1420 |
| 272 | (patient?? adj2 (satisfaction or satisfied or satisfy*)).mp. | 9216 |
| 273 | (person?? adj2 (satisfaction or satisfied or satisfy*)).mp. | 230 |
| 274 | (personal adj1 (story or stories)).mp. | 104 |
| 275 | (personal adj1 account???).mp. | 304 |
| 276 | (program* adj3 evaluat*).mp. | 3338 |
| 277 | (qualitative adj5 metaanaly*).mp. | 0 |
| 278 | (qualitative adj5 meta-analy*).mp. | 140 |
| 279 | (semantic? adj2 description?).mp. | 31 |
| 280 | (therapeutic adj1 model?).mp. | 144 |
| 281 | (treatment? adj1 model?).mp. | 602 |
| 282 | (unmet adj2 need?).mp. | 4972 |
| 283 | action research.ti,ab. | 698 |
| 284 | contextual*.mp. | 8554 |
| 285 | focus group?.mp. | 8858 |
| 286 | frame work*.mp. | 124 |
| 287 | framework*.mp. | 95773 |
| 288 | giorgi*.mp. | 117 |
| 289 | interview?.mp. | 43486 |
| 290 | mixed-method*.mp. | 7033 |
| 291 | multimethod inquir*.mp. | 0 |
| 292 | multi-method inquir*.mp. | 0 |
| 293 | multiperspective?.mp. | 37 |
| 294 | multi-perspective?.mp. | 51 |
| 295 | narrative?.mp. | 13033 |
| 296 | phenomenological*.mp. | 5636 |
| 297 | qualitative*.mp. | 58475 |
| 298 | qualitative.mp. | 48564 |
| 299 | questionnaire?.mp. | 87221 |
| 300 | thematic.mp. | 8902 |
| 301 | theme.mp. | 4881 |
| 302 | themes.mp. | 15710 |
| 303 | or/192-302 [ Qualitative Research or Questionnaires & Related Terms ] | 615742 |
| 304 | Clinical Trial, Phase III/ | 1 |
| 305 | exp Clinical Trial/ | 603 |
| 306 | Clinical Trials, Phase III as Topic/ | 0 |
| 307 | Comparative Study/ | 46 |
| 308 | Controlled Clinical Trial/ | 21 |
| 309 | Controlled Clinical Trials as Topic/ | 0 |
| 310 | Cross-Sectional Studies/ | 0 |
| 311 | Double-Blind Method/ | 0 |
| 312 | Equivalence Trial/ | 0 |
| 313 | Equivalence Trials as Topic/ | 0 |
| 314 | exp Case-Control Studies/ | 1 |
| 315 | exp Cohort Studies/ | 1 |
| 316 | exp Randomized Controlled Trial/ | 277 |
| 317 | exp Randomized Controlled Trials as Topic/ | 0 |
| 318 | Longitudinal Studies/ | 0 |
| 319 | Meta-Analysis as Topic/ | 0 |
| 320 | Meta-Analysis/ | 159 |
| 321 | Multicenter Studies as Topic/ | 0 |
| 322 | Multicenter Study/ | 5 |
| 323 | Observational Study/ | 92 |
| 324 | Observational Studies as Topic/ | 0 |
| 325 | Placebos/ | 0 |
| 326 | Pragmatic Clinical Trial/ | 0 |
| 327 | Pragmatic Clinical Trials as Topic/ | 0 |
| 328 | Prospective Studies/ | 0 |
| 329 | Retrospective Studies/ | 1 |
| 330 | Systematic Review/ | 3284 |
| 331 | Systematic Reviews as Topic/ | 0 |
| 332 | Validation Studies/ | 1 |
| 333 | ("phase 1" or "phase1" or "phase I").mp. | 7056 |
| 334 | ("phase 2" or "phase2" or "phase II").mp. | 8610 |
| 335 | ("phase 3" or "phase3" or "phase III").mp. | 7458 |
| 336 | ((multicenter* or multicentre*) adj2 (trial? or study or studies)).mp. | 9882 |
| 337 | ((noninferiority or non-inferiority) adj4 (trial? or study or studies)).mp. | 802 |
| 338 | ((single or double or triple or treble) adj3 (blind* or mask*)).mp. | 17221 |
| 339 | (case control* adj2 (study or studies)).mp. | 15712 |
| 340 | (comparative adj2 (trial? or study or studies)).mp. | 18551 |
| 341 | (conceal* adj2 allocat*).mp. | 270 |
| 342 | (controlled adj1 clinical adj2 (trial? or study or studies)).mp. | 4487 |
| 343 | (cross-sectional* adj2 (study or studies)).mp. | 50067 |
| 344 | (equivalen* adj4 (trial? or study or studies)).mp. | 632 |
| 345 | (evaluation adj1 (study or studies)).mp. | 1163 |
| 346 | (longitudinal* adj2 (study or studies)).mp. | 13812 |
| 347 | (meta-anal* or metanal* or metaanal*).mp. | 39508 |
| 348 | (observational adj2 (trial? or study or studies)).mp. | 28472 |
| 349 | (overview? adj4 (review or reviews)).mp. | 5597 |
| 350 | (pragmatic adj2 (trial? or study or studies)).mp. | 571 |
| 351 | (prospective* adj2 (study or studies)).mp. | 41274 |
| 352 | (retrospective* adj2 (study or studies)).mp. | 52192 |
| 353 | (superiority adj4 (trial? or study or studies)).mp. | 602 |
| 354 | (systematic adj4 (review or reviews or overview or overviews)).mp. | 49211 |
| 355 | (validation adj1 (study or studies)).mp. | 3244 |
| 356 | cohort*.mp. | 105849 |
| 357 | placebo*.mp. | 20919 |
| 358 | qualitativ*.mp. | 58508 |
| 359 | quasirandom*.mp. | 69 |
| 360 | random*.mp. | 190213 |
| 361 | research.ti,hw,pt. | 282134 |
| 362 | semiquantitative.mp. | 1468 |
| 363 | or/304-362 [ Studies ] | 800145 |
| 364 | 303 or 363 [ Qualitative or Quantitative Research ] | 1193998 |
| 365 | 191 and 364 [ Hospitals + Nature + Patients + (Qualitative or Quantitative) ] | 64 |
| 366 | limit 365 to english language | 63 |
| 367 | remove duplicates from 366 [ Removal of internal Medline ePubs duplicates (if any) ] | 63 |

# Embase

Embase Classic+Embase 1947 to 2021 November 23

| **#** | **Searches** | **Results** |
| --- | --- | --- |
| 1 | Environment/ and (physical or build* or built or hospital or hospitals or inpatient? or interior? or facility or facilities or architectur*).mp. | 24151 |
| 2 | "Facility Design and Construction"/ | 8026 |
| 3 | "Hospital Design and Construction"/ | 9812 |
| 4 | "Interior Design and Furnishings"/ | 5023 |
| 5 | "Patient’s Rooms"/ | 79512 |
| 6 | architect/ | 1150 |
| 7 | Architecture/ | 19628 |
| 8 | Bed Occupancy/ | 3999 |
| 9 | Birthing Centers/ | 4469 |
| 10 | Building Codes/ | 8560 |
| 11 | Built Environment/ | 1091 |
| 12 | Delivery Rooms/ | 3602 |
| 13 | Environment Design/ | 10183 |
| 14 | Environmental Medicine/ | 132 |
| 15 | Evidence-Based Facility Design/ | 9812 |
| 16 | exp Hospitals/ | 1343076 |
| 17 | exp Academic Medical Centers/ | 145592 |
| 18 | exp Intensive Care Units/ | 235866 |
| 19 | exp Medicine/ and (architect* or building? or department? or environment* or hospital or hospitals or landscape? or ward? or unit? or floor? or hospitali#ation? or facility or facilities or room or rooms or roomed or rooming).mp. | 770802 |
| 20 | Health Facilities/ | 72723 |
| 21 | Hospital Bed Capacity/ | 19740 |
| 22 | exp Hospital Units/ | 655685 |
| 23 | Hospitals, Proprietary/ | 446240 |
| 24 | Universal Design/ | 235 |
| 25 | Waiting Rooms/ | 2355 |
| 26 | architect*.in,hw. | 58448 |
| 27 | (general adj3 (department? or ward? or unit? or floor? or hospitali#ation?)).mp. | 24522 |
| 28 | (healing adj1 (place or places)).mp. | 38 |
| 29 | (healing adj1 (space or spaces)).mp. | 45 |
| 30 | (health* adj1 environment?).ti,ab,jw. | 5961 |
| 31 | ((health care or healthcare) adj1 design???).mp. | 938 |
| 32 | ((health care or healthcare) adj1 setting?).mp. | 22363 |
| 33 | (healthy adj1 (place? or space?)).mp. | 118 |
| 34 | (hospital? adj3 (department? or ward? or unit? or floor? or hospitali#ation?)).mp. | 105076 |
| 35 | (in-patient?? adj3 room?).mp. | 776 |
| 36 | (inpatient?? adj3 room?).mp. | 1163 |
| 37 | (patient?? adj3 room?).mp. | 9541 |
| 38 | (public? adj2 area?).mp. | 2340 |
| 39 | (surgical adj3 (department? or ward? or unit? or floor? or hospitali#ation?)).mp. | 33090 |
| 40 | (medical adj3 (department? or ward? or unit? or floor? or hospitali#ation?)).ti,ab. | 45685 |
| 41 | or/1-40 [ Hospitals or Health Care Environment ] | 2062941 |
| 42 | Nature/ | 4142 |
| 43 | Bays/ | 2240 |
| 44 | Birds/ | 52102 |
| 45 | Estuaries/ | 9328 |
| 46 | Forests/ | 21578 |
| 47 | Gardens/ | 15211 |
| 48 | Horticulture/ | 1081 |
| 49 | Horticultural Therapy/ | 137 |
| 50 | Lakes/ | 16496 |
| 51 | "Oceans and Seas"/ | 20522 |
| 52 | Plants/ | 135969 |
| 53 | Ponds/ | 5335 |
| 54 | Rivers/ | 31632 |
| 55 | exp Songbirds/ | 14664 |
| 56 | Trees/ | 32311 |
| 57 | Vocalization, Animal/ | 12285 |
| 58 | Wilderness/ | 492 |
| 59 | exp Auditory Perception/ and exp Nature/ | 16647 |
| 60 | (access* adj2 natural).mp. | 718 |
| 61 | (access* adj2 nature).mp. | 270 |
| 62 | (acoust* adj3 access* adj3 (nature or natural)).mp. | 0 |
| 63 | (audib* adj3 access* adj3 (nature or natural)).mp. | 0 |
| 64 | (auditor* adj3 access* adj3 (nature or natural)).mp. | 1 |
| 65 | (commun??? adj2 nature).mp. | 276 |
| 66 | (biophili?? adj2 design*).mp. | 11 |
| 67 | (biophili?? adj2 imag*).mp. | 2 |
| 68 | (contact* adj2 nature).mp. | 431 |
| 69 | (experienc* adj2 nature).mp. | 458 |
| 70 | (green adj2 belt*).mp. | 137 |
| 71 | green infrastructur*.mp. | 500 |
| 72 | ((hear or hears or hearing or heard) adj3 (nature or natural)).mp. | 301 |
| 73 | ((hear or hears or hearing or heard) adj3 animal?).mp. | 576 |
| 74 | ((hear or hears or hearing or heard) adj3 bird?).mp. | 110 |
| 75 | ((hear or hears or hearing or heard) adj3 rain*).mp. | 5 |
| 76 | ((hear or hears or hearing or heard) adj3 water).mp. | 56 |
| 77 | ((hear or hears or hearing or heard) adj3 wind?).mp. | 20 |
| 78 | (hear* adj3 access* adj3 (nature or natural)).mp. | 1 |
| 79 | (listen* adj3 (nature or natural)).mp. | 183 |
| 80 | (listen* adj3 animal?).mp. | 44 |
| 81 | (listen* adj3 bird?).mp. | 11 |
| 82 | (listen* adj3 rain*).mp. | 4 |
| 83 | (listen* adj3 water?).mp. | 12 |
| 84 | (listen* adj3 wind?).mp. | 3 |
| 85 | (natural adj2 environ*).mp. | 17026 |
| 86 | (natural adj2 imag*).mp. | 2260 |
| 87 | (natural adj2 land*).mp. | 966 |
| 88 | (natural adj2 light*).mp. | 2169 |
| 89 | (natural adj2 scene*).mp. | 2048 |
| 90 | (natural adj2 setting*).mp. | 2230 |
| 91 | (natural adj2 sound?).mp. | 726 |
| 92 | (natural adj2 spac*).mp. | 378 |
| 93 | (natural adj2 view*).mp. | 711 |
| 94 | (natural adj2 virtual*).mp. | 84 |
| 95 | (natural adj1 world?).mp. | 1136 |
| 96 | (nature adj2 environ*).mp. | 966 |
| 97 | (nature adj2 imag*).mp. | 371 |
| 98 | (nature adj2 landscape?).mp. | 69 |
| 99 | (nature adj2 scene*).mp. | 88 |
| 100 | (nature adj2 simulat*).mp. | 110 |
| 101 | (nature?? adj2 sound?).mp. | 147 |
| 102 | (nature adj2 spac??).mp. | 174 |
| 103 | (nature adj2 stimul*).mp. | 436 |
| 104 | nature-theme?.mp. | 7 |
| 105 | (nature adj1 theme?).mp. | 23 |
| 106 | (nature adj2 view*).mp. | 262 |
| 107 | (nature adj2 virtual*).mp. | 81 |
| 108 | nature-based.mp. | 1027 |
| 109 | (nature-based adj2 art?).mp. | 3 |
| 110 | ((near or nearby) adj3 (nature or natural)).mp. | 864 |
| 111 | (outdoor? adj2 (art or arts or artwork*)).mp. | 9 |
| 112 | (outdoor? adj2 imag*).mp. | 67 |
| 113 | (outdoor? adj2 photo*).mp. | 178 |
| 114 | (physical* adj3 access* adj3 (nature or natural)).mp. | 4 |
| 115 | (scenic adj2 view*).mp. | 15 |
| 116 | (visual* adj3 access* adj3 (nature or natural)).mp. | 6 |
| 117 | airscape?.mp. | 2 |
| 118 | artwork?.mp. | 1537 |
| 119 | balconies.mp. | 108 |
| 120 | balcony.mp. | 203 |
| 121 | biophili???.mp. | 112 |
| 122 | birdsong?.mp. | 940 |
| 123 | "blue space?".mp. | 176 |
| 124 | bluespace?.mp. | 12 |
| 125 | cloudscape?.mp. | 0 |
| 126 | countryside?.mp. | 2121 |
| 127 | courtyard?.mp. | 268 |
| 128 | daylight*.mp. | 5328 |
| 129 | ecotherap*3.mp. | 14 |
| 130 | fauna.mp. | 15966 |
| 131 | "fine art?".mp. | 475 |
| 132 | flower?.mp. | 47068 |
| 133 | flowerbed?.mp. | 18 |
| 134 | (forest? not ("causal forest?" or "forest classifier?" or "forest plot?" or "random* forest?" or "wake forest" or "decision* forest?")).mp. | 72351 |
| 135 | (garden or gardens).mp. | 13045 |
| 136 | (green adj2 space?).mp. | 2002 |
| 137 | greenbelt?.mp. | 95 |
| 138 | greenery.mp. | 294 |
| 139 | greenspace?.mp. | 450 |
| 140 | "green space?".mp. | 1921 |
| 141 | horticultur*.mp. | 4391 |
| 142 | ((landscape or landscapes or landscaping) not (landscape? adj2 (biologic* or regulatory or chang* or cultural* or genetic* or genomic* or global* or practice? or practise? or treatment? or trial? or national or online or economic* or online or techn* or fraught or risk? or contact or test*3 or service? or tele* or policy or politic* or remediat* or health* or institution* or insur* or underemploy* or unemploy*))).mp. | 65233 |
| 143 | (mural or murals).mp. | 11355 |
| 144 | non-city.mp. | 24 |
| 145 | non-urban.mp. | 994 |
| 146 | non-window*.mp. | 16 |
| 147 | nonwindow*.mp. | 9 |
| 148 | "open space?".mp. | 1668 |
| 149 | (outdoor? adj3 (life or panoram* or scene??? or space? or view? or vista?)).mp. | 894 |
| 150 | "outside world".mp. | 1646 |
| 151 | (panoramic adj2 (scen* or view? or vista?)).mp. | 913 |
| 152 | parkland?.mp. | 1179 |
| 153 | phytotherap*3.mp. | 20132 |
| 154 | (plant? adj1 (living or growing)).mp. | 4042 |
| 155 | "public space?".mp. | 1375 |
| 156 | riverscape?.mp. | 76 |
| 157 | (roof or roofs or rooftop?).mp. | 13231 |
| 158 | scenery.mp. | 678 |
| 159 | seascape?.mp. | 323 |
| 160 | (terrace or terraces).mp. | 1267 |
| 161 | (vista and (nature or natural)).mp. | 204 |
| 162 | (vistas and (nature or natural)).mp. | 133 |
| 163 | waterscape?.mp. | 35 |
| 164 | wilderness??.mp. | 1941 |
| 165 | (wilds or wilderness).mp. | 1994 |
| 166 | ((window or windows) not ((spss adj3 windows) or (statistics adj2 windows) or (time adj1 window?) or time-window? or "time window" or (ibm adj3 windows))).mp. | 111582 |
| 167 | ((window or windows) not (hour-window? or minute-window? or (dissection* adj1 window?))).mp. | 130020 |
| 168 | ((window or windows) not (window? adj1 opportunit*)).mp. | 130710 |
| 169 | window-side?.mp. | 19 |
| 170 | windowside?.mp. | 0 |
| 171 | woods.mp. | 4044 |
| 172 | Art/ | 54738 |
| 173 | Paintings/ | 9190 |
| 174 | or/42-173 [ Natural Environment & related terms or how to see them ] | 821749 |
| 175 | 41 and 174 [ Hospitals + Nature ] | 42613 |
| 176 | exp Patients/ | 3115090 |
| 177 | exp Hospitalization/ | 443347 |
| 178 | Length of Stay/ | 222451 |
| 179 | Patient-Centered Care/ | 210581 |
| 180 | exp Patient Outcome Assessment/ | 633177 |
| 181 | Patient Readmission/ | 67611 |
| 182 | (patient?? or inpatient??).mp. | 11947604 |
| 183 | ((person?? or client?) adj2 (centered or centred or centric)).mp. | 11302 |
| 184 | (hospital? adj3 (admit* or admis*)).mp. | 336125 |
| 185 | (hospital? adj3 (readmit* or readmis*)).mp. | 82399 |
| 186 | (hospital? adj3 (revisit* or re-visit*)).mp. | 409 |
| 187 | (length adj2 stay*3).mp. | 240512 |
| 188 | hospitali#ed.mp. | 209062 |
| 189 | institutionali#ed.mp. | 15253 |
| 190 | or/176-189 [ Patients & related terms ] | 12354984 |
| 191 | 175 and 190 [ Hospitals + Nature + Patients ] | 19596 |
| 192 | exp Qualitative Research/ | 94485 |
| 193 | exp Communication Barriers/ | 2791 |
| 194 | "Surveys and Questionnaires"/ | 784794 |
| 195 | Evaluation Study/ | 51130 |
| 196 | Feasibility Studies/ | 142243 |
| 197 | Grounded Theory/ | 8606 |
| 198 | Health Care Surveys/ | 15231 |
| 199 | Health Impact Assessment/ | 6078 |
| 200 | Health Knowledge, Attitudes, Practice/ | 100744 |
| 201 | "Health Services Needs and Demand"/ | 147113 |
| 202 | Hermeneutics/ | 602 |
| 203 | Interview/ | 227348 |
| 204 | Narration/ | 15732 |
| 205 | Narrative Medicine/ | 375 |
| 206 | Needs Assessment/ | 25878 |
| 207 | Nursing Methodology Research/ | 14815 |
| 208 | Observational Study/ | 253271 |
| 209 | Patient Education as Topic/ | 100539 |
| 210 | Patient Health Questionnaire/ | 3156 |
| 211 | Patient Reported Outcome Measures/ | 32330 |
| 212 | Patient Satisfaction/ | 151380 |
| 213 | Personal Narrative/ | 46468 |
| 214 | Personal Satisfaction/ | 60866 |
| 215 | Pilot Projects/ | 123603 |
| 216 | exp Program Evaluation/ | 29180 |
| 217 | ((discourse* or discurs*) adj3 analys#s).mp. | 3655 |
| 218 | ((purpos* adj4 sampl*) or (focus adj group*)).mp. | 90604 |
| 219 | (account or accounts or unstructured or open-ended or open ended or text* or narrative*).mp. | 915784 |
| 220 | (action research or cooperative inquir* or co operative inquir* or co- operative inquir*).mp. | 6185 |
| 221 | (constant adj (comparative or comparison)).mp. | 6669 |
| 222 | (corbin* adj2 strauss*).mp. | 428 |
| 223 | (emic or etic or hermeneutic* or heuristic* or semiotic* or (data adj1 saturat*) or participant observ*).mp. | 34417 |
| 224 | (evaluat* adj2 (study or studies)).mp. | 369620 |
| 225 | (field adj (study or studies or research)).mp. | 25493 |
| 226 | (grounded adj (theor* or study or studies or research or analys#s)).mp. | 17029 |
| 227 | (humanistic or existential or experiential or paradigm*).mp. | 216501 |
| 228 | (life stor* or women* stor*).mp. | 1943 |
| 229 | (life world or life-world or conversation analys#s or personal experience* or theoretical saturation).mp. | 70532 |
| 230 | ((lived or life) adj experience*).mp. | 19114 |
| 231 | (merleau adj ponty*).mp. | 285 |
| 232 | (observational adj (study or studies or research)).mp. | 312912 |
| 233 | (social construct* or (postmodern* or post- structural*) or (post structural* or poststructural*) or post modern* or post-modern* or feminis* or interpret*).mp. | 591162 |
| 234 | (survey? or surveyed or surveying).mp. | 1639932 |
| 235 | (theme* or thematic).mp. | 171913 |
| 236 | (van adj kaam*).mp. | 46 |
| 237 | (van adj manen*).mp. | 567 |
| 238 | biographical method?.mp. | 43 |
| 239 | cluster sampl*.mp. | 10302 |
| 240 | colaizzi*.mp. | 984 |
| 241 | content analys#s.mp. | 43506 |
| 242 | ethnograph*.mp. | 14508 |
| 243 | ethnological research.mp. | 11 |
| 244 | ethnonursing.mp. | 128 |
| 245 | foucault*.mp. | 1028 |
| 246 | glaser*.mp. | 1174 |
| 247 | heidegger*.mp. | 877 |
| 248 | human science.mp. | 324 |
| 249 | narrative analys#s.mp. | 1787 |
| 250 | narrative review?.mp. | 18021 |
| 251 | observational method*.mp. | 2523 |
| 252 | phenomenol*.mp. | 37375 |
| 253 | qualitative.mp. | 384927 |
| 254 | questionnaire*.mp. | 1094589 |
| 255 | spiegelberg*.mp. | 167 |
| 256 | theoretical sampl*.mp. | 1058 |
| 257 | (client* adj2 (satisfaction or satisfied or satisfy*)).mp. | 2037 |
| 258 | (content analys* or thematic analys* or narrative analys*).mp. | 81756 |
| 259 | (ethnol* or ethnog* or ethnonurs* or emic or etic).mp. | 96234 |
| 260 | (feasib* adj2 (study or studies)).mp. | 166406 |
| 261 | (Grounded adj5 theor*).mp. | 18750 |
| 262 | (hermeneutic* or phenomenolog* or lived experience*).mp. | 45888 |
| 263 | (integrat* adj1 model?).mp. | 9150 |
| 264 | (meta-ethnog* or metaethnog* or meta-narrat* or metanarrat* or meta-interpret* or metainterpret*).mp. | 1066 |
| 265 | (metasynthes* or meta-synthes* or metasummar* or meta-summar* or metastud* or meta-stud*).ti,ab. | 1988 |
| 266 | (mixed adj2 method*).mp. | 38271 |
| 267 | (multiple adj1 perspective?).mp. | 1540 |
| 268 | (needs adj2 assessment*).mp. | 32320 |
| 269 | (patient?? adj2 (satisfaction or satisfied or satisfy*)).mp. | 176165 |
| 270 | (person?? adj2 (satisfaction or satisfied or satisfy*)).mp. | 1774 |
| 271 | (personal adj1 (story or stories)).mp. | 872 |
| 272 | (personal adj1 account???).mp. | 1444 |
| 273 | (program* adj3 evaluat*).mp. | 48578 |
| 274 | (qualitative adj5 metaanaly*).mp. | 18 |
| 275 | (qualitative adj5 meta-analy*).mp. | 1110 |
| 276 | (semantic? adj2 description?).mp. | 146 |
| 277 | (therapeutic adj1 model?).mp. | 1483 |
| 278 | (treatment? adj1 model?).mp. | 6687 |
| 279 | (unmet adj2 need?).mp. | 40800 |
| 280 | action research.ti,ab. | 5541 |
| 281 | contextual*.mp. | 52610 |
| 282 | focus group?.mp. | 67841 |
| 283 | frame work*.mp. | 954 |
| 284 | framework*.mp. | 372387 |
| 285 | giorgi*.mp. | 1076 |
| 286 | interview?.mp. | 483050 |
| 287 | mixed-method*.mp. | 35160 |
| 288 | multimethod inquir*.mp. | 1 |
| 289 | multi-method inquir*.mp. | 2 |
| 290 | multiperspective?.mp. | 180 |
| 291 | multi-perspective?.mp. | 296 |
| 292 | narrative?.mp. | 61505 |
| 293 | phenomenological*.mp. | 23356 |
| 294 | qualitative*.mp. | 436943 |
| 295 | qualitative.mp. | 384927 |
| 296 | questionnaire?.mp. | 1094218 |
| 297 | thematic.mp. | 54834 |
| 298 | theme.mp. | 33611 |
| 299 | themes.mp. | 107187 |
| 300 | or/192-299 [ Qualitative Research or Questionnaires & Related Terms ] | 6177819 |
| 301 | exp Clinical Trial/ | 1667495 |
| 302 | Clinical Trials, Phase III as Topic/ | 43135 |
| 303 | Comparative Study/ | 964330 |
| 304 | Controlled Clinical Trial/ | 464712 |
| 305 | Controlled Clinical Trials as Topic/ | 10737 |
| 306 | Cross-Sectional Studies/ | 318746 |
| 307 | Double-Blind Method/ | 167846 |
| 308 | Equivalence Trial/ | 123 |
| 309 | Equivalence Trials as Topic/ | 131 |
| 310 | exp Case-Control Studies/ | 198189 |
| 311 | exp Cohort Studies/ | 775548 |
| 312 | exp Randomized Controlled Trial/ | 687543 |
| 313 | exp Randomized Controlled Trials as Topic/ | 214837 |
| 314 | Longitudinal Studies/ | 144486 |
| 315 | Meta-Analysis as Topic/ | 35029 |
| 316 | Meta-Analysis/ | 230118 |
| 317 | Multicenter Studies as Topic/ | 34959 |
| 318 | Multicenter Study/ | 306269 |
| 319 | Observational Study/ | 253271 |
| 320 | Placebos/ | 328460 |
| 321 | Pragmatic Clinical Trial/ | 1378 |
| 322 | Pragmatic Clinical Trials as Topic/ | 214750 |
| 323 | Prospective Studies/ | 623795 |
| 324 | Retrospective Studies/ | 889745 |
| 325 | Systematic Review/ | 321304 |
| 326 | Systematic Reviews as Topic/ | 27739 |
| 327 | Validation Study/ | 93025 |
| 328 | ("phase 1" or "phase1" or "phase I").mp. | 143967 |
| 329 | ("phase 2" or "phase2" or "phase II").mp. | 198452 |
| 330 | ("phase 3" or "phase3" or "phase III").mp. | 154945 |
| 331 | ((multicenter* or multicentre* or multicentric) adj2 (trial? or study or studies)).mp. | 397244 |
| 332 | ((noninferiority or non-inferiority) adj4 (trial? or study or studies)).mp. | 9329 |
| 333 | ((single or double or triple or treble) adj3 (blind* or mask*)).mp. | 338934 |
| 334 | (case control* adj2 (study or studies)).mp. | 245844 |
| 335 | (comparative adj2 (trial? or study or studies)).mp. | 1066339 |
| 336 | (conceal* adj2 allocat*).mp. | 4048 |
| 337 | (controlled adj1 clinical adj2 (trial? or study or studies)).mp. | 508105 |
| 338 | (cross-sectional* adj2 (study or studies)).mp. | 514998 |
| 339 | (equivalen* adj4 (trial? or study or studies)).mp. | 7136 |
| 340 | (evaluation adj1 (study or studies)).mp. | 60865 |
| 341 | (longitudinal* adj2 (study or studies)).mp. | 221204 |
| 342 | (meta-anal* or metanal* or metaanal*).mp. | 363124 |
| 343 | (observational adj2 (trial? or study or studies)).mp. | 340731 |
| 344 | (overview? adj4 (review or reviews)).mp. | 24523 |
| 345 | (pragmatic adj2 (trial? or study or studies)).mp. | 5160 |
| 346 | (prospective* adj2 (study or studies)).mp. | 920239 |
| 347 | (retrospective* adj2 (study or studies)).mp. | 1267844 |
| 348 | (superiority adj4 (trial? or study or studies)).mp. | 6294 |
| 349 | (systematic adj4 (review or reviews or overview or overviews)).mp. | 426428 |
| 350 | (validation adj1 (study or studies)).mp. | 105344 |
| 351 | cohort*.mp. | 1347507 |
| 352 | placebo*.mp. | 495297 |
| 353 | qualitativ*.mp. | 437024 |
| 354 | quasirandom*.mp. | 243 |
| 355 | random*.mp. | 1978930 |
| 356 | research.ti,hw. | 1187827 |
| 357 | semiquantitative.mp. | 26407 |
| 358 | or/301-357 [ Studies ] | 8524611 |
| 359 | 300 or 358 [ Qualitative or Quantitative Research ] | 12153388 |
| 360 | 191 and 359 [ Hospitals + Nature + Patients + (Qualitative or Quantitative) ] | 10695 |
| 361 | limit 360 to english language | 10233 |
| 362 | limit 361 to (conference abstracts or (books or chapter or conference abstract or "conference review") or (book or book series or conference proceeding)) | 6017 |
| 363 | 361 not 362 | 4216 |
| 364 | conferenc*.so. | 624877 |
| 365 | 363 not 364 | 4212 |
| 366 | remove duplicates from 365 | 4180 |
| 367 | (exp animals/ or exp animal experimentation/ or nonhuman/) not ((exp animals/ or exp animal experimentation/ or nonhuman/) and exp human/) | 7583837 |
| 368 | 366 not 367 | 4134 |
| 369 | limit 366 to human | 3931 |
| 370 | 366 not (animal or animals or ape or apes or baboon or baboons or bat or bats or beagle or beagles or bird or birds or boar or boars or bonobo or bonobos or bovine or camel or camels or canine or canines or cat or cats or cattle or chick or chicks or chicken or chickens or chimpanzee or chimpanzees or dog or dogs or dromedary or dromedaries or duck or ducks or equine or equines or feline or felines or ferret or ferrets or frog or frogs or fowl or fowls or goat or goats or hamster or hamsters or hare or hares or hen or hens or horse or horses or lamb or lambs or livestock or macaque or macaques or mandrill or mandrills or mice or mink or minks or monkey or monkeys or mouse or murine or ovine or pig or pigs or piglet or piglets or poultry or porcine or orangutan or orangutans or rabbit or rabbits or rat or rats or rodent or rodents or sheep or swine or tamarin or tamarins or tiger or tigers or veterinary or veterinarian or veterinarians or waterfowl or waterfowls or weasel or weasels or veterinar* or fish or shellfish).ti,jw,hw. | 4035 |
| 371 | 366 and (human* or patient? or man or mankind or men or women or woman or adult*).ti,jw. | 1025 |
| 372 | 368 or 369 or 370 or 371 | 4147 |
| 373 | epigenetics/ or gene expression/ or gene overexpression/ or gene mutation/ or genetic marker/ or histopathology/ or human cell/ or human tissue/ or immunofluorescence/ or immunohistopathology/ or transcripteom*.hw. or ((cell* adj1 landscape*) or (mutat* adj1 landscape)).mp. [ To remove bench science citations from results ] | 4724082 |
| 374 | 372 not 373 [ Final Embase results ] | 3697 |

# CCTR

Cochrane Central Register of Controlled Trials 2014 to Present

| **#** | **Searches** | **Results** |
| --- | --- | --- |
| 1 | Environment/ and (physical or build* or built or hospital or hospitals or inpatient? or interior? or facility or facilities or architectur*).mp. | 147 |
| 2 | "Facility Design and Construction"/ | 25 |
| 3 | "Hospital Design and Construction"/ | 10 |
| 4 | "Interior Design and Furnishings"/ | 98 |
| 5 | "Patient’s Rooms"/ | 1 |
| 6 | Architecture/ | 6 |
| 7 | Bed Occupancy/ | 9 |
| 8 | Birthing Centers/ | 15 |
| 9 | Building Codes/ | 1 |
| 10 | Built Environment/ | 12 |
| 11 | Delivery Rooms/ | 74 |
| 12 | Environment Design/ | 113 |
| 13 | Environmental Medicine/ | 0 |
| 14 | Evidence-Based Facility Design/ | 1 |
| 15 | exp Hospitals/ | 3908 |
| 16 | exp Academic Medical Centers/ | 1829 |
| 17 | exp Intensive Care Units/ | 3858 |
| 18 | exp Medicine/ and (architect* or building? or department? or environment* or hospital or hospitals or landscape? or ward? or unit? or floor? or hospitali#ation? or facility or facilities or room or rooms or roomed or rooming).mp. | 3900 |
| 19 | Health Facilities, Proprietary/ | 2 |
| 20 | Health Facilities/ | 105 |
| 21 | Health Facility Environment/ | 53 |
| 22 | Hospital Bed Capacity/ | 14 |
| 23 | exp Hospital Units/ | 4371 |
| 24 | Hospitals, Proprietary/ | 2 |
| 25 | Universal Design/ | 0 |
| 26 | Waiting Rooms/ | 2 |
| 27 | architect*.in,hw. | 241 |
| 28 | (general adj3 (department? or ward? or unit? or floor? or hospitali#ation?)).mp. | 2818 |
| 29 | (healing adj1 (place or places)).mp. | 10 |
| 30 | (healing adj1 (space or spaces)).mp. | 4 |
| 31 | (health* adj1 environment?).ti,ab,jw. | 473 |
| 32 | ((health care or healthcare) adj1 design???).mp. | 109 |
| 33 | ((health care or healthcare) adj1 setting?).mp. | 1549 |
| 34 | (healthy adj1 (place? or space?)).mp. | 32 |
| 35 | (hospital? adj3 (department? or ward? or unit? or floor? or hospitali#ation?)).mp. | 19836 |
| 36 | (in-patient?? adj3 room?).mp. | 2987 |
| 37 | (inpatient?? adj3 room?).mp. | 99 |
| 38 | (patient?? adj3 room?).mp. | 2987 |
| 39 | (public? adj2 area?).mp. | 116 |
| 40 | (surgical adj3 (department? or ward? or unit? or floor? or hospitali#ation?)).mp. | 2926 |
| 41 | or/1-40 [ Hospitals or Health Care Environment ] | 39302 |
| 42 | Nature/ | 26 |
| 43 | Bays/ | 0 |
| 44 | Birds/ | 10 |
| 45 | Estuaries/ | 0 |
| 46 | Forests/ | 14 |
| 47 | Gardens/ | 8 |
| 48 | Horticulture/ | 0 |
| 49 | Horticultural Therapy/ | 18 |
| 50 | Lakes/ | 6 |
| 51 | "Oceans and Seas"/ | 20 |
| 52 | Plants/ | 45 |
| 53 | Ponds/ | 0 |
| 54 | Rivers/ | 6 |
| 55 | exp Songbirds/ | 4 |
| 56 | Trees/ | 89 |
| 57 | Vocalization, Animal/ | 5 |
| 58 | Wilderness/ | 2 |
| 59 | exp Auditory Perception/ and exp Nature/ | 0 |
| 60 | (access* adj2 natural).mp. | 19 |
| 61 | (access* adj2 nature).mp. | 6 |
| 62 | (acoust* adj3 access* adj3 (nature or natural)).mp. | 0 |
| 63 | (audib* adj3 access* adj3 (nature or natural)).mp. | 0 |
| 64 | (auditor* adj3 access* adj3 (nature or natural)).mp. | 0 |
| 65 | (commun??? adj2 nature).mp. | 16 |
| 66 | (biophili?? adj2 design*).mp. | 3 |
| 67 | (biophili?? adj2 imag*).mp. | 0 |
| 68 | (contact* adj2 nature).mp. | 28 |
| 69 | (experienc* adj2 nature).mp. | 68 |
| 70 | (green adj2 belt*).mp. | 1 |
| 71 | green infrastructur*.mp. | 0 |
| 72 | ((hear or hears or hearing or heard) adj3 (nature or natural)).mp. | 29 |
| 73 | ((hear or hears or hearing or heard) adj3 animal?).mp. | 6 |
| 74 | ((hear or hears or hearing or heard) adj3 bird?).mp. | 0 |
| 75 | ((hear or hears or hearing or heard) adj3 rain*).mp. | 1 |
| 76 | ((hear or hears or hearing or heard) adj3 water).mp. | 8 |
| 77 | ((hear or hears or hearing or heard) adj3 wind?).mp. | 0 |
| 78 | (hear* adj3 access* adj3 (nature or natural)).mp. | 0 |
| 79 | (listen* adj3 (nature or natural)).mp. | 35 |
| 80 | (listen* adj3 animal?).mp. | 0 |
| 81 | (listen* adj3 bird?).mp. | 2 |
| 82 | (listen* adj3 rain*).mp. | 2 |
| 83 | (listen* adj3 water?).mp. | 6 |
| 84 | (listen* adj3 wind?).mp. | 5 |
| 85 | (natural adj2 environ*).mp. | 450 |
| 86 | (natural adj2 imag*).mp. | 57 |
| 87 | (natural adj2 land*).mp. | 20 |
| 88 | (natural adj2 light*).mp. | 78 |
| 89 | (natural adj2 scene*).mp. | 57 |
| 90 | (natural adj2 setting*).mp. | 144 |
| 91 | (natural adj2 sound?).mp. | 51 |
| 92 | (natural adj2 spac*).mp. | 18 |
| 93 | (natural adj2 view*).mp. | 43 |
| 94 | (natural adj2 virtual*).mp. | 11 |
| 95 | (natural adj1 world?).mp. | 12 |
| 96 | (nature adj2 environ*).mp. | 56 |
| 97 | (nature adj2 imag*).mp. | 39 |
| 98 | (nature adj2 landscape?).mp. | 6 |
| 99 | (nature adj2 scene*).mp. | 31 |
| 100 | (nature adj2 simulat*).mp. | 8 |
| 101 | (nature?? adj2 sound?).mp. | 87 |
| 102 | (nature adj2 spac??).mp. | 3 |
| 103 | (nature adj2 stimul*).mp. | 66 |
| 104 | (nature adj2 view*).mp. | 55 |
| 105 | (nature adj2 virtual*).mp. | 19 |
| 106 | nature-based.mp. | 87 |
| 107 | (nature-based adj2 art?).mp. | 0 |
| 108 | ((near or nearby) adj3 (nature or natural)).mp. | 16 |
| 109 | (outdoor? adj2 (art or arts or artwork*)).mp. | 0 |
| 110 | (outdoor? adj2 imag*).mp. | 1 |
| 111 | (outdoor? adj2 photo*).mp. | 6 |
| 112 | (physical* adj3 access* adj3 (nature or natural)).mp. | 1 |
| 113 | (scenic adj2 view*).mp. | 0 |
| 114 | (visual* adj3 access* adj3 (nature or natural)).mp. | 0 |
| 115 | airscape?.mp. | 0 |
| 116 | artwork?.mp. | 41 |
| 117 | balconies.mp. | 2 |
| 118 | balcony.mp. | 4 |
| 119 | biophili???.mp. | 5 |
| 120 | birdsong?.mp. | 9 |
| 121 | "blue space?".mp. | 4 |
| 122 | bluespace?.mp. | 0 |
| 123 | cloudscape?.mp. | 0 |
| 124 | countryside?.mp. | 31 |
| 125 | courtyard?.mp. | 18 |
| 126 | daylight*.mp. | 283 |
| 127 | ecotherap*3.mp. | 0 |
| 128 | fauna.mp. | 11 |
| 129 | "fine art?".mp. | 16 |
| 130 | flower?.mp. | 594 |
| 131 | flowerbed?.mp. | 0 |
| 132 | (forest? not ("causal forest?" or "forest classifier?" or "forest plot?" or "random* forest?" or "wake forest" or "decision* forest?")).mp. | 536 |
| 133 | (garden or gardens).mp. | 360 |
| 134 | (green adj2 space?).mp. | 36 |
| 135 | greenbelt?.mp. | 3 |
| 136 | greenery.mp. | 8 |
| 137 | greenspace?.mp. | 7 |
| 138 | "green space?".mp. | 30 |
| 139 | horticultur*.mp. | 131 |
| 140 | ((landscape or landscapes or landscaping) not (landscape? adj2 (biologic* or regulatory or chang* or cultural* or genetic* or genomic* or global* or practice? or practise? or treatment? or trial? or national or online or economic* or online or techn* or fraught or risk? or contact or test*3 or service? or tele* or policy or politic* or remediat* or health* or institution* or insur* or underemploy* or unemploy*))).mp. [ Testing of added terms ] | 533 |
| 141 | (mural or murals).mp. | 167 |
| 142 | non-city.mp. | 3 |
| 143 | non-urban.mp. | 18 |
| 144 | non-window*.mp. | 1 |
| 145 | nonwindow*.mp. | 2 |
| 146 | "open space?".mp. | 32 |
| 147 | (outdoor? adj3 (life or panoram* or scene??? or space? or view? or vista?)).mp. | 33 |
| 148 | "outside world".mp. | 22 |
| 149 | (panoramic adj2 (scen* or view? or vista?)).mp. | 24 |
| 150 | parkland?.mp. | 106 |
| 151 | phytotherap*3.mp. | 4381 |
| 152 | (plant? adj1 (living or growing)).mp. | 22 |
| 153 | "public space?".mp. | 60 |
| 154 | riverscape?.mp. | 0 |
| 155 | (roof or roofs or rooftop?).mp. | 344 |
| 156 | scenery.mp. | 46 |
| 157 | seascape?.mp. | 3 |
| 158 | (terrace or terraces).mp. | 12 |
| 159 | (vista and (nature or natural)).mp. | 7 |
| 160 | (vistas and (nature or natural)).mp. | 0 |
| 161 | waterscape?.mp. | 0 |
| 162 | wilderness??.mp. | 35 |
| 163 | (wilds or wilderness).mp. | 35 |
| 164 | (((window or windows) not ((spss adj2 windows) or (statistics adj2 windows) or (time adj1 window?) or time-window? or "time window")) or (ibm adj4 windows)).mp. | 4977 |
| 165 | ((window or windows) not (hour-window? or minute-window? or (dissection* adj1 window?))).mp. | 6135 |
| 166 | ((window or windows) not (window? adj1 opportunit*)).mp. | 5712 |
| 167 | window-side?.mp. | 2 |
| 168 | windowside?.mp. | 0 |
| 169 | woods.mp. | 142 |
| 170 | Art/ | 58 |
| 171 | Paintings/ | 14 |
| 172 | "Pictorial Works as Topic"/ | 0 |
| 173 | Sculpture/ | 1 |
| 174 | or/42-173 [ Natural Environment & related terms or how to see them ] | 15562 |
| 175 | 41 and 174 [ Hospitals + Nature ] | 463 |
| 176 | exp Patients/ | 6011 |
| 177 | exp Hospitalization/ | 14603 |
| 178 | Length of Stay/ | 7375 |
| 179 | Patient-Centered Care/ | 664 |
| 180 | exp Patient Outcome Assessment/ | 319 |
| 181 | Patient Readmission/ | 1126 |
| 182 | (patient?? or inpatient??).mp. | 1107764 |
| 183 | ((person?? or client?) adj2 (centered or centred or centric)).mp. | 1319 |
| 184 | (hospital? adj3 (admit* or admis*)).mp. | 19148 |
| 185 | (hospital? adj3 (readmit* or readmis*)).mp. | 5672 |
| 186 | (hospital? adj3 (revisit* or re-visit*)).mp. | 56 |
| 187 | (length adj2 stay*3).mp. | 27851 |
| 188 | hospitali#ed.mp. | 18761 |
| 189 | institutionali#ed.mp. | 1262 |
| 190 | or/176-189 [ Patients & related terms ] | 1117634 |
| 191 | 175 and 190 [ Hospitals + Nature + Patients ] | 366 |
| 192 | 191 not (abstract or addresses or bibliography or biography or book or book article or book book or book note or "book review" or book series article or book series article in press or book series chapter or book series conference paper or book series letter or "book series review" or book series short survey or chapter or conference or conference abstract or conference abstract placebo controlled partly blinded crossover study in 12 sle patients or conference proceeding or "conference review" or journal conference abstract or "journal conference review" or monograph).pt. | 327 |
| 193 | 192 not conference*.so. | 326 |
| 194 | 193 not "http?://clinicaltrial*".so. | 196 |
| 195 | 194 not "http?://www*".so. | 196 |
| 196 | 195 not "http?://trials*".so. | 172 |
| 197 | limit 196 to english language | 147 |
| 198 | remove duplicates from 197 | 140 |

# CDSR

Cochrane Database of Systematic Reviews 2005 to Present

| **#** | **Searches** | **Results** |
| --- | --- | --- |
| 1 | (general adj3 (department? or ward? or unit? or floor? or hospitali#ation?)).ti,ab. | 16 |
| 2 | (healing adj1 (place or places)).ti,ab. | 0 |
| 3 | (healing adj1 (space or spaces)).ti,ab. | 0 |
| 4 | (health* adj1 environment?).ti,ab. | 2 |
| 5 | ((health care or healthcare) adj1 design???).ti,ab. | 0 |
| 6 | ((health care or healthcare) adj1 setting?).ti,ab. | 73 |
| 7 | (healthy adj1 (place? or space?)).ti,ab. | 0 |
| 8 | (hospital? and (department? or ward? or unit? or floor? or hospitali#ation?)).ti,ab. | 391 |
| 9 | (in-patient?? adj3 room?).ti,ab. | 0 |
| 10 | (inpatient?? adj3 room?).ti,ab. | 0 |
| 11 | (patient?? adj3 room?).ti,ab. | 0 |
| 12 | (public? adj2 area?).ti,ab. | 0 |
| 13 | (surgical adj3 (department? or ward? or unit? or floor? or hospitali#ation?)).ti,ab. | 3 |
| 14 | (medical adj3 (department? or ward? or unit? or floor? or hospitali#ation?)).ti,ab. | 10 |
| 15 | or/1-14 [ Hospitals ] | 465 |
| 16 | (access* adj2 natural).ti,ab. | 0 |
| 17 | (access* adj2 nature).ti,ab. | 1 |
| 18 | (acoust* adj3 access* adj3 (nature or natural)).ti,ab. | 0 |
| 19 | (audib* adj3 access* adj3 (nature or natural)).ti,ab. | 0 |
| 20 | (auditor* adj3 access* adj3 (nature or natural)).ti,ab. | 0 |
| 21 | (commun??? adj2 nature).ti,ab. | 0 |
| 22 | (biophili?? adj2 design*).ti,ab. | 0 |
| 23 | (biophili?? adj2 imag*).ti,ab. | 0 |
| 24 | (contact* adj2 nature).ti,ab. | 0 |
| 25 | (experienc* adj2 nature).ti,ab. | 0 |
| 26 | (green adj2 belt*).ti,ab. | 0 |
| 27 | green infrastructur*.ti,ab. | 0 |
| 28 | ((hear or hears or hearing or heard) adj3 (nature or natural)).ti,ab. | 0 |
| 29 | ((hear or hears or hearing or heard) adj3 animal?).ti,ab. | 0 |
| 30 | ((hear or hears or hearing or heard) adj3 bird?).ti,ab. | 0 |
| 31 | ((hear or hears or hearing or heard) adj3 rain*).ti,ab. | 0 |
| 32 | ((hear or hears or hearing or heard) adj3 water).ti,ab. | 0 |
| 33 | ((hear or hears or hearing or heard) adj3 wind?).ti,ab. | 0 |
| 34 | (hear* adj3 access* adj3 (nature or natural)).ti,ab. | 0 |
| 35 | (listen* adj3 (nature or natural)).ti,ab. | 0 |
| 36 | (listen* adj3 animal?).ti,ab. | 0 |
| 37 | (listen* adj3 bird?).ti,ab. | 0 |
| 38 | (listen* adj3 rain*).ti,ab. | 0 |
| 39 | (listen* adj3 water?).ti,ab. | 0 |
| 40 | (listen* adj3 wind?).ti,ab. | 0 |
| 41 | (natural adj2 environ*).ti,ab. | 3 |
| 42 | (natural adj2 imag*).ti,ab. | 0 |
| 43 | (natural adj2 land*).ti,ab. | 0 |
| 44 | (natural adj2 light*).ti,ab. | 0 |
| 45 | (natural adj2 scene*).ti,ab. | 0 |
| 46 | (natural adj2 setting*).ti,ab. | 1 |
| 47 | (natural adj2 sound?).ti,ab. | 0 |
| 48 | (natural adj2 spac*).ti,ab. | 0 |
| 49 | (natural adj2 view*).ti,ab. | 0 |
| 50 | (natural adj2 virtual*).ti,ab. | 0 |
| 51 | (natural adj1 world?).ti,ab. | 0 |
| 52 | (nature adj2 environ*).ti,ab. | 0 |
| 53 | (nature adj2 imag*).ti,ab. | 0 |
| 54 | (nature adj2 landscape?).ti,ab. | 0 |
| 55 | (nature adj2 scene*).ti,ab. | 0 |
| 56 | (nature adj2 simulat*).ti,ab. | 0 |
| 57 | (nature?? adj2 sound?).ti,ab. | 0 |
| 58 | (nature adj2 spac??).ti,ab. | 0 |
| 59 | (nature adj2 stimul*).ti,ab. | 0 |
| 60 | nature-theme?.ti,ab. | 0 |
| 61 | (nature adj1 theme?).ti,ab. | 0 |
| 62 | (nature adj2 view*).ti,ab. | 0 |
| 63 | (nature adj2 virtual*).ti,ab. | 0 |
| 64 | nature-based.ti,ab. | 0 |
| 65 | (nature-based adj2 art?).ti,ab. | 0 |
| 66 | ((near or nearby) adj3 (nature or natural)).ti,ab. | 0 |
| 67 | (outdoor? adj2 (art or arts or artwork*)).ti,ab. | 0 |
| 68 | (outdoor? adj2 imag*).ti,ab. | 0 |
| 69 | (outdoor? adj2 photo*).ti,ab. | 0 |
| 70 | (physical* adj3 access* adj3 (nature or natural)).ti,ab. | 0 |
| 71 | (scenic adj2 view*).ti,ab. | 0 |
| 72 | (visual* adj3 access* adj3 (nature or natural)).ti,ab. | 0 |
| 73 | airscape?.ti,ab. | 0 |
| 74 | artwork?.ti,ab. | 0 |
| 75 | balconies.ti,ab. | 0 |
| 76 | balcony.ti,ab. | 0 |
| 77 | biophili???.ti,ab. | 0 |
| 78 | birdsong?.ti,ab. | 0 |
| 79 | "blue space?".ti,ab. | 0 |
| 80 | bluespace?.ti,ab. | 0 |
| 81 | cloudscape?.ti,ab. | 0 |
| 82 | countryside?.ti,ab. | 0 |
| 83 | courtyard?.ti,ab. | 0 |
| 84 | daylight*.ti,ab. | 1 |
| 85 | ecotherap*3.ti,ab. | 0 |
| 86 | fauna.ti,ab. | 0 |
| 87 | "fine art?".ti,ab. | 0 |
| 88 | flower?.ti,ab. | 4 |
| 89 | flowerbed?.ti,ab. | 0 |
| 90 | (forest? not ("causal forest?" or "forest classifier?" or "forest plot?" or "random* forest?" or "wake forest" or "decision* forest?")).ti,ab. | 3 |
| 91 | (garden or gardens).ti,ab. | 1 |
| 92 | (green adj2 space?).ti,ab. | 0 |
| 93 | greenbelt?.ti,ab. | 0 |
| 94 | greenery.ti,ab. | 0 |
| 95 | greenspace?.ti,ab. | 0 |
| 96 | "green space?".ti,ab. | 0 |
| 97 | horticultur*.ti,ab. | 1 |
| 98 | ((landscape or landscapes or landscaping) not (landscape? adj2 (biologic* or regulatory or chang* or cultural* or genetic* or genomic* or global* or practice? or practise? or treatment? or trial? or national or online or economic* or online or techn* or fraught or risk? or contact or test*3 or service? or tele* or policy or politic* or remediat* or health* or institution* or insur* or underemploy* or unemploy*))).ti,ab. [ Testing of added terms ] | 1 |
| 99 | (mural or murals).ti,ab. | 0 |
| 100 | non-city.ti,ab. | 0 |
| 101 | non-urban.ti,ab. | 0 |
| 102 | non-window*.ti,ab. | 0 |
| 103 | nonwindow*.ti,ab. | 0 |
| 104 | "open space?".ti,ab. | 0 |
| 105 | (outdoor? adj3 (life or panoram* or scene??? or space? or view? or vista?)).ti,ab. | 1 |
| 106 | "outside world".ti,ab. | 1 |
| 107 | (panoramic adj2 (scen* or view? or vista?)).ti,ab. | 0 |
| 108 | parkland?.ti,ab. | 0 |
| 109 | phytotherap*3.ti,ab. | 6 |
| 110 | (plant? adj1 (living or growing)).ti,ab. | 0 |
| 111 | "public space?".ti,ab. | 0 |
| 112 | riverscape?.ti,ab. | 0 |
| 113 | (roof or roofs or rooftop?).ti,ab. | 3 |
| 114 | scenery.ti,ab. | 0 |
| 115 | seascape?.ti,ab. | 0 |
| 116 | (terrace or terraces).ti,ab. | 0 |
| 117 | (vista and (nature or natural)).ti,ab. | 0 |
| 118 | (vistas and (nature or natural)).ti,ab. | 0 |
| 119 | waterscape?.ti,ab. | 0 |
| 120 | wilderness??.ti,ab. | 0 |
| 121 | (wilds or wilderness).ti,ab. | 0 |
| 122 | (((window or windows) not ((spss adj2 windows) or (statistics adj2 windows) or (time adj1 window?) or time-window? or "time window")) or (ibm adj4 windows)).ti,ab. | 12 |
| 123 | ((window or windows) not (hour-window? or minute-window? or (dissection* adj1 window?))).ti,ab. | 16 |
| 124 | ((window or windows) not (window? adj1 opportunit*)).ti,ab. | 16 |
| 125 | window-side?.ti,ab. | 0 |
| 126 | windowside?.ti,ab. | 0 |
| 127 | woods.ti,ab. | 0 |
| 128 | or/15-127 [ Nature ] | 502 |
| 129 | 13 and 128 [ Hospitals + Nature ] | 3 |
| 130 | (patient?? or inpatient??).ti,ab. | 4233 |
| 131 | ((person?? or client?) adj2 (centered or centred or centric)).ti,ab. | 15 |
| 132 | (hospital? adj3 (admit* or admis*)).ti,ab. | 253 |
| 133 | (hospital? adj3 (readmit* or readmis*)).ti,ab. | 35 |
| 134 | (hospital? adj3 (revisit* or re-visit*)).ti,ab. | 0 |
| 135 | (length adj2 stay*3).ti,ab. | 159 |
| 136 | hospitali#ed.ti,ab. | 130 |
| 137 | institutionali#ed.ti,ab. | 6 |
| 138 | or/130-137 [ Patients ] | 4424 |
| 139 | 129 and 138 [ Hospitals + Nature + Patients ] | 3 |

# APA PsycINFO

APA PsycInfo 1806 to November Week 3 2021

| **#** | **Searches** | **Results** |
| --- | --- | --- |
| 1 | Environment/ and (physical or build* or built or hospital or hospitals or inpatient? or interior? or facility or facilities or architectur*).mp. | 3855 |
| 2 | Architecture/ | 2078 |
| 3 | Built Environment/ | 1145 |
| 4 | facility environment/ | 801 |
| 5 | exp Hospitals/ | 25765 |
| 6 | hospital environment/ | 1901 |
| 7 | exp Medicine/ and (architect* or building? or department? or environment* or hospital or hospitals or landscape? or ward? or unit? or floor? or hospitali#ation? or facility or facilities or room or rooms or roomed or rooming).mp. | 86115 |
| 8 | exp treatment facilities/ | 57171 |
| 9 | architect*.in,hw. | 4759 |
| 10 | (general adj3 (department? or ward? or unit? or floor? or hospitali#ation?)).mp. | 2915 |
| 11 | (healing adj1 (place or places)).mp. | 23 |
| 12 | (healing adj1 (space or spaces)).mp. | 40 |
| 13 | (health* adj1 environment?).ti,ab,jw. | 1981 |
| 14 | ((health care or healthcare) adj1 design???).mp. | 196 |
| 15 | ((health care or healthcare) adj1 setting?).mp. | 5964 |
| 16 | (healthy adj1 (place? or space?)).mp. | 54 |
| 17 | (hospital? and (department? or ward? or unit? or floor? or hospitali#ation?)).mp. | 48408 |
| 18 | (in-patient?? adj3 room?).mp. | 35 |
| 19 | (inpatient?? adj3 room?).mp. | 162 |
| 20 | (patient?? adj3 room?).mp. | 785 |
| 21 | (public? adj2 area?).mp. | 754 |
| 22 | (surgical adj3 (department? or ward? or unit? or floor? or hospitali#ation?)).mp. | 1354 |
| 23 | (medical adj3 (department? or ward? or unit? or floor? or hospitali#ation?)).ti,ab. | 3635 |
| 24 | or/1-23 [ Hospitals ] | 178992 |
| 25 | Birds/ | 14469 |
| 26 | Horticulture/ | 141 |
| 27 | "nature (environment)"/ | 2222 |
| 28 | environmental enrichment/ | 858 |
| 29 | Plants/ | 1337 |
| 30 | (access* adj2 natural).mp. | 118 |
| 31 | (access* adj2 nature).mp. | 139 |
| 32 | (acoust* adj3 access* adj3 (nature or natural)).mp. | 0 |
| 33 | (audib* adj3 access* adj3 (nature or natural)).mp. | 0 |
| 34 | (auditor* adj3 access* adj3 (nature or natural)).mp. | 0 |
| 35 | (commun??? adj2 nature).mp. | 319 |
| 36 | (biophili?? adj2 design*).mp. | 16 |
| 37 | (biophili?? adj2 imag*).mp. | 0 |
| 38 | (contact* adj2 nature).mp. | 267 |
| 39 | (experienc* adj2 nature).mp. | 958 |
| 40 | (green adj2 belt*).mp. | 8 |
| 41 | green infrastructur*.mp. | 20 |
| 42 | ((hear or hears or hearing or heard) adj3 (nature or natural)).mp. | 99 |
| 43 | ((hear or hears or hearing or heard) adj3 animal?).mp. | 881 |
| 44 | ((hear or hears or hearing or heard) adj3 bird?).mp. | 82 |
| 45 | ((hear or hears or hearing or heard) adj3 rain*).mp. | 1 |
| 46 | ((hear or hears or hearing or heard) adj3 water).mp. | 21 |
| 47 | ((hear or hears or hearing or heard) adj3 wind?).mp. | 8 |
| 48 | (hear* adj3 access* adj3 (nature or natural)).mp. | 1 |
| 49 | (listen* adj3 (nature or natural)).mp. | 188 |
| 50 | (listen* adj3 animal?).mp. | 18 |
| 51 | (listen* adj3 bird?).mp. | 12 |
| 52 | (listen* adj3 rain*).mp. | 6 |
| 53 | (listen* adj3 water?).mp. | 5 |
| 54 | (listen* adj3 wind?).mp. | 5 |
| 55 | (natural adj2 environ*).mp. | 4944 |
| 56 | (natural adj2 imag*).mp. | 957 |
| 57 | (natural adj2 land*).mp. | 200 |
| 58 | (natural adj2 light*).mp. | 247 |
| 59 | (natural adj2 scene*).mp. | 1422 |
| 60 | (natural adj2 setting*).mp. | 2313 |
| 61 | (natural adj2 sound?).mp. | 399 |
| 62 | (natural adj2 spac*).mp. | 113 |
| 63 | (natural adj2 view*).mp. | 526 |
| 64 | (natural adj2 virtual*).mp. | 30 |
| 65 | (natural adj1 world?).mp. | 1443 |
| 66 | (nature adj2 environ*).mp. | 2677 |
| 67 | (nature adj2 imag*).mp. | 337 |
| 68 | (nature adj2 landscape?).mp. | 38 |
| 69 | (nature adj2 scene*).mp. | 101 |
| 70 | (nature adj2 simulat*).mp. | 47 |
| 71 | (nature?? adj2 sound?).mp. | 126 |
| 72 | (nature adj2 spac??).mp. | 108 |
| 73 | (nature adj2 stimul*).mp. | 255 |
| 74 | nature-theme?.mp. | 14 |
| 75 | (nature adj1 theme?).mp. | 21 |
| 76 | (nature adj2 view*).mp. | 465 |
| 77 | (nature adj2 virtual*).mp. | 85 |
| 78 | nature-based.mp. | 437 |
| 79 | (nature-based adj2 art?).mp. | 3 |
| 80 | ((near or nearby) adj3 (nature or natural)).mp. | 119 |
| 81 | (outdoor? adj2 (art or arts or artwork*)).mp. | 21 |
| 82 | (outdoor? adj2 imag*).mp. | 23 |
| 83 | (outdoor? adj2 photo*).mp. | 13 |
| 84 | (physical* adj3 access* adj3 (nature or natural)).mp. | 0 |
| 85 | (scenic adj2 view*).mp. | 7 |
| 86 | (visual* adj3 access* adj3 (nature or natural)).mp. | 3 |
| 87 | airscape?.mp. | 1 |
| 88 | artwork?.mp. | 2181 |
| 89 | balconies.mp. | 22 |
| 90 | balcony.mp. | 70 |
| 91 | biophili???.mp. | 180 |
| 92 | birdsong?.mp. | 667 |
| 93 | "blue space?".mp. | 54 |
| 94 | bluespace?.mp. | 8 |
| 95 | cloudscape?.mp. | 0 |
| 96 | countryside?.mp. | 533 |
| 97 | courtyard?.mp. | 71 |
| 98 | daylight*.mp. | 1178 |
| 99 | ecotherap*3.mp. | 69 |
| 100 | fauna.mp. | 188 |
| 101 | "fine art?".mp. | 881 |
| 102 | flower?.mp. | 2509 |
| 103 | flowerbed?.mp. | 5 |
| 104 | (forest? not ("causal forest?" or "forest classifier?" or "forest plot?" or "random* forest?" or "wake forest" or "decision* forest?")).mp. | 4404 |
| 105 | (garden or gardens).mp. | 2579 |
| 106 | (green adj2 space?).mp. | 414 |
| 107 | greenbelt?.mp. | 10 |
| 108 | greenery.mp. | 83 |
| 109 | greenspace?.mp. | 88 |
| 110 | "green space?".mp. | 392 |
| 111 | horticultur*.mp. | 546 |
| 112 | ((landscape or landscapes or landscaping) not (landscape? adj2 (biologic* or regulatory or chang* or cultural* or genetic* or genomic* or global* or practice? or practise? or treatment? or trial? or national or online or economic* or online or techn* or fraught or risk? or contact or test*3 or service? or tele* or policy or politic* or remediat* or health* or institution* or insur* or underemploy* or unemploy*))).mp. | 11172 |
| 113 | (mural or murals).mp. | 314 |
| 114 | non-city.mp. | 5 |
| 115 | non-urban.mp. | 274 |
| 116 | non-window*.mp. | 1 |
| 117 | nonwindow*.mp. | 1 |
| 118 | "open space?".mp. | 769 |
| 119 | (outdoor? adj3 (life or panoram* or scene??? or space? or view? or vista?)).mp. | 442 |
| 120 | "outside world".mp. | 1695 |
| 121 | (panoramic adj2 (scen* or view? or vista?)).mp. | 167 |
| 122 | parkland?.mp. | 66 |
| 123 | phytotherap*3.mp. | 1127 |
| 124 | (plant? adj1 (living or growing)).mp. | 43 |
| 125 | "public space?".mp. | 1811 |
| 126 | riverscape?.mp. | 2 |
| 127 | (roof or roofs or rooftop?).mp. | 691 |
| 128 | scenery.mp. | 362 |
| 129 | seascape?.mp. | 32 |
| 130 | (terrace or terraces).mp. | 156 |
| 131 | (vista and (nature or natural)).mp. | 86 |
| 132 | (vistas and (nature or natural)).mp. | 56 |
| 133 | waterscape?.mp. | 15 |
| 134 | wilderness??.mp. | 1194 |
| 135 | (wilds or wilderness).mp. | 1225 |
| 136 | (((window or windows) not ((spss adj2 windows) or (statistics adj2 windows) or (time adj1 window?) or time-window? or "time window")) or (ibm adj4 windows)).mp. | 14019 |
| 137 | ((window or windows) not (hour-window? or minute-window? or (dissection* adj1 window?))).mp. | 17313 |
| 138 | ((window or windows) not (window? adj1 opportunit*)).mp. | 17350 |
| 139 | window-side?.mp. | 1 |
| 140 | windowside?.mp. | 0 |
| 141 | woods.mp. | 1344 |
| 142 | Art/ | 6825 |
| 143 | or/25-142 [ Nature ] | 90375 |
| 144 | 24 and 143 [ Hospitals + Nature ] | 3799 |
| 145 | exp Patients/ | 101645 |
| 146 | exp Hospitalization/ | 24749 |
| 147 | Length of Stay/ | 4319 |
| 148 | Patient-Centered Care/ | 344 |
| 149 | (patient?? or inpatient??).mp. | 800379 |
| 150 | ((person?? or client?) adj2 (centered or centred or centric)).mp. | 11830 |
| 151 | (hospital? adj3 (admit* or admis*)).mp. | 13669 |
| 152 | (hospital? adj3 (readmit* or readmis*)).mp. | 2161 |
| 153 | (hospital? adj3 (revisit* or re-visit*)).mp. | 26 |
| 154 | (length adj2 stay*3).mp. | 5814 |
| 155 | hospitali#ed.mp. | 32317 |
| 156 | institutionali#ed.mp. | 11472 |
| 157 | or/145-156 [ Patients ] | 834458 |
| 158 | 144 and 157 [ Hospitals + Nature + Patients ] | 1125 |
| 159 | exp Qualitative Research/ | 18164 |
| 160 | Qualitative Study/ | 0 |
| 161 | exp Communication Barriers/ | 858 |
| 162 | Grounded Theory/ | 4132 |
| 163 | Hermeneutics/ | 2306 |
| 164 | Needs Assessment/ | 4432 |
| 165 | Patient Reported Outcome Measures/ | 410 |
| 166 | Patient Satisfaction/ | 5922 |
| 167 | exp Program Evaluation/ | 21367 |
| 168 | ((discourse* or discurs*) adj3 analys#s).mp. | 15200 |
| 169 | ((purpos* adj4 sampl*) or (focus adj group*)).mp. | 51500 |
| 170 | (account or accounts or unstructured or open-ended or open ended or text* or narrative*).mp. | 382806 |
| 171 | (action research or cooperative inquir* or co operative inquir* or co- operative inquir*).mp. | 10500 |
| 172 | (constant adj (comparative or comparison)).mp. | 5195 |
| 173 | (corbin* adj2 strauss*).mp. | 710 |
| 174 | (emic or etic or hermeneutic* or heuristic* or semiotic* or (data adj1 saturat*) or participant observ*).mp. | 39635 |
| 175 | (evaluat* adj2 (study or studies)).mp. | 34437 |
| 176 | (field adj (study or studies or research)).mp. | 10910 |
| 177 | (grounded adj (theor* or study or studies or research or analys#s)).mp. | 18314 |
| 178 | (humanistic or existential or experiential or paradigm*).mp. | 146598 |
| 179 | (life stor* or women* stor*).mp. | 4448 |
| 180 | (life world or life-world or conversation analys#s or personal experience* or theoretical saturation).mp. | 16332 |
| 181 | ((lived or life) adj experience*).mp. | 51513 |
| 182 | (merleau adj ponty*).mp. | 962 |
| 183 | (observational adj (study or studies or research)).mp. | 12630 |
| 184 | (social construct* or (postmodern* or post- structural*) or (post structural* or poststructural*) or post modern* or post-modern* or feminis* or interpret*).mp. | 253450 |
| 185 | (survey? or surveyed or surveying).mp. | 368034 |
| 186 | (theme* or thematic).mp. | 152033 |
| 187 | (van adj kaam*).mp. | 504 |
| 188 | (van adj manen*).mp. | 585 |
| 189 | biographical method?.mp. | 93 |
| 190 | cluster sampl*.mp. | 1792 |
| 191 | colaizzi*.mp. | 625 |
| 192 | content analys#s.mp. | 32045 |
| 193 | ethnograph*.mp. | 31866 |
| 194 | ethnological research.mp. | 11 |
| 195 | ethnonursing.mp. | 66 |
| 196 | foucault*.mp. | 3156 |
| 197 | glaser*.mp. | 1377 |
| 198 | heidegger*.mp. | 1950 |
| 199 | human science.mp. | 664 |
| 200 | narrative analys#s.mp. | 3082 |
| 201 | narrative review?.mp. | 3037 |
| 202 | observational method*.mp. | 1200 |
| 203 | phenomenol*.mp. | 49405 |
| 204 | qualitative.mp. | 191625 |
| 205 | questionnaire*.mp. | 446603 |
| 206 | spiegelberg*.mp. | 19 |
| 207 | theoretical sampl*.mp. | 643 |
| 208 | (client* adj2 (satisfaction or satisfied or satisfy*)).mp. | 7970 |
| 209 | (content analys* or thematic analys* or narrative analys*).mp. | 53680 |
| 210 | (ethnol* or ethnog* or ethnonurs* or emic or etic).mp. | 36480 |
| 211 | (feasib* adj2 (study or studies)).mp. | 3204 |
| 212 | (Grounded adj5 theor*).mp. | 22842 |
| 213 | (hermeneutic* or phenomenolog* or lived experience*).mp. | 64126 |
| 214 | (integrat* adj1 model?).mp. | 7641 |
| 215 | (meta-ethnog* or metaethnog* or meta-narrat* or metanarrat* or meta-interpret* or metainterpret*).mp. | 857 |
| 216 | (metasynthes* or meta-synthes* or metasummar* or meta-summar* or metastud* or meta-stud*).ti,ab. | 1154 |
| 217 | (mixed adj2 method*).mp. | 30257 |
| 218 | (multiple adj1 perspective?).mp. | 2626 |
| 219 | (needs adj2 assessment*).mp. | 8278 |
| 220 | (patient?? adj2 (satisfaction or satisfied or satisfy*)).mp. | 7147 |
| 221 | (person?? adj2 (satisfaction or satisfied or satisfy*)).mp. | 1511 |
| 222 | (personal adj1 (story or stories)).mp. | 1687 |
| 223 | (personal adj1 account???).mp. | 2179 |
| 224 | (program* adj3 evaluat*).mp. | 34846 |
| 225 | (qualitative adj5 metaanaly*).mp. | 3 |
| 226 | (qualitative adj5 meta-analy*).mp. | 315 |
| 227 | (semantic? adj2 description?).mp. | 129 |
| 228 | (therapeutic adj1 model?).mp. | 992 |
| 229 | (treatment? adj1 model?).mp. | 4598 |
| 230 | (unmet adj2 need?).mp. | 6075 |
| 231 | action research.ti,ab. | 8851 |
| 232 | contextual*.mp. | 69307 |
| 233 | focus group?.mp. | 42369 |
| 234 | frame work*.mp. | 259 |
| 235 | framework*.mp. | 215986 |
| 236 | giorgi*.mp. | 1057 |
| 237 | interview?.mp. | 387413 |
| 238 | mixed-method*.mp. | 29572 |
| 239 | multimethod inquir*.mp. | 4 |
| 240 | multi-method inquir*.mp. | 3 |
| 241 | multiperspective?.mp. | 161 |
| 242 | multi-perspective?.mp. | 256 |
| 243 | narrative?.mp. | 76502 |
| 244 | phenomenological*.mp. | 36173 |
| 245 | qualitative*.mp. | 208644 |
| 246 | qualitative.mp. | 191625 |
| 247 | questionnaire?.mp. | 446524 |
| 248 | thematic.mp. | 35379 |
| 249 | theme.mp. | 32228 |
| 250 | themes.mp. | 105440 |
| 251 | exp Quantitative Research/ | 3661 |
| 252 | exp Clinical Trial/ | 13018 |
| 253 | exp Randomized Controlled Trial/ | 1054 |
| 254 | Longitudinal Studies/ | 15936 |
| 255 | Meta-Analysis/ | 5086 |
| 256 | Prospective Studies/ | 978 |
| 257 | Retrospective Studies/ | 720 |
| 258 | Systematic Review/ | 644 |
| 259 | ("phase 1" or "phase1" or "phase I").mp. | 6226 |
| 260 | ("phase 2" or "phase2" or "phase II").mp. | 6782 |
| 261 | ("phase 3" or "phase3" or "phase III").mp. | 3708 |
| 262 | ((multicenter* or multicentre*) adj2 (trial? or study or studies)).mp. | 4887 |
| 263 | ((noninferiority or non-inferiority) adj4 (trial? or study or studies)).mp. | 349 |
| 264 | ((single or double or triple or treble) adj3 (blind* or mask*)).mp. | 27784 |
| 265 | (case control* adj2 (study or studies)).mp. | 9014 |
| 266 | (comparative adj2 (trial? or study or studies)).mp. | 19675 |
| 267 | (conceal* adj2 allocat*).mp. | 222 |
| 268 | (controlled adj1 clinical adj2 (trial? or study or studies)).mp. | 3648 |
| 269 | (cross-sectional* adj2 (study or studies)).mp. | 38935 |
| 270 | (equivalen* adj4 (trial? or study or studies)).mp. | 1291 |
| 271 | (evaluation adj1 (study or studies)).mp. | 3249 |
| 272 | (longitudinal* adj2 (study or studies)).mp. | 74542 |
| 273 | (meta-anal* or metanal* or metaanal*).mp. | 44871 |
| 274 | (observational adj2 (trial? or study or studies)).mp. | 14154 |
| 275 | (overview? adj4 (review or reviews)).mp. | 1818 |
| 276 | (pragmatic adj2 (trial? or study or studies)).mp. | 826 |
| 277 | (prospective* adj2 (study or studies)).mp. | 29785 |
| 278 | (retrospective* adj2 (study or studies)).mp. | 12934 |
| 279 | (superiority adj4 (trial? or study or studies)).mp. | 495 |
| 280 | (systematic adj4 (review or reviews or overview or overviews)).mp. | 41617 |
| 281 | (validation adj1 (study or studies)).mp. | 5765 |
| 282 | cohort*.mp. | 97117 |
| 283 | placebo*.mp. | 43616 |
| 284 | qualitativ*.mp. | 208702 |
| 285 | quasirandom*.mp. | 41 |
| 286 | random*.mp. | 230116 |
| 287 | research.ti,hw,pt. | 113526 |
| 288 | semiquantitative.mp. | 484 |
| 289 | or/159-288 [ Qualitative or Quantitative ] | 2359749 |
| 290 | 158 and 289 [ Hospitals + Nature + Patients + (Qualitative or Quantitative) ] | 625 |
| 291 | limit 290 to english language | 574 |
| 292 | limit 291 to human | 563 |
| 293 | limit 292 to ("0200 book" or "0240 authored book" or "0280 edited book" or "0300 encyclopedia" or "0400 dissertation abstract" or "0500 electronic collection" or (classic book or conference proceedings or "handbook/manual" or reference book or "textbook/study guide")) | 136 |
| 294 | 292 not 293 | 427 |
| 295 | limit 292 to ("0100 journal" or "0110 peer-reviewed journal" or "0120 non-peer-reviewed journal" or "0130 peer-reviewed status unknown") | 427 |
| 296 | 294 or 295 | 427 |
| 297 | 296 not ("0*" or "1*" or "2*" or "3*" or "4*" or "5*" or "6*" or "7*" or "8*" or "9*").pm. | 116 |
| 298 | remove duplicates from 297 | 116 |

# Ovid EmCare Nursing

Ovid Emcare Nursing 1995 to Present

| **#** | **Searches** | **Results** |
| --- | --- | --- |
| 1 | Environment/ and (physical or build* or built or hospital or hospitals or inpatient? or interior? or facility or facilities or architectur*).mp. | 19077 |
| 2 | "Facility Design and Construction"/ | 987 |
| 3 | "Hospital Design and Construction"/ | 1263 |
| 4 | "Interior Design and Furnishings"/ | 1370 |
| 5 | "Patient’s Rooms"/ | 29327 |
| 6 | Architecture/ | 6976 |
| 7 | Bed Occupancy/ | 1405 |
| 8 | Birthing Centers/ | 2018 |
| 9 | Building Codes/ | 1125 |
| 10 | Built Environment/ | 710 |
| 11 | Delivery Rooms/ | 1686 |
| 12 | Environment Design/ | 1318 |
| 13 | Environmental Medicine/ | 66 |
| 14 | Evidence-Based Facility Design/ | 1263 |
| 15 | exp Hospitals/ | 448881 |
| 16 | exp Academic Medical Centers/ | 40032 |
| 17 | exp Intensive Care Units/ | 95727 |
| 18 | exp Medicine/ and (architect* or building? or department? or environment* or hospital or hospitals or landscape? or ward? or unit? or floor? or hospitali#ation? or facility or facilities or room or rooms or roomed or rooming).mp. | 179047 |
| 19 | Health Facilities/ | 29327 |
| 20 | Hospital Bed Capacity/ | 2372 |
| 21 | exp Hospital Units/ | 265470 |
| 22 | Hospitals, Proprietary/ | 138101 |
| 23 | Universal Design/ | 191 |
| 24 | Waiting Rooms/ | 1401 |
| 25 | architect*.in,hw. | 14211 |
| 26 | (general adj3 (department? or ward? or unit? or floor? or hospitali#ation?)).mp. | 6846 |
| 27 | (healing adj1 (place or places)).mp. | 18 |
| 28 | (healing adj1 (space or spaces)).mp. | 35 |
| 29 | (health* adj1 environment?).ti,ab,jw. | 3604 |
| 30 | ((health care or healthcare) adj1 design???).mp. | 685 |
| 31 | ((health care or healthcare) adj1 setting?).mp. | 11564 |
| 32 | (healthy adj1 (place? or space?)).mp. | 68 |
| 33 | (hospital? adj3 (department? or ward? or unit? or floor? or hospitali#ation?)).mp. | 29875 |
| 34 | (in-patient?? adj3 room?).mp. | 286 |
| 35 | (inpatient?? adj3 room?).mp. | 342 |
| 36 | (patient?? adj3 room?).mp. | 2941 |
| 37 | (public? adj2 area?).mp. | 956 |
| 38 | (surgical adj3 (department? or ward? or unit? or floor? or hospitali#ation?)).mp. | 9521 |
| 39 | (medical adj3 (department? or ward? or unit? or floor? or hospitali#ation?)).mp. | 13132 |
| 40 | or/1-39 [ Hospitals or Health Care Environment ] | 631327 |
| 41 | Nature/ | 2058 |
| 42 | Bays/ | 335 |
| 43 | Birds/ | 4946 |
| 44 | Estuaries/ | 675 |
| 45 | Forests/ | 5069 |
| 46 | Gardens/ | 3089 |
| 47 | Horticulture/ | 313 |
| 48 | Horticultural Therapy/ | 74 |
| 49 | Lakes/ | 2063 |
| 50 | "Oceans and Seas"/ | 6650 |
| 51 | Plants/ | 11331 |
| 52 | Ponds/ | 596 |
| 53 | Rivers/ | 3720 |
| 54 | exp Songbirds/ | 1482 |
| 55 | Trees/ | 5087 |
| 56 | Vocalization, Animal/ | 3084 |
| 57 | Wilderness/ | 403 |
| 58 | exp Auditory Perception/ and exp Nature/ | 2841 |
| 59 | (access* adj2 natural).mp. | 105 |
| 60 | (access* adj2 nature).mp. | 106 |
| 61 | (acoust* adj3 access* adj3 (nature or natural)).mp. | 0 |
| 62 | (audib* adj3 access* adj3 (nature or natural)).mp. | 0 |
| 63 | (auditor* adj3 access* adj3 (nature or natural)).mp. | 1 |
| 64 | (commun??? adj2 nature).mp. | 152 |
| 65 | (biophili?? adj2 design*).mp. | 8 |
| 66 | (biophili?? adj2 imag*).mp. | 2 |
| 67 | (contact* adj2 nature).mp. | 159 |
| 68 | (experienc* adj2 nature).mp. | 208 |
| 69 | (green adj2 belt*).mp. | 14 |
| 70 | green infrastructur*.mp. | 41 |
| 71 | ((hear or hears or hearing or heard) adj3 (nature or natural)).mp. | 108 |
| 72 | ((hear or hears or hearing or heard) adj3 animal?).mp. | 162 |
| 73 | ((hear or hears or hearing or heard) adj3 bird?).mp. | 20 |
| 74 | ((hear or hears or hearing or heard) adj3 rain*).mp. | 2 |
| 75 | ((hear or hears or hearing or heard) adj3 water).mp. | 11 |
| 76 | ((hear or hears or hearing or heard) adj3 wind?).mp. | 11 |
| 77 | (hear* adj3 access* adj3 (nature or natural)).mp. | 1 |
| 78 | (listen* adj3 (nature or natural)).mp. | 95 |
| 79 | (listen* adj3 animal?).mp. | 13 |
| 80 | (listen* adj3 bird?).mp. | 5 |
| 81 | (listen* adj3 rain*).mp. | 2 |
| 82 | (listen* adj3 water?).mp. | 5 |
| 83 | (listen* adj3 wind?).mp. | 1 |
| 84 | (natural adj2 environ*).mp. | 2870 |
| 85 | (natural adj2 imag*).mp. | 439 |
| 86 | (natural adj2 land*).mp. | 187 |
| 87 | (natural adj2 light*).mp. | 320 |
| 88 | (natural adj2 scene*).mp. | 410 |
| 89 | (natural adj2 setting*).mp. | 770 |
| 90 | (natural adj2 sound?).mp. | 220 |
| 91 | (natural adj2 spac*).mp. | 95 |
| 92 | (natural adj2 view*).mp. | 176 |
| 93 | (natural adj2 virtual*).mp. | 22 |
| 94 | (natural adj1 world?).mp. | 260 |
| 95 | (nature adj2 environ*).mp. | 197 |
| 96 | (nature adj2 imag*).mp. | 114 |
| 97 | (nature adj2 landscape?).mp. | 17 |
| 98 | (nature adj2 scene*).mp. | 38 |
| 99 | (nature adj2 simulat*).mp. | 39 |
| 100 | (nature?? adj2 sound?).mp. | 74 |
| 101 | (nature adj2 spac??).mp. | 37 |
| 102 | (nature adj2 stimul*).mp. | 68 |
| 103 | nature-theme?.mp. | 5 |
| 104 | (nature adj1 theme?).mp. | 12 |
| 105 | (nature adj2 view*).mp. | 99 |
| 106 | (nature adj2 virtual*).mp. | 37 |
| 107 | nature-based.mp. | 245 |
| 108 | (nature-based adj2 art?).mp. | 2 |
| 109 | ((near or nearby) adj3 (nature or natural)).mp. | 112 |
| 110 | (outdoor? adj2 (art or arts or artwork*)).mp. | 7 |
| 111 | (outdoor? adj2 imag*).mp. | 6 |
| 112 | (outdoor? adj2 photo*).mp. | 16 |
| 113 | (physical* adj3 access* adj3 (nature or natural)).mp. | 3 |
| 114 | (scenic adj2 view*).mp. | 3 |
| 115 | (visual* adj3 access* adj3 (nature or natural)).mp. | 1 |
| 116 | airscape?.mp. | 0 |
| 117 | artwork?.mp. | 508 |
| 118 | balconies.mp. | 45 |
| 119 | balcony.mp. | 82 |
| 120 | biophili???.mp. | 58 |
| 121 | birdsong?.mp. | 100 |
| 122 | "blue space?".mp. | 68 |
| 123 | bluespace?.mp. | 6 |
| 124 | cloudscape?.mp. | 0 |
| 125 | countryside?.mp. | 565 |
| 126 | courtyard?.mp. | 80 |
| 127 | daylight*.mp. | 963 |
| 128 | ecotherap*3.mp. | 14 |
| 129 | fauna.mp. | 1848 |
| 130 | "fine art?".mp. | 153 |
| 131 | flower?.mp. | 4677 |
| 132 | flowerbed?.mp. | 6 |
| 133 | (forest? not ("causal forest?" or "forest classifier?" or "forest plot?" or "random* forest?" or "wake forest" or "decision* forest?")).mp. | 9976 |
| 134 | (garden or gardens).mp. | 2624 |
| 135 | (green adj2 space?).mp. | 677 |
| 136 | greenbelt?.mp. | 9 |
| 137 | greenery.mp. | 89 |
| 138 | greenspace?.mp. | 161 |
| 139 | "green space?".mp. | 644 |
| 140 | horticultur*.mp. | 770 |
| 141 | ((landscape or landscapes or landscaping) not (landscape? adj2 (biologic* or regulatory or chang* or cultural* or genetic* or genomic* or global* or practice? or practise? or treatment? or trial? or national or online or economic* or online or techn* or fraught or risk? or contact or test*3 or service? or tele* or policy or politic* or remediat* or health* or institution* or insur* or underemploy* or unemploy*))).mp. | 11862 |
| 142 | (mural or murals).mp. | 1615 |
| 143 | non-city.mp. | 8 |
| 144 | non-urban.mp. | 314 |
| 145 | non-window*.mp. | 3 |
| 146 | nonwindow*.mp. | 3 |
| 147 | "open space?".mp. | 569 |
| 148 | (outdoor? adj3 (life or panoram* or scene??? or space? or view? or vista?)).mp. | 357 |
| 149 | "outside world".mp. | 432 |
| 150 | (panoramic adj2 (scen* or view? or vista?)).mp. | 198 |
| 151 | parkland?.mp. | 324 |
| 152 | phytotherap*3.mp. | 2633 |
| 153 | (plant? adj1 (living or growing)).mp. | 325 |
| 154 | "public space?".mp. | 909 |
| 155 | riverscape?.mp. | 8 |
| 156 | (roof or roofs or rooftop?).mp. | 2245 |
| 157 | scenery.mp. | 194 |
| 158 | seascape?.mp. | 77 |
| 159 | (terrace or terraces).mp. | 204 |
| 160 | (vista and (nature or natural)).mp. | 36 |
| 161 | (vistas and (nature or natural)).mp. | 14 |
| 162 | waterscape?.mp. | 10 |
| 163 | wilderness??.mp. | 1051 |
| 164 | (wilds or wilderness).mp. | 1065 |
| 165 | ((window or windows) not ((spss adj3 windows) or (statistics adj2 windows) or (time adj1 window?) or time-window? or "time window" or (ibm adj3 windows))).mp. | 22615 |
| 166 | ((window or windows) not (hour-window? or minute-window? or (dissection* adj1 window?))).mp. | 26474 |
| 167 | ((window or windows) not (window? adj1 opportunit*)).mp. | 26649 |
| 168 | window-side?.mp. | 6 |
| 169 | windowside?.mp. | 0 |
| 170 | woods.mp. | 594 |
| 171 | Art/ | 6201 |
| 172 | Paintings/ | 1927 |
| 173 | or/41-172 [ Natural Environment & related terms or how to see them ] | 125683 |
| 174 | 40 and 173 [ Hospitals + Nature ] | 10762 |
| 175 | exp Patients/ | 1004432 |
| 176 | exp Hospitalization/ | 113113 |
| 177 | Length of Stay/ | 67649 |
| 178 | Patient-Centered Care/ | 48155 |
| 179 | exp Patient Outcome Assessment/ | 232557 |
| 180 | Patient Readmission/ | 9144 |
| 181 | (patient?? or inpatient??).mp. | 2560366 |
| 182 | ((person?? or client?) adj2 (centered or centred or centric)).mp. | 8623 |
| 183 | (hospital? adj3 (admit* or admis*)).mp. | 97948 |
| 184 | (hospital? adj3 (readmit* or readmis*)).mp. | 23848 |
| 185 | (hospital? adj3 (revisit* or re-visit*)).mp. | 147 |
| 186 | (length adj2 stay*3).mp. | 71797 |
| 187 | hospitali#ed.mp. | 50527 |
| 188 | institutionali#ed.mp. | 6293 |
| 189 | or/175-188 [ Patients & related terms ] | 2720072 |
| 190 | 174 and 189 [ Hospitals + Nature + Patients ] | 4600 |
| 191 | exp Qualitative Research/ | 67917 |
| 192 | exp Communication Barriers/ | 1352 |
| 193 | "Surveys and Questionnaires"/ | 290881 |
| 194 | Evaluation Study/ | 8912 |
| 195 | Feasibility Studies/ | 46965 |
| 196 | Grounded Theory/ | 11089 |
| 197 | Health Care Surveys/ | 4576 |
| 198 | Health Impact Assessment/ | 2450 |
| 199 | Health Knowledge, Attitudes, Practice/ | 9855 |
| 200 | "Health Services Needs and Demand"/ | 77507 |
| 201 | Hermeneutics/ | 564 |
| 202 | Interview/ | 157103 |
| 203 | Narration/ | 5478 |
| 204 | Narrative Medicine/ | 200 |
| 205 | Needs Assessment/ | 7412 |
| 206 | Nursing Methodology Research/ | 1601 |
| 207 | Observational Study/ | 85193 |
| 208 | Patient Education as Topic/ | 32235 |
| 209 | Patient Health Questionnaire/ | 1243 |
| 210 | Patient Reported Outcome Measures/ | 1547 |
| 211 | Patient Satisfaction/ | 57554 |
| 212 | Personal Narrative/ | 7072 |
| 213 | Personal Satisfaction/ | 48071 |
| 214 | Pilot Projects/ | 20430 |
| 215 | exp Program Evaluation/ | 10205 |
| 216 | ((discourse* or discurs*) adj3 analys#s).mp. | 4059 |
| 217 | ((purpos* adj4 sampl*) or (focus adj group*)).mp. | 59762 |
| 218 | (account or accounts or unstructured or open-ended or open ended or text* or narrative*).mp. | 247912 |
| 219 | (action research or cooperative inquir* or co operative inquir* or co- operative inquir*).mp. | 4875 |
| 220 | (constant adj (comparative or comparison)).mp. | 4642 |
| 221 | (corbin* adj2 strauss*).mp. | 382 |
| 222 | (emic or etic or hermeneutic* or heuristic* or semiotic* or (data adj1 saturat*) or participant observ*).mp. | 19324 |
| 223 | (evaluat* adj2 (study or studies)).mp. | 90946 |
| 224 | (field adj (study or studies or research)).mp. | 6577 |
| 225 | (grounded adj (theor* or study or studies or research or analys#s)).mp. | 12896 |
| 226 | (humanistic or existential or experiential or paradigm*).mp. | 62480 |
| 227 | (life stor* or women* stor*).mp. | 1535 |
| 228 | (life world or life-world or conversation analys#s or personal experience* or theoretical saturation).mp. | 36267 |
| 229 | ((lived or life) adj experience*).mp. | 12542 |
| 230 | (merleau adj ponty*).mp. | 257 |
| 231 | (observational adj (study or studies or research)).mp. | 101494 |
| 232 | (social construct* or (postmodern* or post- structural*) or (post structural* or poststructural*) or post modern* or post-modern* or feminis* or interpret*).mp. | 159640 |
| 233 | (survey? or surveyed or surveying).mp. | 509127 |
| 234 | (theme* or thematic).mp. | 105062 |
| 235 | (van adj kaam*).mp. | 35 |
| 236 | (van adj manen*).mp. | 490 |
| 237 | biographical method?.mp. | 34 |
| 238 | cluster sampl*.mp. | 4278 |
| 239 | colaizzi*.mp. | 878 |
| 240 | content analys#s.mp. | 31903 |
| 241 | ethnograph*.mp. | 12729 |
| 242 | ethnological research.mp. | 2 |
| 243 | ethnonursing.mp. | 109 |
| 244 | foucault*.mp. | 1177 |
| 245 | glaser*.mp. | 384 |
| 246 | heidegger*.mp. | 674 |
| 247 | human science.mp. | 173 |
| 248 | narrative analys#s.mp. | 1642 |
| 249 | narrative review?.mp. | 8514 |
| 250 | observational method*.mp. | 1280 |
| 251 | phenomenol*.mp. | 19574 |
| 252 | qualitative.mp. | 185122 |
| 253 | questionnaire*.mp. | 383515 |
| 254 | spiegelberg*.mp. | 27 |
| 255 | theoretical sampl*.mp. | 733 |
| 256 | (client* adj2 (satisfaction or satisfied or satisfy*)).mp. | 1181 |
| 257 | (content analys* or thematic analys* or narrative analys*).mp. | 59983 |
| 258 | (ethnol* or ethnog* or ethnonurs* or emic or etic).mp. | 26263 |
| 259 | (feasib* adj2 (study or studies)).mp. | 49353 |
| 260 | (Grounded adj5 theor*).mp. | 14188 |
| 261 | (hermeneutic* or phenomenolog* or lived experience*).mp. | 26653 |
| 262 | (integrat* adj1 model?).mp. | 3519 |
| 263 | (meta-ethnog* or metaethnog* or meta-narrat* or metanarrat* or meta-interpret* or metainterpret*).mp. | 868 |
| 264 | (metasynthes* or meta-synthes* or metasummar* or meta-summar* or metastud* or meta-stud*).ti,ab. | 1548 |
| 265 | (mixed adj2 method*).mp. | 25746 |
| 266 | (multiple adj1 perspective?).mp. | 902 |
| 267 | (needs adj2 assessment*).mp. | 9273 |
| 268 | (patient?? adj2 (satisfaction or satisfied or satisfy*)).mp. | 63891 |
| 269 | (person?? adj2 (satisfaction or satisfied or satisfy*)).mp. | 1004 |
| 270 | (personal adj1 (story or stories)).mp. | 535 |
| 271 | (personal adj1 account???).mp. | 597 |
| 272 | (program* adj3 evaluat*).mp. | 16801 |
| 273 | (qualitative adj5 metaanaly*).mp. | 6 |
| 274 | (qualitative adj5 meta-analy*).mp. | 518 |
| 275 | (semantic? adj2 description?).mp. | 60 |
| 276 | (therapeutic adj1 model?).mp. | 364 |
| 277 | (treatment? adj1 model?).mp. | 2087 |
| 278 | (unmet adj2 need?).mp. | 11134 |
| 279 | action research.ti,ab. | 4318 |
| 280 | contextual*.mp. | 29699 |
| 281 | focus group?.mp. | 46570 |
| 282 | frame work*.mp. | 199 |
| 283 | framework*.mp. | 137764 |
| 284 | giorgi*.mp. | 543 |
| 285 | interview?.mp. | 251744 |
| 286 | mixed-method*.mp. | 24927 |
| 287 | multimethod inquir*.mp. | 1 |
| 288 | multi-method inquir*.mp. | 2 |
| 289 | multiperspective?.mp. | 97 |
| 290 | multi-perspective?.mp. | 161 |
| 291 | narrative?.mp. | 40962 |
| 292 | phenomenological*.mp. | 14082 |
| 293 | qualitative*.mp. | 194224 |
| 294 | qualitative.mp. | 185122 |
| 295 | questionnaire?.mp. | 383438 |
| 296 | thematic.mp. | 38514 |
| 297 | theme.mp. | 14849 |
| 298 | themes.mp. | 73281 |
| 299 | or/191-298 [ Qualitative Research or Questionnaires & Related Terms ] | 1914778 |
| 300 | exp Clinical Trial/ | 433062 |
| 301 | Clinical Trials, Phase III as Topic/ | 10903 |
| 302 | Comparative Study/ | 133936 |
| 303 | Controlled Clinical Trial/ | 65347 |
| 304 | Controlled Clinical Trials as Topic/ | 3967 |
| 305 | Cross-Sectional Studies/ | 52834 |
| 306 | Double-Blind Method/ | 53172 |
| 307 | Equivalence Trial/ | 48 |
| 308 | Equivalence Trials as Topic/ | 29 |
| 309 | exp Case-Control Studies/ | 45133 |
| 310 | exp Cohort Studies/ | 213027 |
| 311 | exp Randomized Controlled Trial/ | 213336 |
| 312 | exp Randomized Controlled Trials as Topic/ | 76699 |
| 313 | Longitudinal Studies/ | 34982 |
| 314 | Meta-Analysis as Topic/ | 16891 |
| 315 | Meta-Analysis/ | 72029 |
| 316 | Multicenter Studies as Topic/ | 11165 |
| 317 | Multicenter Study/ | 70583 |
| 318 | Observational Study/ | 85193 |
| 319 | Placebos/ | 105886 |
| 320 | Pragmatic Clinical Trial/ | 564 |
| 321 | Pragmatic Clinical Trials as Topic/ | 76682 |
| 322 | Prospective Studies/ | 98406 |
| 323 | Retrospective Studies/ | 107966 |
| 324 | Systematic Review/ | 143982 |
| 325 | Systematic Reviews as Topic/ | 13019 |
| 326 | Validation Study/ | 22340 |
| 327 | ("phase 1" or "phase1" or "phase I").mp. | 23247 |
| 328 | ("phase 2" or "phase2" or "phase II").mp. | 33846 |
| 329 | ("phase 3" or "phase3" or "phase III").mp. | 29306 |
| 330 | ((multicenter* or multicentre*) adj2 (trial? or study or studies)).mp. | 93569 |
| 331 | ((noninferiority or non-inferiority) adj4 (trial? or study or studies)).mp. | 2754 |
| 332 | ((single or double or triple or treble) adj3 (blind* or mask*)).mp. | 85976 |
| 333 | (case control* adj2 (study or studies)).mp. | 54327 |
| 334 | (comparative adj2 (trial? or study or studies)).mp. | 146755 |
| 335 | (conceal* adj2 allocat*).mp. | 1608 |
| 336 | (controlled adj1 clinical adj2 (trial? or study or studies)).mp. | 80366 |
| 337 | (cross-sectional* adj2 (study or studies)).mp. | 180501 |
| 338 | (equivalen* adj4 (trial? or study or studies)).mp. | 2002 |
| 339 | (evaluation adj1 (study or studies)).mp. | 12421 |
| 340 | (longitudinal* adj2 (study or studies)).mp. | 82699 |
| 341 | (meta-anal* or metanal* or metaanal*).mp. | 121867 |
| 342 | (observational adj2 (trial? or study or studies)).mp. | 107435 |
| 343 | (overview? adj4 (review or reviews)).mp. | 5254 |
| 344 | (pragmatic adj2 (trial? or study or studies)).mp. | 2042 |
| 345 | (prospective* adj2 (study or studies)).mp. | 239901 |
| 346 | (retrospective* adj2 (study or studies)).mp. | 276149 |
| 347 | (superiority adj4 (trial? or study or studies)).mp. | 1566 |
| 348 | (systematic adj4 (review or reviews or overview or overviews)).mp. | 173896 |
| 349 | (validation adj1 (study or studies)).mp. | 25018 |
| 350 | cohort*.mp. | 339130 |
| 351 | placebo*.mp. | 120900 |
| 352 | qualitativ*.mp. | 194253 |
| 353 | quasirandom*.mp. | 101 |
| 354 | random*.mp. | 577322 |
| 355 | research.ti,hw,pt. | 398164 |
| 356 | semiquantitative.mp. | 4330 |
| 357 | or/300-356 [ Studies ] | 2207612 |
| 358 | 299 or 357 [ Qualitative or Quantitative Research ] | 3251731 |
| 359 | 190 and 358 [ Hospitals + Nature + Patients + (Qualitative or Quantitative) ] | 2815 |
| 360 | limit 359 to english language | 2611 |
| 361 | 360 not medline.cr. | 2548 |
| 362 | remove duplicates from 361 | 2532 |
| 363 | limit 362 to (books or business article or chapter or conference abstract or "conference review" or dissertation or press release or working paper or (book or book series or conference proceeding or major reference work or report or trade journal)) | 27 |
| 364 | 362 not 363 | 2505 |
| 365 | 364 not (animal or animals or ape or apes or baboon or baboons or bat or bats or beagle or beagles or bird or birds or boar or boars or bonobo or bonobos or bovine or camel or camels or canine or canines or cat or cats or cattle or chick or chicks or chicken or chickens or chimpanzee or chimpanzees or dog or dogs or dromedary or dromedaries or duck or ducks or equine or equines or feline or felines or ferret or ferrets or frog or frogs or fowl or fowls or goat or goats or hamster or hamsters or hare or hares or hen or hens or horse or horses or lamb or lambs or livestock or macaque or macaques or mandrill or mandrills or mice or mink or minks or monkey or monkeys or mouse or murine or ovine or pig or pigs or piglet or piglets or poultry or porcine or orangutan or orangutans or rabbit or rabbits or rat or rats or rodent or rodents or sheep or swine or tamarin or tamarins or tiger or tigers or veterinary or veterinarian or veterinarians or waterfowl or waterfowls or weasel or weasels or veterinar* or fish or shellfish).ti,jw,hw. | 2414 |
| 366 | 364 and (human* or patient? or man or mankind or men or women or woman or adult*).ti,jw. | 669 |
| 367 | limit 362 to human | 2220 |
| 368 | 365 or 366 or 367 | 2508 |

# CINAHL

| **#** | **Query** | **Limiters/Expanders** | **Last Run Via** | **Results** |
| --- | --- | --- | --- | --- |
|  |  | Limited to academic journals |  | 876 |
| S11 | S4 AND S9 | Limiters - English Language; Exclude MEDLINE records; Human  Expanders - Apply equivalent subjects  Search modes - Boolean/Phrase | Interface - EBSCOhost Research Databases  Search Screen - Advanced Search  Database - CINAHL Complete | 951 |
| S10 | S4 AND S9 | Expanders - Apply equivalent subjects  Search modes - Boolean/Phrase | Interface - EBSCOhost Research Databases  Search Screen - Advanced Search  Database - CINAHL Complete | 2,899 |
| S9 | S7 OR S8 | Expanders - Apply equivalent subjects  Search modes - Boolean/Phrase | Interface - EBSCOhost Research Databases  Search Screen - Advanced Search  Database - CINAHL Complete | 2,963,262 |
| S8 | (MH "Clinical Trial+") OR (MH "Comparative Study") OR (MH "Controlled Clinical Trial") OR (MH "Controlled Clinical Trials as Topic") OR (MH "Cross-Sectional Studies") OR (MH "Double-Blind Method") OR (MH "Equivalence Trial") OR (MH "Equivalence Trials as Topic") OR (MH "Case-Control Studies+") OR (MH "Cohort Studies+") OR (MH "Randomized Controlled Trial+") OR (MH "Randomized Controlled Trials as Topic+") OR (MH "Longitudinal Studies") OR (MH "Meta-Analysis as Topic") OR (MH Meta-Analysis) OR (MH "Multicenter Studies as Topic") OR (MH "Multicenter Study") OR (MH "Observational Study") OR (MH "Observational Studies as Topic") OR (MH Placebos) OR (MH "Pragmatic Clinical Trial") OR (MH "Pragmatic Clinical Trials as Topic") OR (MH "Prospective Studies") OR (MH "Retrospective Studies") OR (MH "Systematic Review") OR (MH "Systematic Reviews as Topic") OR (MH "Validation Studies") OR ("phase 1" OR phase1 OR "phase I") OR ("phase 2" OR phase2 OR "phase II") OR ("phase 3" OR phase3 OR "phase III") OR ((multicenter* OR multicentre*) N2 (trial# OR study OR studies)) OR ((noninferiority OR non-inferiority) N4 (trial# OR study OR studies)) OR ((single OR double OR triple OR treble) N3 (blind* OR mask*)) OR ("case control*" N2 (study OR studies)) OR (comparative N2 (trial# OR study OR studies)) OR (conceal* N2 allocat*) OR (controlled N1 clinical N2 (trial# OR study OR studies)) OR (cross-sectional* N2 (study OR studies)) OR (equivalen* N4 (trial# OR study OR studies)) OR (evaluation N1 (study OR studies)) OR (longitudinal* N2 (study OR studies)) OR (meta-anal* OR metanal* OR metaanal*) OR (observational N2 (trial# OR study OR studies)) OR (overview# N4 (review OR reviews)) OR (pragmatic N2 (trial# OR study OR studies)) OR (prospective* N2 (study OR studies)) OR (retrospective* N2 (study OR studies)) OR (superiority N4 (trial# OR study OR studies)) OR (systematic N4 (review OR reviews OR overview OR overviews)) OR (validation N1 (study OR studies)) OR cohort* OR placebo* OR qualitativ* OR quasirandom* OR random* OR (TI research) OR (MW research) OR semiquantitative | Expanders - Apply equivalent subjects  Search modes - Boolean/Phrase | Interface - EBSCOhost Research Databases  Search Screen - Advanced Search  Database - CINAHL Complete | 2,230,976 |
| S7 | S5 OR S6 | Expanders - Apply equivalent subjects  Search modes - Boolean/Phrase | Interface - EBSCOhost Research Databases  Search Screen - Advanced Search  Database - CINAHL Complete | 1,723,059 |
| S6 | ((discourse* OR discurs*) N3 analys?s) OR ((purpos* N4 sampl*) OR (focus W1 group*)) OR (account OR accounts OR unstructured OR open-ended OR "open ended" OR text* OR narrative*) OR ("action research" OR "cooperative inquir*" OR "co operative inquir*" OR "co- operative inquir*") OR (constant W1 (comparative OR comparison)) OR (corbin* N2 strauss*) OR (emic OR etic OR hermeneutic* OR heuristic* OR semiotic* OR (data N1 saturat*) OR "participant observ*") OR (evaluat* N2 (study OR studies)) OR (field W1 (study OR studies OR research)) OR (grounded W1 (theor* OR study OR studies OR research OR analys?s)) OR (humanistic OR existential OR experiential OR paradigm*) OR ("life stor*" OR "women* stor*") OR ("life world" OR life-world OR "conversation analys" ) OR ((lived OR life) W1 experience*) OR (merleau W1 ponty*) OR (observational W1 (study OR studies OR research)) OR ("social construct*" OR (postmodern* OR "post- structural*") OR ("post structural*" OR poststructural*) OR "post modern*" OR post-modern* OR feminis* OR interpret*) OR (survey# OR surveyed OR surveying) OR (theme* OR thematic) OR (van W1 kaam*) OR (van W1 manen*) OR "biographical method#" OR "cluster sampl*" OR colaizzi* OR "content analys" OR ethnograph* OR "ethnological research" OR ethnonursing OR foucault* OR glaser* OR heidegger* OR "human science" OR "narrative analys" OR "narrative review#" OR "observational method*" OR phenomenol* OR qualitative OR questionnaire* OR spiegelberg* OR "theoretical sampl*" OR (client* N2 (satisfaction OR satisfied OR satisfy*)) OR ("content analys*" OR "thematic analys*" OR "narrative analys*") OR (ethnol* OR ethnog* OR ethnonurs* OR emic OR etic) OR (feasib* N2 (study OR studies)) OR (Grounded N5 theor*) OR (hermeneutic* OR phenomenolog* OR "lived experience*") OR (integrat* N1 model#) OR (meta-ethnog* OR metaethnog* OR meta-narrat* OR metanarrat* OR meta-interpret* OR metainterpret*) OR ((TI metasynthes* OR AB metasynthes*) OR (TI meta-synthes* OR AB meta-synthes*) OR (TI metasummar* OR AB metasummar*) OR (TI meta-summar* OR AB meta-summar*) OR (TI metastud* OR AB metastud*) OR (TI meta-stud* OR AB meta-stud*)) OR (mixed N2 method*) OR (multiple N1 perspective#) OR (needs N2 assessment*) OR (patient## N2 (satisfaction OR satisfied OR satisfy*)) OR (person## N2 (satisfaction OR satisfied OR satisfy*)) OR (personal N1 (story OR stories)) OR (personal N1 account###) OR (program* N3 evaluat*) OR (qualitative N5 metaanaly*) OR (qualitative N5 meta-analy*) OR (semantic# N2 description#) OR (therapeutic N1 model#) OR (treatment# N1 model#) OR (unmet N2 need#) OR (TI "action research" OR AB "action research") OR contextual* OR "focus group#" OR "frame work*" OR framework* OR giorgi* OR interview# OR mixed-method* OR "multimethod inquir*" OR "multi-method inquir*" OR multiperspective# OR multi-perspective# OR narrative# OR phenomenological* OR qualitative* OR qualitative OR questionnaire# OR thematic OR theme OR themes | Expanders - Apply equivalent subjects  Search modes - Boolean/Phrase | Interface - EBSCOhost Research Databases  Search Screen - Advanced Search  Database - CINAHL Complete | 1,707,115 |
| S5 | (MH "Qualitative Research+") OR (MH "Communication Barriers+") OR (MH "Surveys and Questionnaires") OR (MH "Evaluation Studies as Topic") OR (MH "Evaluation Studies") OR (MH "Feasibility Studies") OR (MH "Grounded Theory") OR (MH "Health Care Surveys") OR (MH "Health Impact Assessment") OR (MH "Health Knowledge, Attitudes, Practice") OR (MH "Health Services Needs and Demand") OR (MH Hermeneutics) OR (MH Interview) OR (MH "Interviews as Topic") OR (MH Narration) OR (MH "Narrative Medicine") OR (MH "Needs Assessment") OR (MH "Nursing Methodology Research") OR (MH "Observational Studies as Topic") OR (MH "Observational Study") OR (MH "Patient Education as Topic") OR (MH "Patient Health Questionnaire") OR (MH "Patient Reported Outcome Measures") OR (MH "Patient Satisfaction") OR (MH "Personal Narrative") OR (MH "Personal Satisfaction") OR (MH "Pilot Projects") OR (MH "Program Evaluation+") | Expanders - Apply equivalent subjects  Search modes - Boolean/Phrase | Interface - EBSCOhost Research Databases  Search Screen - Advanced Search  Database - CINAHL Complete | 178,990 |
| S4 | S1 AND S2 AND S3 | Expanders - Apply equivalent subjects  Search modes - Boolean/Phrase | Interface - EBSCOhost Research Databases  Search Screen - Advanced Search  Database - CINAHL Complete | 4,134 |
| S3 | (MH Patients+) OR (MH Hospitalization+) OR (MH "Length of Stay") OR (MH "Patient-Centered Care") OR (MH "Patient Outcome Assessment+") OR (MH "Patient Readmission") OR (patient## OR inpatient##) OR ((person## OR client#) N2 (centered OR centred OR centric)) OR (hospital# N3 (admit* OR admis*)) OR (hospital# N3 (readmit* OR readmis*)) OR (hospital# N3 (revisit* OR re-visit*)) OR (length N2 stay*3) OR hospitali?ed OR institutionali?ed | Expanders - Apply equivalent subjects  Search modes - Boolean/Phrase | Interface - EBSCOhost Research Databases  Search Screen - Advanced Search  Database - CINAHL Complete | 2,419,062 |
| S2 | (MH Art) OR (MH Paintings) OR (MH Sculpture) OR (MH Nature) OR (MH Bays) OR (MH Birds) OR (MH Estuaries) OR (MH Forests) OR (MH Gardens) OR (MH Horticulture) OR (MH "Horticultural Therapy") OR (MH Lakes) OR (MH "Oceans and Seas") OR (MH Plants) OR (MH Ponds) OR (MH Rivers) OR (MH Songbirds+) OR (MH Trees) OR (MH "Vocalization, Animal") OR (MH Wilderness) OR (MH "Auditory Perception+") AND (MH Nature+) OR (access* N2 natural) OR (access* N2 nature) OR (acoust* N3 access* N3 (nature OR natural)) OR (audib* N3 access* N3 (nature OR natural)) OR (auditor* N3 access* N3 (nature OR natural)) OR (commun### N2 nature) OR (biophili## N2 design*) OR (biophili## N2 imag*) OR (contact* N2 nature) OR (experienc* N2 nature) OR (green N2 belt*) OR "green infrastructur*" OR ((hear OR hears OR hearing OR heard) N3 (nature OR natural)) OR ((hear OR hears OR hearing OR heard) N3 animal#) OR ((hear OR hears OR hearing OR heard) N3 bird#) OR ((hear OR hears OR hearing OR heard) N3 rain*) OR ((hear OR hears OR hearing OR heard) N3 water) OR ((hear OR hears OR hearing OR heard) N3 wind#) OR (hear* N3 access* N3 (nature OR natural)) OR (listen* N3 (nature OR natural)) OR (listen* N3 animal#) OR (listen* N3 bird#) OR (listen* N3 rain*) OR (listen* N3 water#) OR (listen* N3 wind#) OR (natural N2 environ*) OR (natural N2 imag*) OR (natural N2 land*) OR (natural N2 light*) OR (natural N2 scene*) OR (natural N2 setting*) OR (natural N2 sound#) OR (natural N2 spac*) OR (natural N2 view*) OR (natural N2 virtual*) OR (natural N1 world#) OR (nature N2 environ*) OR (nature N2 imag*) OR (nature N2 landscape#) OR (nature N2 scene*) OR (nature N2 simulat*) OR (nature## N2 sound#) OR (nature N2 spac##) OR (nature N2 stimul*) OR (nature N2 view*) OR (nature N2 virtual*) OR nature-based OR (nature-based N2 art#) OR ((near OR nearby) N3 (nature OR natural)) OR (outdoor# N2 (art OR arts OR artwork*)) OR (outdoor# N2 imag*) OR (outdoor# N2 photo*) OR (physical* N3 access* N3 (nature OR natural)) OR (scenic N2 view*) OR (visual* N3 access* N3 (nature OR natural)) OR airscape# OR artwork# OR balconies OR balcony OR biophili### OR birdsong# OR "blue space#" OR bluespace# OR cloudscape# OR countryside# OR courtyard# OR daylight* OR ecotherap*3 OR fauna OR "fine art#" OR flower# OR flowerbed# OR (forest# NOT ("causal forest#" OR "forest classifier#" OR "forest plot#" OR "random* forest#" OR "wake forest" OR "decision* forest#")) OR (garden OR gardens) OR (green N2 space#) OR greenbelt# OR greenery OR greenspace# OR "green space#" OR horticultur* OR ((landscape OR landscapes OR landscaping) NOT (landscape# N2 (biologic* OR regulatory OR chang* OR cultural* OR genetic* OR genomic* OR global* OR practice# OR practise# OR treatment# OR trial# OR national OR online OR economic* OR online OR techn* OR fraught OR risk# OR contact OR test*3 OR service# OR tele* OR policy OR politic* OR remediat* OR health* OR institution* OR insur* OR underemploy* OR unemploy*))) OR (mural OR murals) OR non-city OR non-urban OR non-window* OR nonwindow* OR "open space#" OR (outdoor# N3 (life OR panoram* OR scene### OR space# OR view# OR vista#)) OR "outside world" OR (panoramic N2 (scen* OR view# OR vista#)) OR parkland# OR phytotherap*3 OR (plant# N1 (living OR growing)) OR "public space#" OR riverscape# OR (roof OR roofs OR rooftop#) OR scenery OR seascape# OR (terrace OR terraces) OR (vista AND (nature OR natural)) OR (vistas AND (nature OR natural)) OR waterscape# OR wilderness## OR (wilds OR wilderness) OR (((window OR windows) NOT ((spss N2 windows) OR (statistics N2 windows) OR (time N1 window#) OR time-window# OR "time window")) OR (ibm N4 windows)) OR ((window OR windows) NOT (hour-window# OR minute-window# OR (dissection* N1 window#))) OR ((window OR windows) NOT (window# N1 opportunit*)) OR window-side# OR windowside# OR woods | Expanders - Apply equivalent subjects  Search modes - Boolean/Phrase | Interface - EBSCOhost Research Databases  Search Screen - Advanced Search  Database - CINAHL Complete | 118,482 |
| S1 | (MH Environment) AND (physical OR build* OR built OR hospital OR hospitals OR inpatient# OR interior# OR facility OR facilities OR architectur*) OR (MH "Facility Design and Construction") OR (MH "Hospital Design and Construction") OR (MH "Interior Design and Furnishings") OR (MH "Patient’s Rooms") OR (MH Architecture) OR (MH "Bed Occupancy") OR (MH "Birthing Centers") OR (MH "Building Codes") OR (MH "Built Environment") OR (MH "Delivery Rooms") OR (MH "Environment Design") OR (MH "Environmental Medicine") OR (MH "Evidence-Based Facility Design") OR (MH Hospitals+) OR (MH "Academic Medical Centers+") OR (MH "Intensive Care Units+") OR (MH Medicine+) AND (architect* OR building# OR department# OR environment* OR hospital OR hospitals OR landscape# OR ward# OR unit# OR floor# OR hospitali?ation# OR facility OR facilities OR room OR rooms OR roomed OR rooming) OR (MH "Health Facilities, Proprietary") OR (MH "Health Facilities") OR (MH "Health Facility Environment") OR (MH "Hospital Bed Capacity") OR (MH "Hospital Units+") OR (MH "Hospitals, Proprietary") OR (MH "Universal Design") OR (MH "Waiting Rooms") OR architect* OR (general N3 (department# OR ward# OR unit# OR floor# OR hospitali?ation#)) OR (healing N1 (place OR places)) OR (healing N1 (space OR spaces)) OR ((TI health* OR AB health*) N1 (TI environment# OR AB environment#)) ,jw. OR (("health care" OR healthcare) N1 design###) OR (("health care" OR healthcare) N1 setting#) OR (healthy N1 (place# OR space#)) OR (hospital# N3 (department# OR ward# OR unit# OR floor# OR hospitali?ation#)) OR (in-patient## N3 room#) OR (inpatient## N3 room#) OR (patient## N3 room#) OR (public# N2 area#) OR (surgical N3 (department# OR ward# OR unit# OR floor# OR hospitali?ation#)) | Expanders - Apply equivalent subjects  Search modes - Boolean/Phrase | Interface - EBSCOhost Research Databases  Search Screen - Advanced Search  Database - CINAHL Complete | 525,703 |

# Scopus

103 document results

( ( ( INDEXTERMS ( environment ) AND TITLE-ABS-KEY ( physical OR build* OR built OR hospital OR hospitals OR inpatient* OR interior* OR facility OR facilities OR architectur* ) OR INDEXTERMS ( "Facility Design and Construction" ) OR INDEXTERMS ( "Hospital Design and Construction" ) OR INDEXTERMS ( "Interior Design and Furnishings" ) OR INDEXTERMS ( "Patient's Rooms" ) OR INDEXTERMS ( architecture ) OR INDEXTERMS ( "Bed Occupancy" ) OR INDEXTERMS ( "Birthing Centers" ) OR INDEXTERMS ( "Building Codes" ) OR INDEXTERMS ( "Built Environment" ) OR INDEXTERMS ( "Delivery Rooms" ) OR INDEXTERMS ( "Environment Design" ) OR INDEXTERMS ( "Environmental Medicine" ) OR INDEXTERMS ( "Evidence-Based Facility Design" ) OR INDEXTERMS ( hospitals ) OR INDEXTERMS ( "Academic Medical Centers" ) OR INDEXTERMS ( "Intensive Care Units" ) OR INDEXTERMS ( medicine ) AND TITLE-ABS-KEY ( architect* OR building* OR department* OR environment* OR hospital OR hospitals OR landscape* OR ward* OR unit* OR floor* OR hospitali?ation* OR facility OR facilities OR room OR rooms OR roomed OR rooming ) OR INDEXTERMS ( "Health Facilities, Proprietary" ) OR INDEXTERMS ( "Health Facilities" ) OR INDEXTERMS ( "Health Facility Environment" ) OR INDEXTERMS ( "Hospital Bed Capacity" ) OR INDEXTERMS ( "Hospital Units" ) OR INDEXTERMS ( "Hospitals, Proprietary" ) OR INDEXTERMS ( "Universal Design" ) OR INDEXTERMS ( "Waiting Rooms" ) OR TITLE-ABS-KEY ( architect* ) OR TITLE-ABS-KEY ( general W/3 ( department* OR ward* OR unit* OR floor* OR hospitali?ation* ) ) OR TITLE-ABS-KEY ( healing W/1 ( place OR places ) ) OR TITLE-ABS-KEY ( healing W/1 ( space OR spaces ) ) OR TITLE-ABS ( health* W/1 environment* ) OR TITLE-ABS-KEY ( ( "health care" OR healthcare ) W/1 design* ) OR TITLE-ABS-KEY ( ( "health care" OR healthcare ) W/1 setting* ) OR TITLE-ABS-KEY ( healthy W/1 ( place* OR space* ) ) OR TITLE-ABS-KEY ( hospital* W/3 ( department* OR ward* OR unit* OR floor* OR hospitali?ation* ) ) OR TITLE-ABS-KEY ( in-patient* W/3 room* ) OR TITLE-ABS-KEY ( inpatient* W/3 room* ) OR TITLE-ABS-KEY ( patient* W/3 room* ) OR TITLE-ABS-KEY ( public* W/2 area* ) OR TITLE-ABS-KEY ( surgical W/3 ( department* OR ward* OR unit* OR floor* OR hospitali?ation* ) ) ) AND ( ( INDEXTERMS ( nature ) OR INDEXTERMS ( bays ) OR INDEXTERMS ( birds ) OR INDEXTERMS ( estuaries ) OR INDEXTERMS ( forests ) OR INDEXTERMS ( gardens ) OR INDEXTERMS ( horticulture ) OR INDEXTERMS ( "Horticultural Therapy" ) OR INDEXTERMS ( lakes ) OR INDEXTERMS ( "Oceans and Seas" ) OR INDEXTERMS ( plants ) OR INDEXTERMS ( ponds ) OR INDEXTERMS ( rivers ) OR INDEXTERMS ( songbirds ) OR INDEXTERMS ( trees ) OR INDEXTERMS ( "Vocalization, Animal" ) OR INDEXTERMS ( wilderness ) OR INDEXTERMS ( "Auditory Perception" ) AND INDEXTERMS ( nature ) OR TITLE-ABS-KEY ( access* W/2 natural ) OR TITLE-ABS-KEY ( access* W/2 nature ) OR TITLE-ABS-KEY ( acoust* W/3 access* W/3 ( nature OR natural ) ) OR TITLE-ABS-KEY ( audib* W/3 access* W/3 ( nature OR natural ) ) OR TITLE-ABS-KEY ( auditor* W/3 access* W/3 ( nature OR natural ) ) OR TITLE-ABS-KEY ( commun* W/2 nature ) OR TITLE-ABS-KEY ( biophili* W/2 design* ) OR TITLE-ABS-KEY ( biophili* W/2 imag* ) OR TITLE-ABS-KEY ( contact* W/2 nature ) OR TITLE-ABS-KEY ( experienc* W/2 nature ) OR TITLE-ABS-KEY ( green W/2 belt* ) OR TITLE-ABS-KEY ( "green infrastructur*" ) OR TITLE-ABS-KEY ( ( hear OR hears OR hearing OR heard ) W/3 ( nature OR natural ) ) OR TITLE-ABS-KEY ( ( hear OR hears OR hearing OR heard ) W/3 animal* ) OR TITLE-ABS-KEY ( ( hear OR hears OR hearing OR heard ) W/3 bird* ) OR TITLE-ABS-KEY ( ( hear OR hears OR hearing OR heard ) W/3 rain* ) OR TITLE-ABS-KEY ( ( hear OR hears OR hearing OR heard ) W/3 water ) OR TITLE-ABS-KEY ( ( hear OR hears OR hearing OR heard ) W/3 wind* ) OR TITLE-ABS-KEY ( hear* W/3 access* W/3 ( nature OR natural ) ) OR TITLE-ABS-KEY ( listen* W/3 ( nature OR natural ) ) OR TITLE-ABS-KEY ( listen* W/3 animal* ) OR TITLE-ABS-KEY ( listen* W/3 bird* ) OR TITLE-ABS-KEY ( listen* W/3 rain* ) OR TITLE-ABS-KEY ( listen* W/3 water* ) OR TITLE-ABS-KEY ( listen* W/3 wind* ) OR TITLE-ABS-KEY ( natural W/2 environ* ) OR TITLE-ABS-KEY ( natural W/2 imag* ) OR TITLE-ABS-KEY ( natural W/2 land* ) OR TITLE-ABS-KEY ( natural W/2 light* ) OR TITLE-ABS-KEY ( natural W/2 scene* ) OR TITLE-ABS-KEY ( natural W/2 setting* ) OR TITLE-ABS-KEY ( natural W/2 sound* ) OR TITLE-ABS-KEY ( natural W/2 spac* ) OR TITLE-ABS-KEY ( natural W/2 view* ) OR TITLE-ABS-KEY ( natural W/2 virtual* ) OR TITLE-ABS-KEY ( natural W/1 world* ) OR TITLE-ABS-KEY ( nature W/2 environ* ) OR TITLE-ABS-KEY ( nature W/2 imag* ) OR TITLE-ABS-KEY ( nature W/2 landscape* ) OR TITLE-ABS-KEY ( nature W/2 scene* ) OR TITLE-ABS-KEY ( nature W/2 simulat* ) OR TITLE-ABS-KEY ( nature* W/2 sound* ) OR TITLE-ABS-KEY ( nature W/2 spac* ) OR TITLE-ABS-KEY ( nature W/2 stimul* ) OR TITLE-ABS-KEY ( nature W/2 view* ) OR TITLE-ABS-KEY ( nature W/2 virtual* ) OR TITLE-ABS-KEY ( nature-based ) OR TITLE-ABS-KEY ( nature-based W/2 art* ) OR TITLE-ABS-KEY ( ( near OR nearby ) W/3 ( nature OR natural ) ) ) OR ( TITLE-ABS-KEY ( outdoor* W/2 ( art OR arts OR artwork* ) ) OR TITLE-ABS-KEY ( outdoor* W/2 imag* ) OR TITLE-ABS-KEY ( outdoor* W/2 photo* ) OR TITLE-ABS-KEY ( physical* W/3 access* W/3 ( nature OR natural ) ) OR TITLE-ABS-KEY ( scenic W/2 view* ) OR TITLE-ABS-KEY ( visual* W/3 access* W/3 ( nature OR natural ) ) OR TITLE-ABS-KEY ( airscape* ) OR TITLE-ABS-KEY ( artwork* ) OR TITLE-ABS-KEY ( balconies ) OR TITLE-ABS-KEY ( balcony ) OR TITLE-ABS-KEY ( biophili* ) OR TITLE-ABS-KEY ( birdsong* ) OR TITLE-ABS-KEY ( "blue space*" ) OR TITLE-ABS-KEY ( bluespace* ) OR TITLE-ABS-KEY ( cloudscape* ) OR TITLE-ABS-KEY ( countryside* ) OR TITLE-ABS-KEY ( courtyard* ) OR TITLE-ABS-KEY ( daylight* ) OR TITLE-ABS-KEY ( ecotherap*3 ) OR TITLE-ABS-KEY ( fauna ) OR TITLE-ABS-KEY ( "fine art*" ) OR TITLE-ABS-KEY ( flower* ) OR TITLE-ABS-KEY ( flowerbed* ) OR TITLE-ABS-KEY ( forest OR forests ) OR TITLE-ABS-KEY ( garden OR gardens ) OR TITLE-ABS-KEY ( green W/2 space* ) OR TITLE-ABS-KEY ( greenbelt* ) OR TITLE-ABS-KEY ( greenery ) OR TITLE-ABS-KEY ( greenspace* ) OR TITLE-ABS-KEY ( "green space*" ) OR TITLE-ABS-KEY ( horticultur* ) OR TITLE-ABS-KEY ( landscape OR landscapes OR landscaping ) OR TITLE-ABS-KEY ( mural OR murals ) OR TITLE-ABS-KEY ( non-city ) OR TITLE-ABS-KEY ( non-urban ) OR TITLE-ABS-KEY ( non-window* ) OR TITLE-ABS-KEY ( nonwindow* ) OR TITLE-ABS-KEY ( "open space*" ) OR TITLE-ABS-KEY ( outdoor* W/3 ( life OR panoram* OR scene* OR space* OR view* OR vista* ) ) OR TITLE-ABS-KEY ( "outside world" ) ) OR ( TITLE-ABS-KEY ( panoramic W/2 ( scen* OR view* OR vista* ) ) OR TITLE-ABS-KEY ( parkland* ) OR TITLE-ABS-KEY ( phytotherap*3 ) OR TITLE-ABS-KEY ( plant* W/1 ( living OR growing ) ) OR TITLE-ABS-KEY ( "public space*" ) OR TITLE-ABS-KEY ( riverscape* ) OR TITLE-ABS-KEY ( roof OR roofs OR rooftop* ) OR TITLE-ABS-KEY ( scenery ) OR TITLE-ABS-KEY ( seascape* ) OR TITLE-ABS-KEY ( terrace OR terraces ) OR TITLE-ABS-KEY ( vista AND ( nature OR natural ) ) OR TITLE-ABS-KEY ( vistas AND ( nature OR natural ) ) OR TITLE-ABS-KEY ( waterscape* ) OR TITLE-ABS-KEY ( wilderness* ) OR TITLE-ABS-KEY ( wilds OR wilderness ) OR TITLE-ABS-KEY ( window OR windows ) OR TITLE-ABS-KEY ( window-side* ) OR TITLE-ABS-KEY ( windowside* ) OR TITLE-ABS-KEY ( woods ) OR INDEXTERMS ( art ) OR INDEXTERMS ( paintings ) OR INDEXTERMS ( "Pictorial Works as Topic" ) OR INDEXTERMS ( sculpture ) ) ) ) AND ( INDEXTERMS ( patients ) OR INDEXTERMS ( hospitalization ) OR INDEXTERMS ( "Length of Stay" ) OR INDEXTERMS ( "Patient-Centered Care" ) OR INDEXTERMS ( "Patient Outcome Assessment" ) OR INDEXTERMS ( "Patient Readmission" ) OR TITLE-ABS-KEY ( patient* OR inpatient* ) OR TITLE-ABS-KEY ( ( person* OR client* ) W/2 ( centered OR centred OR centric ) ) OR TITLE-ABS-KEY ( hospital* W/3 ( admit* OR admis* ) ) OR TITLE-ABS-KEY ( hospital* W/3 ( readmit* OR readmis* ) ) OR TITLE-ABS-KEY ( hospital* W/3 ( revisit* OR re-visit* ) ) OR TITLE-ABS-KEY ( length W/2 stay*3 ) OR TITLE-ABS-KEY ( hospitali?ed ) OR TITLE-ABS-KEY ( institutionali?ed ) ) ) AND NOT ( INDEX ( medline ) ) AND ( LIMIT-TO ( LANGUAGE , "English" ) ) AND ( LIMIT-TO ( SRCTYPE , "j" ) )

# Web of Science


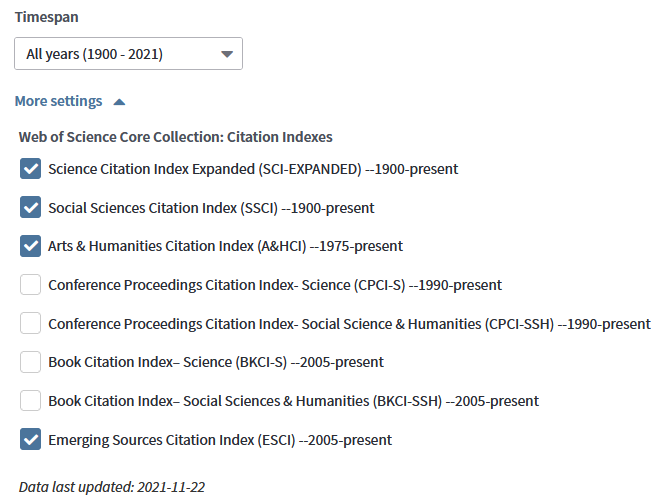


| # 18 | 2,479 | #13  NOT  #14  **Refined by:** **DOCUMENT TYPES:** ( ARTICLE OR EARLY ACCESS ) AND **DOCUMENT TYPES:** ( ARTICLE OR EARLY ACCESS ) AND [excluding] **WEB OF SCIENCE CATEGORIES:** ( PHARMACOLOGY PHARMACY OR IMMUNOLOGY OR VETERINARY SCIENCES OR LINGUISTICS )  Indexes=SCI-EXPANDED, SSCI, A&HCI, ESCI Timespan=All years |
| --- | --- | --- |
| # 17 | 2,595 | #13  NOT  #14  **Refined by:** **DOCUMENT TYPES:** ( ARTICLE OR EARLY ACCESS ) AND **DOCUMENT TYPES:** ( ARTICLE OR EARLY ACCESS )  Indexes=SCI-EXPANDED, SSCI, A&HCI, ESCI Timespan=All years |
| # 16 | 2,595 | #13  NOT  #14  **Refined by:** **DOCUMENT TYPES:** ( ARTICLE OR EARLY ACCESS )  Indexes=SCI-EXPANDED, SSCI, A&HCI, ESCI Timespan=All years |
| # 15 | 2,595 | #13  NOT  #14  Indexes=SCI-EXPANDED, SSCI, A&HCI, ESCI Timespan=All years |
| # 14 | 25,142,153 | PMID=("0*"  or  "1*"  OR  "2*"  OR  "3*"  OR  "4*"  OR  "5*"  OR  "6*"  OR  "7*"  OR  "8*"  OR  "9*")  Indexes=SCI-EXPANDED, SSCI, A&HCI, ESCI Timespan=All years |
| # 13 | 18,485 | #12  AND  #9  Indexes=SCI-EXPANDED, SSCI, A&HCI, ESCI Timespan=All years |
| # 12 | 10,072,424 | #11  OR  #10  Indexes=SCI-EXPANDED, SSCI, A&HCI, ESCI Timespan=All years |
| # 11 | 4,492,099 | (TS=(Quantitative  OR  ("phase 1" OR phase1 OR "phase I")  OR  ("phase 2" OR phase2 OR "phase II")  OR  ("phase 3" OR phase3 OR "phase III")  OR  ((multicenter* OR multicentre*)  NEAR/2  (trial$ OR study OR studies) )  OR  ((noninferiority OR non-inferiority)  NEAR/4  (trial$ OR study OR studies) )  OR  ((single OR double OR triple OR treble)  NEAR/3  (blind* OR mask*) )  OR  ("case control*" NEAR/2 (study OR studies) )  OR  (comparative NEAR/2 (trial$ OR study OR studies) )  OR  (conceal* NEAR/2 allocat*)  OR  (controlled NEAR/1 clinical NEAR/2 (trial$ OR study OR studies) )  OR  (cross-sectional* NEAR/2 (study OR studies) )  OR  (equivalen* NEAR/4 (trial$ OR study OR studies) )  OR  (evaluation NEAR/1 (study OR studies) )  OR  (longitudinal* NEAR/2 (study OR studies) )  OR  (meta-anal* OR metanal* OR metaanal*)  OR  (observational NEAR/2 (trial$ OR study OR studies) )  OR  (overview$ NEAR/4 (review OR reviews) )  OR  (pragmatic NEAR/2 (trial$ OR study OR studies) )  OR  (prospective* NEAR/2 (study OR studies) )  OR  (retrospective* NEAR/2 (study OR studies) )  OR  (superiority NEAR/4 (trial$ OR study OR studies) )  OR  (systematic NEAR/4 (review OR reviews OR overview OR overviews) )  OR  (validation NEAR/1 (study OR studies) )  OR  cohort*  OR  placebo*  OR  quasirandom*  OR  random*  OR  semiquantitative  ))  *AND* **LANGUAGE:**  (English)  *AND*  **DOCUMENT  TYPES:**  (Article)  Indexes=SCI-EXPANDED, SSCI, A&HCI, ESCI Timespan=All years |
| # 10 | 6,930,431 | (TS=("Qualitative  Research"  OR  "Communication  Barriers"  OR  "Health  Care  Surveys"  OR  "Health  Impact  Assessment"  OR  "Health  Knowledge,  Attitudes,  Practice"  OR  "Health  Services  Needs  and  Demand"  OR  Hermeneutics  OR  "Narrative  Medicine"  OR  "Needs  Assessment"  OR  "Nursing  Methodology  Research"  OR  "Patient  Health  Questionnaire"  OR  "Patient  Reported  Outcome  Measures"  OR  "Personal  Narrative"  OR  "Personal  Satisfaction"  OR  "Pilot  Projects"  OR  "Program  Evaluation"  OR  ((discourse* OR discurs*)  NEAR/3  analys?s)  OR  ((purpos* NEAR/4 sampl*)  OR  (focus NEAR/0 group*) )  OR  (account OR accounts OR unstructured OR open-ended OR "open ended" OR text* OR narrative*)  OR  ("action research" OR "cooperative inquir*" OR "co operative inquir*" OR "co- operative inquir*")  OR  (constant NEAR/0 (comparative OR comparison) )  OR  (corbin* NEAR/2 strauss*)  OR  (emic OR etic OR hermeneutic* OR heuristic* OR semiotic* OR (data NEAR/1 saturat*)  OR  "participant  observ*")  OR  (evaluat* NEAR/2 (study OR studies) )  OR  (field NEAR/0 (study OR studies OR research) )  OR  (grounded NEAR/0 (theor* OR study OR studies OR research OR analys?s) )  OR  (humanistic OR existential OR experiential OR paradigm*)  OR  ("life stor*" OR "women* stor*")  OR  ("life world" OR life-world OR "conversation analys")  OR  ((lived OR life)  NEAR/0  experience*)  OR  (merleau NEAR/0 ponty*)  OR  (observational NEAR/0 (study OR studies OR research) )  OR  ("social construct*" OR (postmodern* OR "post- structural*")  OR  ("post structural*" OR poststructural*)  OR  "post  modern*"  OR  post-modern*  OR  feminis*  OR  interpret*)  OR  (van NEAR/0 kaam*)  OR  (van NEAR/0 manen*)  OR  "biographical  method$"  OR  "cluster  sampl*"  OR  colaizzi*  OR  "content  analys"  OR  ethnograph*  OR  "ethnological  research"  OR  ethnonursing  OR  foucault*  OR  glaser*  OR  heidegger*  OR  "human  science"  OR  "narrative  analys"  OR  "narrative  review$"  OR  "observational  method*"  OR  phenomenol*  OR  spiegelberg*  OR  "theoretical  sampl*"  OR  (client* NEAR/2 (satisfaction OR satisfied OR satisfy*) )  OR  ("content analys*" OR "thematic analys*" OR "narrative analys*")  OR  (ethnol* OR ethnog* OR ethnonurs* OR emic OR etic)  OR  (feasib* NEAR/2 (study OR studies) )  OR  (grounded NEAR/5 theor*)  OR  (hermeneutic* OR phenomenolog* OR "lived experience*")  OR  (integrat* NEAR/1 model$)  OR  (meta-ethnog* OR metaethnog* OR meta-narrat* OR metanarrat* OR meta-interpret* OR metainterpret*)  OR  (metasynthes* OR meta-synthes* OR metasummar* OR meta-summar* OR metastud* OR meta-stud*)  OR  (mixed NEAR/2 method*)  OR  (multiple NEAR/1 perspective$)  OR  (needs NEAR/2 assessment*)  OR  (patient$ NEAR/2 (satisfaction OR satisfied OR satisfy*) )  OR  (person$ NEAR/2 (satisfaction OR satisfied OR satisfy*) )  OR  (personal NEAR/1 (story OR stories) )  OR  (personal NEAR/1 account*)  OR  (program* NEAR/3 evaluat*)  OR  (semantic$ NEAR/2 description$)  OR  (therapeutic NEAR/1 model$)  OR  (treatment$ NEAR/1 model$)  OR  (unmet NEAR/2 need$)  OR  "action  research"  OR  contextual*  OR  "focus  group$"  OR  "frame  work*"  OR  framework*  OR  giorgi*  OR  interview$  OR  mixed-method*  OR  "multimethod  inquir*"  OR  "multi-method  inquir*"  OR  multiperspective$  OR  multi-perspective$  OR  narrative$  OR  qualitative*  OR  questionnaire$  OR  survey$  OR  thematic  OR  theme  OR  themes  ))  *AND* **LANGUAGE:**  (English)  *AND*  **DOCUMENT  TYPES:**  (Article)  Indexes=SCI-EXPANDED, SSCI, A&HCI, ESCI Timespan=All years |
| # 9 | 29,997 | #8  AND  #7  Indexes=SCI-EXPANDED, SSCI, A&HCI, ESCI Timespan=All years |
| # 8 | 5,012,836 | (TS=(Patients  OR  Hospitalization  OR  "Length  of  Stay"  OR  "Patient-Centered  Care"  OR  "Patient  Outcome  Assessment"  OR  "Patient  Readmission"  OR  (patient* OR inpatient*)  OR  ((person* OR client$)  NEAR/2  (centered OR centred OR centric) )  OR  (hospital$ NEAR/3 (admit* OR admis*) )  OR  (hospital$ NEAR/3 (readmit* OR readmis*) )  OR  (hospital$ NEAR/3 (revisit* OR re-visit*) )  OR  (length NEAR/2 stay*3)  OR  hospitali?ed  OR  institutionali?ed  ))  *AND* **LANGUAGE:**  (English)  *AND*  **DOCUMENT  TYPES:**  (Article)  Indexes=SCI-EXPANDED, SSCI, A&HCI, ESCI Timespan=All years |
| # 7 | 194,021 | #6  AND  #1  Indexes=SCI-EXPANDED, SSCI, A&HCI, ESCI Timespan=All years |
| # 6 | 5,097,659 | #5  OR  #4  OR  #3  OR  #2  Indexes=SCI-EXPANDED, SSCI, A&HCI, ESCI Timespan=All years |
| # 5 | 1,940,151 | (TS=((outdoor$ NEAR/2 (art OR arts OR artwork*) )  OR  (outdoor$ NEAR/2 imag*)  OR  (outdoor$ NEAR/2 photo*)  OR  (physical* NEAR/3 access* NEAR/3 (nature OR natural) )  OR  (scenic NEAR/2 view*)  OR  (visual* NEAR/3 access* NEAR/3 (nature OR natural) )  OR  airscape$  OR  artwork$  OR  balconies  OR  balcony  OR  biophili*  OR  birdsong$  OR  "blue  space$"  OR  bluespace$  OR  cloudscape$  OR  countryside$  OR  courtyard$  OR  daylight*  OR  ecotherap*3  OR  fauna  OR  "fine  art$"  OR  flower$  OR  flowerbed$  OR  (forest$ NOT ("causal forest$" OR "forest classifier$" OR "forest plot$" OR "random* forest$" OR "wake forest" OR "decision* forest$") )  OR  (garden OR gardens)  OR  (green NEAR/2 space$)  OR  greenbelt$  OR  greenery  OR  greenspace$  OR  "green  space$"  OR  horticultur*  OR  ((landscape OR landscapes OR landscaping)  NOT  (landscape$ NEAR/2 (biologic* OR regulatory OR chang* OR cultural* OR genetic* OR genomic* OR global* OR practice$ OR practise$ OR treatment$ OR trial$ OR national OR online OR economic* OR online OR techn* OR fraught OR risk$ OR contact OR test*3 OR service$ OR tele* OR policy OR politic* OR remediat* OR health* OR institution* OR insur* OR underemploy* OR unemploy*) ))  OR  (mural OR murals)  OR  non-city  OR  non-urban  OR  non-window*  OR  nonwindow*  OR  "open  space$"  OR  (outdoor$ NEAR/3 (life OR panoram* OR scene* OR space$ OR view$ OR vista$) )  OR  "outside  world"  OR  (panoramic NEAR/2 (scen* OR view$ OR vista$) )  OR  parkland$  OR  phytotherap*3  OR  (plant$ NEAR/1 (living OR growing) )  OR  "public  space$"  OR  riverscape$  OR  (roof OR roofs OR rooftop$)  OR  scenery  OR  seascape$  OR  (terrace OR terraces)  OR  (vista AND (nature OR natural) )  OR  (vistas AND (nature OR natural) )  OR  waterscape$  OR  wilderness*  OR  (wilds OR wilderness)  OR  (((window OR windows)  NOT  ((spss NEAR/2 windows)  OR  (statistics NEAR/2 windows)  OR  (time NEAR/1 window$)  OR  time-window$  OR  "time  window"))  OR  (ibm NEAR/4 windows) )  OR  ((window OR windows)  NOT  (hour-window$ OR minute-window$ OR (dissection* NEAR/1 window$) ))  OR  ((window OR windows)  NOT  (window$ NEAR/1 opportunit*) )  OR  window-side$  OR  windowside$  OR  woods  OR  Art  OR  Paintings  OR  "Pictorial  Works  as  Topic"  OR  Sculpture  OR  Sculptures  ))  *AND* **LANGUAGE:**  (English)  *AND*  **DOCUMENT  TYPES:**  (Article)  Indexes=SCI-EXPANDED, SSCI, A&HCI, ESCI Timespan=All years |
| # 4 | 89,818 | (TS=((listen* NEAR/3 (nature OR natural) )  OR  (listen* NEAR/3 animal$)  OR  (listen* NEAR/3 bird$)  OR  (listen* NEAR/3 rain*)  OR  (listen* NEAR/3 water$)  OR  (listen* NEAR/3 wind$)  OR  (natural NEAR/2 environ*)  OR  (natural NEAR/2 imag*)  OR  (natural NEAR/2 land*)  OR  (natural NEAR/2 light*)  OR  (natural NEAR/2 scene*)  OR  (natural NEAR/2 setting*)  OR  (natural NEAR/2 sound$)  OR  (natural NEAR/2 spac*)  OR  (natural NEAR/2 view*)  OR  (natural NEAR/2 virtual*)  OR  (natural NEAR/1 world$)  OR  (nature NEAR/2 environ*)  OR  (nature NEAR/2 imag*)  OR  (nature NEAR/2 landscape$)  OR  (nature NEAR/2 scene*)  OR  (nature NEAR/2 simulat*)  OR  (nature* NEAR/2 sound$)  OR  (nature NEAR/2 spac*)  OR  (nature NEAR/2 stimul*)  OR  (nature NEAR/2 view*)  OR  (nature NEAR/2 virtual*)  OR  nature-based  OR  (nature-based NEAR/2 art$)  OR  (("near" OR nearby)  NEAR/3  (nature OR natural) )  ))  *AND* **LANGUAGE:**  (English)  *AND*  **DOCUMENT  TYPES:**  (Article)  Indexes=SCI-EXPANDED, SSCI, A&HCI, ESCI Timespan=All years |
| # 3 | 1,231 | (TS=(((hear OR hears OR hearing OR heard)  NEAR/3  (nature OR natural) )  OR  ((hear OR hears OR hearing OR heard)  NEAR/3  animal$)  OR  ((hear OR hears OR hearing OR heard)  NEAR/3  bird$)  OR  ((hear OR hears OR hearing OR heard)  NEAR/3  rain*)  OR  ((hear OR hears OR hearing OR heard)  NEAR/3  water)  OR  ((hear OR hears OR hearing OR heard)  NEAR/3  wind$)  ))  *AND* **LANGUAGE:**  (English)  *AND*  **DOCUMENT  TYPES:**  (Article)  Indexes=SCI-EXPANDED, SSCI, A&HCI, ESCI Timespan=All years |
| # 2 | 3,859,642 | (TS=(Nature  OR  Bays  OR  Birds  OR  Estuaries  OR  Forests  OR  Gardens  OR  Horticulture  OR  "Horticultural  Therapy"  OR  Lakes  OR  Oceans  OR  Sea  OR  Seas  OR  Plants  OR  Ponds  OR  Rivers  OR  Songbirds  OR  Trees  OR  Wilderness  OR  (access* NEAR/2 natural)  OR  (access* NEAR/2 nature)  OR  (acoust* NEAR/3 access* NEAR/3 (nature OR natural) )  OR  (audib* NEAR/3 access* NEAR/3 (nature OR natural) )  OR  (auditor* NEAR/3 access* NEAR/3 (nature OR natural) )  OR  (commun* NEAR/2 nature)  OR  (biophili* NEAR/2 design*)  OR  (biophili* NEAR/2 imag*)  OR  (contact* NEAR/2 nature)  OR  (experienc* NEAR/2 nature)  OR  (green NEAR/2 belt*)  OR  "green  infrastructur*"  ))  *AND* **LANGUAGE:**  (English)  *AND*  **DOCUMENT  TYPES:**  (Article)  Indexes=SCI-EXPANDED, SSCI, A&HCI, ESCI Timespan=All years |
| # 1 | 1,640,371 | (TS=(Environment AND (physical OR build* OR built OR hospital OR hospitals OR inpatient$ OR interior$ OR facility OR facilities OR architectur*)  OR  "Facility  Design"  OR  "Hospital  Design"  OR  "Interior  Design"  OR  "Patient’s  Room*"  OR  Architecture  OR  "Bed  Occupancy"  OR  "Birthing  Centers"  OR  "Building  Codes"  OR  "Built  Environment"  OR  "Delivery  Rooms"  OR  "Environment  Design"  OR  "Environmental  Medicine"  OR  "Evidence-Based  Facility  Design"  OR  Hospitals  OR  "Academic  Medical  Centers"  OR  "Intensive  Care  Units"  OR  Medicine  AND  (architect* OR building$ OR department$ OR environment* OR hospital OR hospitals OR landscape$ OR ward$ OR unit$ OR floor$ OR hospitali?ation$ OR facility OR facilities OR room OR rooms OR roomed OR rooming)  OR  "Health  Facilities,  Proprietary"  OR  "Health  Facilities"  OR  "Health  Facility  Environment"  OR  "Hospital  Bed  Capacity"  OR  "Hospital  Units"  OR  "Hospitals,  Proprietary"  OR  "Universal  Design"  OR  "Waiting  Rooms"  OR  architect*  OR  (general NEAR/3 (department$ OR ward$ OR unit$ OR floor$ OR hospitali?ation$) )  OR  (healing NEAR/1 (place OR places) )  OR  (healing NEAR/1 (space OR spaces) )  OR  (health* NEAR/1 environment$)  ,jw.  OR  (("health care" OR healthcare)  NEAR/1  design*)  OR  (("health care" OR healthcare)  NEAR/1  setting$)  OR  (healthy NEAR/1 (place$ OR space$) )  OR  (hospital$ NEAR/3 (department$ OR ward$ OR unit$ OR floor$ OR hospitali?ation$) )  OR  (in-patient* NEAR/3 room$)  OR  (inpatient* NEAR/3 room$)  OR  (patient* NEAR/3 room$)  OR  (public$ NEAR/2 area$)  OR  (surgical NEAR/3 (department$ OR ward$ OR unit$ OR floor$ OR hospitali?ation$) )  ))  *AND* **LANGUAGE:**  (English)  *AND*  **DOCUMENT  TYPES:**  (Article)  Indexes=SCI-EXPANDED, SSCI, A&HCI, ESCI Timespan=All years |

# JSTOR

Searching is too rudimentary for sophisticated searching. Clients may wish to conduct searches on this database for supplemental information.

# Avery Index

Avery Index to Architectural Periodicals (ProQuest)

(hospital OR hospitals OR healthcare OR "health care" OR "medical center" OR "medical centers") OR su(hospital* OR healthcare* OR medicine OR medical OR clinic*) AND (patient* OR inpatient* OR in-patient) AND (room OR rooms OR window* OR interior OR interiors OR outdoor* OR natur* OR daylight* OR sound* OR architect* OR (interior N2 design*) OR biophil*)

140 results

Filters:

- Journal Article
- English
- interior designers united states. OR clinics england london charing cross hospital maggie's centre. OR health care buildings interior design united states. OR health care buildings. OR health care buildings research. OR royal institute of british architects awards and prizes. OR great britain. national health service. OR hospices england london charing cross hospital maggie's centre. OR health care buildings great britain. OR interior design 21st century. OR health care buildings psychological aspects. OR hospitals history. OR hospitals. OR building design partnership. OR environmental design research. OR health care buildings 21st century. OR health care buildings economic aspects great britain. OR health care buildings interior design. OR hospitals england isle of wight newport saint mary's hospital OR hospitals interior design great britain. OR architectural design health aspects. OR architectural design research. OR buildings health aspects australia melbourne medibank place. OR children's hospitals england london saint thomas's hospital evelina children's hospital. OR clinics scotland dundee ninewell hospital maggie's centre. OR environmental psychology research. OR environmental psychology. OR health care buildings 21st century england london st bartholomew's hospital (barts) maggie's centre barts. OR health care buildings 21st century great britain. OR health care buildings australia. OR health care buildings england london. OR health care buildings interior design psychological aspects. OR health care buildings sociological aspects. OR hospices 21st century england london st bartholomew's hospital (barts) maggie's centre barts. OR hospices scotland dundee ninewell hospital maggie's centre. OR hospitals 19th century alterations and additions england london saint thomas's hospital evelina children's hospital. OR hospitals 21st century england bath circlebath. OR hospitals alterations and additions england london saint bartholomew's hospital. OR hospitals environmental aspects. OR hospitals great britain. OR hospitals interior design psychological aspects. OR hospitals medieval alterations and additions england london st bartholomew's hospital (barts) maggie's centre barts. OR psychiatric hospitals united states. OR research facilities australia ballarat ballarat base hospital ballarat regional integrated cancer centre. OR sanatoriums united states.

# DAAI

Design and Applied Arts Index (ProQuest)

**628 results**

(hospital OR hospitals OR healthcare OR "health care" OR "medical center" OR "medical centers") OR su(hospital* OR healthcare* OR medicine OR medical OR clinic*) AND (patient* OR inpatient* OR in-patient) AND (room OR rooms OR window* OR interior OR interiors OR outdoor* OR natur* OR daylight* OR sound* OR architect* OR (interior N2 design*) OR biophil*)Top of Form

Additional limits:

- Source type: Scholarly Journals
- Language: English
- Article
- design OR healthcare OR interior design OR hospitals OR architecture OR health care OR designers OR patients OR human factors OR collaboration OR ergonomics OR innovations OR mental health OR user-centred design OR design process OR architects OR co-design OR cancer OR usability OR industrial design OR offices OR technology OR culture OR service design OR research methodology OR trends OR ethnography OR interdisciplinary aspects OR public health OR buildings OR cognition & reasoning OR creativity OR decision making OR graphic arts OR installations OR knowledge OR lighting OR user experience OR aesthetics
