# Supplementary material for: The Influence of Exposure to Nature on Inpatient Hospital Stays: A Scoping Review
Source: HERD. 2024 Jan 30;17(2):360–75. doi: 10.1177/19375867231221559 (PMC11080386; doi:10.1177/19375867231221559)
Supplement: Supplemental Material, sj-docx-2-her-10.1177_19375867231221559 - The Influence of Exposure to Nature on Inpatient Hospital Stays: A Scoping Review [file sj-docx-2-her-10.1177_19375867231221559.docx]

Appendix 2: Data Collection Form

| Study Number | |  |
| --- | --- | --- |
| Demographics | |  |
|  | First author |  |
|  | Country |  |
|  | Year |  |
|  | Journal |  |
|  | Study design |  |
| Subject | |  |
|  | Clinical setting |  |
|  | Patient population |  |
|  | Number of patients examined, by group |  |
|  | Interventional Type |  |
|  | Detailed description of intervention |  |
|  | Outcomes studies |  |
| Results | |  |
|  | Effect present (yes/no) |  |
|  | Numerical results |  |
|  | Patient reported results |  |
|  | Study conclusion |  |
|  | Study limitations & next steps |  |
| Miscellaneous | |  |
|  | Any additional data |  |
|  | Useful citations |  |
