# Supplementary material for: The Influence of Exposure to Nature on Inpatient Hospital Stays: A Scoping Review
Source: HERD. 2024 Jan 30;17(2):360–75. doi: 10.1177/19375867231221559 (PMC11080386; doi:10.1177/19375867231221559)
Supplement: Supplemental Material, sj-docx-3-her-10.1177_19375867231221559 - The Influence of Exposure to Nature on Inpatient Hospital Stays: A Scoping Review [file sj-docx-3-her-10.1177_19375867231221559.docx]

Appendix 3. Table of included studies.

| **Study Number** | **Author** | **Journal** | **Year** |
| --- | --- | --- | --- |
| 1 | Aburas | Health Environments Research and Design Journal | 2017 |
| 2 | Ali Khan | Gesunde Pflanzen | 2016 |
| 3 | Allah Yar | Urban Forestry and Urban Greening | 2020 |
| 4 | Anaker | Health Environments Research and Design Journal | 2019 |
| 5 | Aomura | Frontiers in Medicine | 2021 |
| 6 | Arenson | Journal of Thoracic and Cardiovascular Surgery | 2013 |
| 7 | Bahonar | Holistic Nursing Practice | 2019 |
| 8 | Bahonar | Journal of Complementary and Integrative Medicine | 2019 |
| 9 | Bauer | Alternative Therapies | 2011 |
| 10 | Benedetti | Journal of Affective Disorders | 2001 |
| 11 | Berg | Journal of Aging and Environment | 2020 |
| 12 | Bielinins | International Journal of Environmental Research and Public Health | 2019 |
| 13 | Chiu | International Journal of Gerontology | 2017 |
| 14 | Choi | Building and Environment | 2012 |
| 15 | Emami | Complementary Therapies in Clinical Practice | 2018 |
| 16 | Finkel | Health Environments Research and Design Journal | 2021 |
| 17 | Gao | Building and Environment | 2020 |
| 18 | Gerber | Frontiers in Medicine | 2019 |
| 19 | Ghezeljeh | Complementary Therapies in Clinical Practice | 2017 |
| 20 | Ghezeljeh | Nursing and Midwifery Studies | 2018 |
| 21 | Iwamoto | Journal of the American Medical Directors Association | 2020 |
| 22 | Jawed | Heart and Lung | 2021 |
| 23 | Keep | Anaesthesia | 1980 |
| 24 | Koh | Heart and Vessels | 2019 |
| 25 | Kohn | Critical Care Medicine | 2013 |
| 26 | Kucher | Complementary Therapies in Medicine | 2020 |
| 27 | Lu | International Journal of Environmental Research and Public Health | 2021 |
| 28 | Mackrill | Journal of Environmental Psychology | 2013 |
| 29 | Mihandoust | International Journal of Environmental Research and Public Health | 2021 |
| 30 | Park | HortTechnology | 2008 |
| 31 | Pati | Health Environments Research and Design Journal | 2016 |
| 32 | Pearson | Health Environments Research and Design Journal | 2019 |
| 33 | Saadatmand | Pain Management Nursing | 2014 |
| 34 | Saadatmand | International Journal of Nursing Studies | 2013 |
| 35 | Shepley | Health Environments Research and Design Journal | 2011 |
| 36 | Smonig | Annals of Intensive Care | 2019 |
| 37 | Timmerman | Scandinavian Journal of Caring Sciences | 2014 |
| 38 | Ulrich | Science | 1984 |
| 39 | Wang | International Journal for Quality in Health Care | 2019 |
| 40 | Wunsch | Critical Care | 2011 |
| 41 | Zaki | Acta Medica International | 2016 |
